# Supplementary material for: Game of Stones: feasibility randomised controlled trial of how to engage men with obesity in text message and incentive interventions for weight loss
Source: BMJ Open. 2020 Feb 25;10(2):e032653. doi: 10.1136/bmjopen-2019-032653 (PMC7045214; doi:10.1136/bmjopen-2019-032653)
Supplement: Supplementary data [file bmjopen-2019-032653supp001.pdf]

Online supplement 1: Incentive strategy contingent on meeting weight loss targets over 12 months

	Money secured	Money lost
3 months: £50 – target 5% weight loss		
<5%	£0	£50
≥5%	£50	£0
6 months: £150 – target 10%		
<5%	£0	£150
5%	£75	£75
6%	£90	£60
7%	£105	£45
8%	£120	£30
9%	£135	£15
≥10%	£150	£0
12 months: £200 – target 10%		
<5%	£0	£200
5%	£100	£100
6%	£120	£80
7%	£140	£60
8%	£160	£40
9%	£180	£20
≥10%	£200	£0

Dombrowski SU, *et al. BMJ Open* 2020; 10:e032653. doi: 10.1136/bmjopen-2019-032653

Online supplement 2 Intervention engagement over time for narrative texts and study webpage

	0-3 months		3-6 months		6-12 months	
	SMS + I N=36	SMS only N=33	SMS + I N=36	SMS only N=33	SMS + I N=36	SMS only N=33
Replies to texts, count	289	81	10	6	28	11
Replies to texts, mean (SD)	8.0 (24.2)	2.5 (7.9)	0.3 (0.7)	0.3 (1.0)	0.8 (3.3)	0.3 (1.0)
**Text replies, n (%)**						
0 replies	21 (58.3)	23 (69.7)	30 (83.3)	31 (93.9)	28 (77.8)	28 (84.8)
1-5 replies	9 (25.0)	7 (22.3)	6 (16.7)	2 (6.1)	7 (19.4)	5 (15.2)
6-10 replies	2 (5.6)	1 (3.0)	0 (0.0)	0 (0.0)	0 (0.0)	0 (0.0)
11-15 replies	0 (0.0)	0 (0.0)	0 (0.0)	0 (0.0)	0 (0.0)	0 (0.0)
16-20 replies	1 (2.8)	1 (3.0)	0 (0.0)	0 (0.0)	0 (0.0)	0 (0.0)
>20 replies	3 (8.3)	1 (3.0)	0 (0.0)	0 (0.0)	1 (2.8)	0 (0.0)

Dombrowski SU, *et al. BMJ Open* 2020; 10:e032653. doi: 10.1136/bmjopen-2019-032653

Stopping texts, n (%)	1 (2.8)	2 (6.1)	3 (8.3)	3 (9.1)	1 (2.8)	1 (3.0)
Withdrawal due to texts (self-report)	2 (5.6)	2 (6.1)	0 (0.0)	0 (0.0)	0 (0.0)	0 (0.0)
**Participants using website self-monitoring features, n (%)**						
Weight	8 (22.2)	5 (15.2)	6 (16.7)	3 (9.1)	4 (11.1)	3 (9.1)
Steps	10 (27.8)	6 (18.2)	7 (19.4)	2 (6.1)	3 (8.3)	2 (6.1)
Waistline	4 (11.1)	3 (9.1)	3 (8.3)	0 (0.0)	1 (2.8)	0 (0.0)
Belt notches	2 (5.6)	0 (0.0)	2 (5.6)	0 (0.0)	0 (0.0)	0 (0.0)
**Number self-monitoring features used, n (%)**						
None	24 (66.7)	26 (78.8)	28 (77.8)	30 (90.1)	31 (86.1)	29 (87.9)
One	5 (13.9)	2 (6.1)	2 (5.6)	1 (3.1)	3 (8.3)	3 (9.1)
Two	3 (8.3)	3 (9.1)	3 (8.3)	2 (6.1)	1 (2.8)	1 (3.0)

Dombrowski SU, *et al. BMJ Open* 2020; 10:e032653. doi: 10.1136/bmjopen-2019-032653

Three	3 (8.3)	2 (6.1)	2 (5.6)	0 (0.0)	1 (2.8)	0 (0.0)
Four	1 (2.8)	0 (0.0)	1 (2.8)	0 (0.0)	0 (0.0)	0 (0.0)
**Number of entries on self-monitoring feature, count**						
Weight	69	115	45	23	42	46
Steps	419	296	350	102	397	74
Waistline	4	7	5	0	2	0
Belt notches	8	0	4	0	0	0

Note. M = mean, SD = Standard Deviation.

Dombrowski SU, *et al*. *BMJ Open* 2020; 10:e032653. doi: 10.1136/bmjopen-2019-032653

Online supplement 3 Satisfaction and helpfulness ratings for overall programme and narrative texts at 12 months by deprivation status

	SMS+I		SMS only		Total	
	SIMD 1, 2 (n=14)	SIMD 3+ (n=7)	SIMD 1, 2 (n=16)	SIMD 3+ (n=10)	SIMD 1, 2 (n=30)	SIMD 3+ (n=17)
Satisfaction programme[a]	81.8, 21.7	79.9, 19.2	74.6, 23.5	81.0, 16.0	77.9, 22.6	80.5, 16.8
Helpfulness programme[b]	4.2, 1.1	3.9, 1.1	3.9, 1.1	4.2, 1.0	4.1, 1.1	4.1, 1.0
Helpfulness of narrative texts[b]	3.4, 1.3	3.4, 1.4	3.0, 1.5	3.8, 1.2	3.2, 1.4	3.6, 1.3

Note. All data mean (SD), [a] scored 0-100 (higher = more satisfied), [b] scored 1-5 **(**1 = low, 5 = high**),** SIMD = Scottish index of multiple deprivation 1 represents the most disadvantaged area; quintile SIMD 5 represents the least disadvantaged area SMS = Short message service, SMS+I = SMS plus incentive group.

Dombrowski SU, *et al. BMJ Open* 2020; 10:e032653. doi: 10.1136/bmjopen-2019-032653
