# Supplementary material for: Game of Stones: feasibility randomised controlled trial of how to engage men with obesity in text message and incentive interventions for weight loss
Source: BMJ Open. 2020 Feb 25;10(2):e032653. doi: 10.1136/bmjopen-2019-032653 (PMC7045214; doi:10.1136/bmjopen-2019-032653)
Supplement: Author's manuscript [file bmjopen-2019-032653.draft_revisions.pdf]

BMJ Open is committed to open peer review. As part of this commitment we make the peer review history of every article we publish publicly available.

When an article is published we post the peer reviewers' comments and the authors' responses online. We also post the versions of the paper that were used during peer review. These are the versions that the peer review comments apply to.

The versions of the paper that follow are the versions that were submitted during the peer review process. They are not the versions of record or the final published versions. They should not be cited or distributed as the published version of this manuscript.

BMJ Open is an open access journal and the full, final, typeset and author-corrected version of record of the manuscript is available on our site with no access controls, subscription charges or pay-per-view fees (http://bmjopen.bmj.com).

If you have any questions on BMJ Open's open peer review process please email info.bmjopen@bmj.com

BMJ Open

Game of Stones: Feasibility randomised controlled trial of how to engage men with obesity in text message and incentive interventions for weight loss

Journal:	*BMJ Open*
Manuscript ID	bmjopen-2019-032653
Article Type:	Original research
Date Submitted by the Author:	02-Jul-2019
Complete List of Authors:	Dombrowski, Stephan U; University of New Brunswick Fredericton, Kinesiology<br>McDonald, Matthew; University of Stirling<br>van der Pol, Marjon; University of Aberdeen, Health Economics Research Unit<br>Grindle, Mark; University of the Highlands and Islands<br>Avenell, Alison; University of Aberdeen<br>Carroll, Paula; Mens Health Forum in Ireland<br>Calveley, Eileen; University of Stirling<br>Elders, A; Glasgow Caledonian University School of Health and Life Sciences, NMAHP Research Unit ; Glasgow Caledonian University<br>Glennie, Nicola ; University of Stirling<br>Gray, Cindy; University of Glasgow, Institute of Health and Wellbeing<br>Harris, Fiona; University of Stirling,<br>Hapca, Adrian; University of Dundee, Population Health Sciences<br>Jones, Claire; University of Dundee<br>Kee, Frank; Queen's University Belfast, UKCRC Centre of Excellence for Public Health (NI)<br>McKinley, Michelle C.; Queen's University Belfast<br>Skinner, Rebecca; University of Stirling<br>Tod, Martin; Mens Health Forum<br>Hoddinott, Pat; University of Stirling, Nursing, Midwifery and Allied Health Professional Research Unit
Keywords:	obesity, men, feasibility, trial, SMS, weight loss

SCHOLARONE™
Manuscripts

I, the Submitting Author has the right to grant and does grant on behalf of all authors of the Work (as defined in the below author licence), an exclusive licence and/or a non-exclusive licence for contributions from authors who are: i) UK Crown employees; ii) where BMJ has agreed a CC-BY licence shall apply, and/or iii) in accordance with the terms applicable for US Federal Government officers or employees acting as part of their official duties; on a worldwide, perpetual, irrevocable, royalty-free basis to BMJ Publishing Group Ltd ("BMJ") its licensees and where the relevant Journal is co-owned by BMJ to the co-owners of the Journal, to publish the Work in this journal and any other BMJ products and to exploit all rights, as set out in our licence.

The Submitting Author accepts and understands that any supply made under these terms is made by BMJ to the Submitting Author unless you are acting as an employee on behalf of your employer or a postgraduate student of an affiliated institution which is paying any applicable article publishing charge ("APC") for Open Access articles. Where the Submitting Author wishes to make the Work available on an Open Access basis (and intends to pay the relevant APC), the terms of reuse of such Open Access shall be governed by a Creative Commons licence – details of these licences and which Creative Commons licence will apply to this Work are set out in our licence referred to above.

Other than as permitted in any relevant BMJ Author's Self Archiving Policies, I confirm this Work has not been accepted for publication elsewhere, is not being considered for publication elsewhere and does not duplicate material already published. I confirm all authors consent to publication of this Work and authorise the granting of this licence.

For peer review only

Game of Stones: Feasibility randomised controlled trial of how to engage men with obesity in text message and incentive interventions for weight loss

Stephan U Dombrowski[1, 2], Matthew McDonald[3], Marjon van der Pol[4], Mark Grindle[5], Alison Avenell[6], Paula Carroll[7], Eileen Calveley[3], Andrew Elders[8], Nicola Glennie[3], Cindy M Gray[9], Fiona M Harris[3], Adrian Hapca[10], Claire Jones[11], Frank Kee[12], Michelle C McKinley[13], Rebecca Skinner[2], Martin Tod[13], Pat Hoddinott[3]

Corresponding author:

Stephan U Dombrowski

Assistant Professor

Faculty of Kinesiology

90 Mackay Drive

Fredericton, New Brunswick,

Canada, E3B 5A3,

Email: stephan.dombrowski@unb.ca

Tel: 1 506 453 4803

[1]Faculty of Kinesiology, University of New Brunswick

[2]Division of Psychology, University of Stirling

[3]Nursing, Midwifery and Allied Health Professions Research Unit, University of Stirling

[4]Health Economics Research Unit, University of Aberdeen

[5]Institute for Health Research and Innovation, University of the Highlands and Islands

[6]Health Services Research Unit, University of Aberdeen

[7]Men's Health Forum in Ireland

[8]Nursing, Midwifery and Allied Health Professions Research Unit, Glasgow Caledonian University

For peer review only

[9]Institute of Health & Wellbeing, University of Glasgow

[10]Tayside Clinical Trials Unit, University of Dundee

[11]Health Informatics Centre, University of Dundee

[12] Centre for Public Health, Queen's University Belfast

[13]The Men's Health Forum (GB), London

Word count: 5624

Key words: obesity, men, feasibility, trial, SMS, weight loss, behaviour change, financial incentives

For peer review only

For peer review only - http://bmjopen.bmj.com/site/about/guidelines.xhtml

ABSTRACT

Objectives

To examine the acceptability and feasibility of narrative text messages with or without financial incentives to support weight loss for men.

Design

Individually randomised three-arm feasibility trial with 12 months' follow-up.

Setting

Two sites in Scotland with high levels of disadvantage according to Scottish Index for Multiple Deprivation (SIMD).

Participants

Men with obesity (n=105) recruited through community outreach and general practitioner registers.

Interventions

Participants randomised to a) narrative text messages plus financial incentive for 12 months (SMS+I), b) narrative text messages for 12 months (SMS only), or c) waiting list control.

Outcomes

Acceptability and feasibility of recruitment, retention, intervention components and trial procedures assessed by analysing quantitative and qualitative data at three, six and 12 months.

Results

105 men were recruited, 60% from more disadvantaged areas (SIMD quintiles 1 or 2). Retention at 12 months was 74%. Fewer SMS+I participants group (64%) completed 12-month assessments compared to SMS only (79%) and control (83%). Narrative texts were acceptable to many men, but some reported negative reactions. No evidence emerged that level of disadvantage was related to acceptability of narrative texts. Eleven SMS+I participants (31%) successfully met or partially met weight loss targets. The cost of the incentive per participant was £81.94 (95% CI £34.59-£129.30). Incentives were acceptable, but improving health was reported as the key motivator for weight loss. All groups lost

For peer review only

weight (SMS+I -2.51kg (SD=4.94); SMS only -1.29kg (SD=5.03); control -0.86kg (SD=5.64) at 12 months).

Conclusions

This weight management intervention recruited and retained men from across the socioeconomic spectrum with the majority from areas of disadvantage, was broadly acceptable to most participants and indicated some weight loss benefits.

Trial registration

ClinicalTrials.gov Identifier: NCT03040518

Funding details

Funded by the National Institute for Health Research Public Health Research Programme (14/185).

For peer review only

For peer review only - http://bmjopen.bmj.com/site/about/guidelines.xhtml

STRENGTHS AND LIMITATIONS OF THIS STUDY

- This weight management study for men with obesity recruited men across the socio-economic spectrum, with a specific focus on more disadvantaged areas, to a randomised controlled feasibility study examining SMS with or without endowment incentives.
- Acceptability and feasibility was established using a multi-lens perspective drawing on quantitative, qualitative and trial procedure data.
- Effectiveness of intervention components will need to be established in a full multi-centre trial.

For peer review only

For peer review only - http://bmjopen.bmj.com/site/about/guidelines.xhtml

BACKGROUND

Obesity is associated with an increased risk of serious health conditions such as type 2 diabetes, cardiovascular disease and some cancers.[1] In 2016, 26% of men in the UK were classified as obese, and men were less likely to be a healthy weight than women (31% of men compared with 35% of women).[2 3]

Despite the growing prevalence of obesity, men contribute a disproportionately low number of participants to evidence-based weight management programmes.[4] Most interventions are designed for mixed sex populations, but systematic review evidence suggests that, compared to women, men often benefit from different ways of providing interventions.[4]

Text message interventions

The evidence for text message interventions supporting changes in lifestyle behaviours, including behaviour change for weight loss, is promising.[5-7] However, systematic reviews[4-7] report no text message delivered trials that target weight loss designed for men only. Text-based interventions can reach large numbers of people, including men from disadvantaged backgrounds,[8] and mobile technologies such as standard mobile phones allow delivery of evidence-based strategies anywhere and anytime.

Narrative approaches to promoting behaviour change have been suggested as a tool for communication-based interventions.[9] Narrative SMS can be broadly defined as interactive life stories, which are based around a group of characters with whom recipients can identify.[10] Text message based interventions using narratives have been used to engage hard to reach men in moderating their alcohol consumption, and were found to be acceptable.[11-13] Narrative approaches to weight management in men may be a promising tool to support behaviour and weight change.

Financial incentive interventions

Systematic review evidence of financial incentives for behaviour change highlights the potential for incentives to change behaviours and help reduce health inequalities.[14] A discrete choice experiment found that paying people (£10-£30/week varying by age and weight) to take part in diet and physical activity (PA) interventions is likely to improve uptake, adherence and maintenance of behaviour change.[15] Moreover, the evidence for financial incentives for weight loss is growing.[16-19] In particular, deposit contracts, where participants deposit their own money and are reimbursed only if they achieve the target weight loss, are effective whilst the incentives are in place.[4 20] Deposit contracts draw on loss aversion where

For peer review only - http://bmjopen.bmj.com/site/about/guidelines.xhtml

people are more motivated to avoid losses than they are to achieve similarly sized gains.[21 22] However, deposit contracts may not be not equitable, as committing one's own money up front may not be possible, particularly for individuals with lower income.

Endowment incentive contracts may overcome limitations of deposit contracts. Endowment incentive contracts are financial incentives where a person is endowed with a (hypothetical) amount of money which they either 'secure' or 'lose' depending on achieving certain targets. A trial that framed financial incentives in this way found these incentives were effective for achieving PA goals in the short term.[23]

For peer review only

Aim

The aim of the Game of Stones feasibility trial was to examine the acceptability and feasibility of a men-only weight management intervention consisting of narrative text messages, with and without an endowment incentive, compared to waiting list control. The objectives were to:

1. Assess the acceptability and willingness to be randomised to: i) narrative text message and endowment incentives; ii) narrative text messages only; or iii) waiting list for text messages (control).
2. Assess the feasibility of recruiting from general practitioner (GP) practice obesity registers and community venues.
3. Determine the acceptability of intervention content, feasibility of delivery, fidelity and any unintended consequences.
4. Assess indicative effects on weight change and progression criteria for a full trial.

METHODS

Trial design

A three-arm individually randomised parallel-group controlled feasibility trial was conducted. Participants were randomised to receive narrative text messages and endowment incentives (SMS+I), narrative text messages only (SMS only), or a waiting list for text messages (Control). The protocol and the a priori progression criteria for the full trial are available: https://www.journalslibrary.nihr.ac.uk/programmes/phr/1418509/#/

Ethical approval for the study was obtained from the North of Scotland Research Ethics Service (Ref: 16/NS/0120).

For peer review only - http://bmjopen.bmj.com/site/about/guidelines.xhtml

Setting

Two Health Board areas in Scotland (Sites A and B) with high levels of disadvantage.

Eligibility criteria

Participants met the following eligibility criteria: men over 18 years old; BMI ≥30kg/m^2 and/or a waist circumference of ≥40 inches (102 cm); owned a mobile phone capable of receiving text messages; could understand English language text messages; not already taking part in a weight loss study; not planning or waiting to have bariatric surgery; not planning to move within the next 12 months; considered by practice clinical staff as suitable for participation (GP practice recruitment only), for example, no severe medical, terminal or psychiatric illness (in patient or close family member) or no significantly impaired cognitive function.

Sample size

This study aimed to randomise 105 men, 35 to each arm, in line with recent recommendations for pilot trials as sufficient to estimate key parameters for a full trial.[24] A sample size of 35 per arm is sufficient to allow the population variance to be estimated (e.g. the SD in weight loss) with enough precision to deliver at least 80% power and 90% confidence in a full trial, with a standardised effect size between 0.2 and 0.5.[24]

Participant recruitment

Participants were recruited via: 1) community outreach, and 2) GP practice obesity registers.

Community outreach

Community recruitment strategies that report success for recruiting men in disadvantaged areas were used.[11-13] This included researchers working on stands with study information leaflets and table banners in supermarkets, fitness centres, hospital foyers, health centres, council workplaces and community centres across both sites. Eleven of the 13 recruitment venues were within more disadvantaged areas based on the Scottish Index of Multiple Deprivation (SIMD quintiles 1 and 2).[25] Men who showed interest were given study information and asked to leave their contact details with researchers.

In addition, some men were recruited through word of mouth. This included researcher discussions with family, friends, colleagues and local workers who passed on study information to eligible men they knew. Information leaflets were distributed across localities

For peer review only - http://bmjopen.bmj.com/site/about/guidelines.xhtml

in shops, libraries, barbers and community centres. Interested men then contacted researchers to get more information.

GP practice obesity register letters

Practices within more disadvantaged areas (SIMD quintiles 1 or 2) were invited to participate in the study. Of the 33 practices invited (n= 13 in Site A, n=20 in Site B), five practices participated (n=4 in Site A, n=1 in Site B).

Practice database searches identified potentially eligible men with a documented BMI of at least 30kg/m^2. Lists were screened by clinical practice staff and details passed to the Health Informatics Centre at the University of Dundee who sent out GP-headed study invitation letters and information leaflets. Interested men contacted the research team or returned an 'opt-in' card.

Baseline Appointment

Men interested in participating were invited to attend a face-to-face appointment. Detailed study information was discussed and written informed consent provided. Anthropometric measurements were conducted and eligibility assessed. Participants then completed a baseline questionnaire, and were randomised. Group-specific post-randomisation information was provided and discussed with the participant.

Randomisation

At the baseline appointment, independent randomisation was performed by the researcher using the clinical trials unit's secure remote web-based system, stratifying by recruitment method (GP and community) and recruitment site (Site A and Site B).

Blinding

The two recruitment researchers who also assessed outcomes and conducted qualitative interviews were mostly not blinded to group allocations. All other study team members were blinded. A demonstration of the feasibility for researcher outcome assessors to be blind to group allocation was conducted with eleven participants at the six month assessment. In this case, a researcher who had not previously met the participant arranged and undertook the assessment. These participants all complied with the request not to reveal their group allocation.

For peer review only - http://bmjopen.bmj.com/site/about/guidelines.xhtml

The trial statistician was fully blinded to intervention groups, and partially blinded for the control group due to the different response schedule (control participants were only assessed at baseline months and 12 months to reflect "usual life").

Intervention components

Narrative text messages

A narrative text message library consisting of 604 texts was written by a professional scriptwriter/researcher (MG). Full details of the narrative texts development process are available elsewhere.[10] Narrative texts were sent to participants over the course of 12 months and were written from the point of view of a fictional character aiming to lose weight over 12 months. Narrative texts were written to appeal to men from disadvantaged backgrounds. All participants in the SMS+I and SMS only groups received automated texts according to a pre-determined schedule. Participants could reply to texts but did not receive a response. Texts were sent between 8:00AM and 10:00PM and ranged from 0-5 texts per day. Some texts were personalised including participants' names and men could select whether weight information should be presented in kilogrammes or stones and pounds. No further personalisation and tailoring options were offered. Texts were delivered by the Health Information Centre in Dundee using existing automated technology linked to a clinical trials unit database.

Endowment incentive

The incentive strategy was informed by existing evidence and men's preferences elicited using a Discrete Choice Experiment completed by 1,045 men with obesity and reported elsewhere.[10] SMS+I participants were 'endowed' with a £400 incentive at baseline which was placed into a hypothetical personal account at the University of Stirling and given a mock-up personalised cheque. The full £400 could be secured by meeting weight loss targets at researcher assessments: 5% of body weight lost since baseline at three months (£50 secured/lost), 10% lost since baseline at six months (£150 secured/lost) and 10% lost since baseline at 12 months (£200 secured/lost) (online supplement 1). Weight loss was verified at all face-to-face appointments. At six and 12 months, men lost a proportion of the money for each % weight loss not attained between 5-10%. Weight at 12 months had to be less than at baseline to receive any money, regardless of whether interim weight loss targets had been met. Men received the money by direct bank transfer after the 12-month assessment.

For peer review only - http://bmjopen.bmj.com/site/about/guidelines.xhtml

Feedback on meeting incentive targets was sent by automated text message and displayed on the personalised SMS+I webpage.

Website

All trial participants were provided with a unique login ID for the Game of Stones website. The front page was accessible to all participants and included trial information and links to existing evidence-based online weight management resources. SMS +I and SMS only webpages had a brief biography and images of the fictional characters featured in narrative texts. Participants could enter their weight, pedometer steps, waist circumference and belt notches, which were displayed as basic visual progress charts. Only individual performance was displayed on the webpage, and no group averages were shown for social comparison. SMS+I webpages described the financial incentives and a visual progress chart of money secured/lost.

Printed information

All participants received a weight loss fact sheet (British Dietetic Association, Weight Loss Food Fact Sheet) and a small card which could be carried in their wallet for noting website details and their appointment weight, weight loss targets and appointments.

Pedometer

All participants received a study pedometer (3DFitBud, A420S, manufactured by 3DActive).

Comparator group

At baseline, participants received access to the information section of the webpage, printed information (i.e. a weight loss fact sheet) and a pedometer. They attended a baseline and 12-month appointment only. Control group participants were offered texts for three months commencing after the 12 month data collection point.

Outcome assessment

Outcomes were assessed at baseline, three, six and 12 months for intervention participants (SMS+I and SMS only), and at baseline and 12 months for control participants. Individual appointments were at community centres, universities, NHS clinical research facilities, voluntary sector organisations, GP practice premises, or the participant's home if no suitable alternative venue could be found. At the 12 month appointment, all participants received a £20 voucher as reimbursement for their time. No travel expenses were provided.

For peer review only - http://bmjopen.bmj.com/site/about/guidelines.xhtml

The text message delivery system automatically the frequency of responses received to the texts. The website automatically recorded engagement with self-monitoring tools.

Self-report questionnaires were completed during appointments and measured socio-demographics, co-morbidities, disability, ethnicity, and perceptions on intervention acceptability. Overall satisfaction with the intervention was assessed with the item: "*On a scale from 0 (not satisfied at all) to 100 (completely satisfied). How satisfied are you with the Game of Stones programme?*". Acceptability of the intervention was assessed with the stem "*Overall the Game of Stones programme has been…*" followed by options '*understandable*', '*useful*', '*helpful*', '*interesting*' and '*relevant*', with responses captured on a 5-point scale ranging from '*totally disagree*' to '*totally agree*'. Helpfulness of the intervention was assessed with the item "*How helpful have you found the following in helping you lose weight?*" followed by options '*text messages*', '*website*', and '*pedometer*', with responses captured on a 5-point scale ranging from '*totally unhelpful*' to '*totally helpful*'. Measures of overall satisfaction, acceptability and helpfulness were adapted from Dombrowski et al., (2012).[26]

Anthropometry measures included height measured at baseline using a portable standing stadiometer (Seca 217, Birmingham, UK) to the nearest 0.1cm. Prior to weight measurements, participants removed shoes and bulky clothing and items from their pockets. Weight was recorded using portable calibrated electronic scales (Marsden M420, Rotherham, UK) to the nearest 0.01kg and waist circumference using a tape measure (Seca 203, Birmingham, UK) to the nearest 0.1cm.

Information on possible adverse events was recorded at assessment visits via open questions and through automatic monitoring of text message replies including words like 'suicide', 'die', and 'death' indicative of potential adverse events, which were notified to the research team by email.

Interviews took place during three and 12 month appointments and were conducted face-to-face and detailed methods are described elsewhere.[10] All audio recorded interviews were transcribed verbatim and anonymised. At three months, all SMS+I and SMS only men were invited to participate in either a brief feedback or an in-depth interview (participant choice). The three month topic guide focussed on the early acceptability of the intervention components in order to identify any refinements required over the remaining nine month intervention. For the 12 month qualitative interviews, separate topic guides for the three trial

For peer review only - http://bmjopen.bmj.com/site/about/guidelines.xhtml

groups were informed by the analysis of the three-month interviews, researcher field notes and to gain information power[27] to address the study objectives. Purposive sampling from the three trial groups was informed by the three month interview data and researcher field notes to provide diversity of perspectives. Researcher field notes taken at all assessments were referred to when interpreting interview data.

Qualitative interviews were conducted face-to-face with 50 of 58 men who attended the three month assessment (7-61 minutes, median=23 minutes). At 12 months, interviews were conducted with 14 participants from SMS+I (13-65 minutes, median=33 minutes), 13 from SMS only (10-59 minutes, median=28 minutes) and six from the control group (7-18 minutes, median=11 minutes). Fourteen interviews at 12 months lasted <20 minutes, mostly for control participants who had received no contact from the research team between baseline and 12 months.

Analysis

Quantitative analysis

All continuous variables were summarised and tabulated using the following descriptive statistics: N (number of valid non-missing responses), mean, standard deviation (SD). Likert-scale variables were treated as continuous measures. The frequency and percentages (based on the non-missing sample size) of observed levels are reported for all categorical measures. The proportion of individuals contacted who were recruited and the proportions retained and withdrawn at each assessment by group was determined. Missing weight data are presented as baseline observation carried forward (BOCF) and last observation carried forward (LOCF) in addition to observed cases only.

CONSORT guidance for reporting randomised pilot and feasibility studies was followed[28].

Qualitative and mixed method data analysis

Anonymised transcripts of interviews together with: attributes for recruitment channel; attendance at assessments, trial group and participant characteristics and weight loss outcomes were entered into NVivo12 software for analysis guided by the framework approach.[29] Reference to researcher field notes contributed to interview data interpretation. Iterative data collection and analysis were driven by the key feasibility and acceptability research questions and objectives. A coding frame was developed by three researchers independently reading a diverse sample of six interviews, followed by a team discussion to finalise the coding frame and identify key themes. Independent coding was conducted by four

For peer review only - http://bmjopen.bmj.com/site/about/guidelines.xhtml

researchers (EC, NG, MM and RS) and checked for consistency by FH. Two independent researchers (EC and NG) who were not involved in any other aspect of the study, assisted with coding and analysis to enhance rigour and reliability of data analysis. Emergent themes and interpretive analysis were discussed at weekly researcher meetings and at two Co-Investigator qualitative interpretation meetings.

At the final stage of mixed methods data interpretation, the quantitative attributes on NVivo were drawn on to triangulate the analysis and suggest further avenues for interrogation. Matrix coding queries in NVivo were generated to cross-reference attributes for trial group, participant SIMD and weight loss outcome at 12 months, with nodes coded for views of incentives and texts messages respectively. Credibility and reliability of qualitative analysis was enhanced by independent coding, use of memos to ensure transparency of interpretation, and interpretive charting conducted with input from the wider study team.

Patient and Public Involvement

A continuous and responsive approach to patient and public involvement (PPI) was adopted to prepare the grant application and throughout the study, as described by Gamble and colleagues.[30]

Continuous PPI was provided by a Co-Investigator partnership with the Charities Men's Health Forum GB and Men's Health Forum in Ireland. The partnership commenced in 2011 with the ROMEO evidence syntheses of weight loss interventions for men with obesity.[4]

During the study, Co-Investigators from each of the Men's Health Forum charities attended trial management meetings to contribute to decisions, intervention development, data analysis, interpretation of findings and reporting. They provided feedback on the grant application, protocol, text messages, information materials and engaged wider involvement of men from their organisation to assist with appropriate language.[31]

Continuous PPI at the study oversight level was provided by two independent lay members of the study steering committee which met on three occasions.

Responsive PPI occurred at the study funding application stage. Co-Investigator CG engaged men who provided PPI input in the FFIT Trial.[32] During the study, relevant members of the public were identified through several sources including: Men's Health Forum GB, Men's Health Forum in Ireland, Scottish Community Health Councils, Men's Sheds, University of Stirling PPI group, Alliance Scotland and other Co-Investigator contacts. Effort was made to engage men from more disadvantaged communities. A total of 121 PPI contributors were

For peer review only - http://bmjopen.bmj.com/site/about/guidelines.xhtml

involved in a range of study activities, including finding a study name, helping with wording of information material, and helping with designing the intervention content. For full details see the report to the funder.[10]

RESULTS

Recruitment and retention

Recruitment was completed in four months between March to June 2017 meeting the target number of 105 men randomised (Figure 1).

Overall, 177 men expressed an interest in the study. Baseline appointments were attended by 111 (63%), of which six (5%) were ineligible. All 105 men consented to be randomised to the SMS+I (n=36), SMS only (n=33) and control (n=36) groups.

GP practice recruitment was undertaken in five GP practices (Site A n=4, Site B n=1) with 45 men recruited and randomised through GP practices. The additional 60 men were recruited through community outreach.

--- Figure 1 ---

The 12 month assessment was completed by 79/105 participants (75%). One of the 79 participants (control group) could not attend and provided self-reported information because he was out of the region due to work commitments, which means that 74% (78/105) of participants were retained for weight assessments at 12 months. Overall 12 month retention differed by group (SMS+I=23/36, 64%; SMS only=26/33, 79%; control=30/36, 83%). Fourteen participants (13%) were lost to follow-up, and 12 participants (11%) withdrew. Reasons for withdrawal were dislike of narrative texts (n=4), health (n=3), family (n=1), unknown (n=1), dissatisfaction with group allocation (n=1), appointment logistics (n=1) and multiple reasons (n=1).

Baseline characteristics

Baseline characteristics are displayed in Table 1. On average, participants were 52.2 (SD=13.1) years old, had a BMI of 35.7 (SD=5.9) and a waist circumference of 116.8 cm (SD=11.8). BMI ranged from 27.5 kg/m^2 to 62.5kg/m^2. Twelve participants (11%) had a baseline BMI less than 30 kg/m^2 due to the additional weight circumference entry criterion of ≥40 inches (102 cm).

The majority of men lived in disadvantaged areas defined as SIMD quintiles 1 or 2 (n=62/104; 60%). Most participants were married (58%), reported at least one co-morbidity

For peer review only - http://bmjopen.bmj.com/site/about/guidelines.xhtml

(63%), were of white ethnicity (91%), reported having children (76%), and were in full-time employment (48%).

--- Table 1 ---

Intervention fidelity

The intervention components were feasible to deliver with fidelity. The majority (95.4%) of the 38,214 text messages sent to the two intervention groups were delivered to the mobile phone, with 1,782 (4.6%) having no delivery status (i.e. lack of response from the mobile phone provider after 48 hrs and invalid phone numbers). No major technical errors occurred. All participants who secured financial incentives were paid by direct bank transfer, in line with their stated preference.

Acceptability of intervention components

Overall acceptability ratings and contamination

Table 2 displays intervention satisfaction indicators over the course of the study for participants attending assessments. Overall mean study satisfaction at 12 months was 81%, 77% and 87% for the SMS+I, SMS only and control groups respectively. Satisfaction over time was comparable between both intervention groups. For scores on specific programme attributes see Table 2.

Helpfulness ratings of the narrative texts, webpage and pedometer were highest for the pedometer and relatively stable throughout the measurement time points. At 12 months, helpfulness ratings (out of 5) for the pedometer were 3.9, 4.0, and 3.7 for the SMS+I, SMS only and control groups respectively. The narrative texts (SMS+I=3.4; SMS only=3.3) and the webpage (SMS+I=3.6; SMS only=3.4; control=3.5) were perceived as somewhat helpful on average at 12 months.

Minimal contamination between intervention groups was observed, with one participant at six and 12 months (SMS+I group) and another at 12 months (control group) reporting meeting other men in the study.

--- Table 2 ---

Acceptability of narrative text messages

Some men sent spontaneous replies to the narrative texts (0-3 months n=25/69, 36%; 3-6 months n=8/69, 12%, 6-12 months n=13/69, 19%; see online supplement 2). Most

For peer review only - http://bmjopen.bmj.com/site/about/guidelines.xhtml

spontaneous replies were received between 0-3 months (n=370 replies) and decreased at 3-6 months (n=16 replies) and 6-12 months (n=39 replies).

Eleven participants (11/69, 16%) requested to no longer receive texts but asked to remain in the trial. Requests to stop narrative texts were similar in both groups (SMS+I=5/36, 14%; SMS only=6/33, 18%), and occurred throughout the study. Four participants (4/69, 6%) withdrew due to dislike of narrative texts between baseline and three months, two in each intervention group.

Qualitative interviews at three and 12 months demonstrated varied views on the narrative texts. Participants' views ranged from positive to indifferent to negative. Those who liked the narrative texts found the storyline entertaining, engaging and some participants felt a certain camaraderie for the main character:

> *You get involved in the content and you start following the script, they're very funny and me being a [from city name] you can see the funny side and you can see the wit in that, …. and that's what keeps you reading them.* (210010, SMS only, 12m)

For other participants, the frequency of texts became a source of irritation, the storyline did not resonate with their own experience nor could they empathise with the fictional characters:

> *I'm not a lover of chocolate, biscuits, cake, ice cream, I hardly ever touch them, hardly ever, so hearing about somebody eating pizza or sweets means nothing to me because I don't eat them anyway* (120002, SMS+I, 12m)

Some were uncertain of the role of the texts in helping to support weight loss. Others expressed an indifference towards the texts, and a decreasing interest in the story over time leading to infrequent engagement with text content. However, the regular reminder of being a participant in a weight loss programme through receiving texts was seen as important:

> *I often wonder, do they relate to myself here. And the only commonality is both trying to lose weight. So in that respect it's just a reminder all the time, you should be reviewing your weight and watching what you're eating, so…from that it's a positive, yeah.* (220017, SMS+I, 3m)

A matrix coding query and analysis of the quotations generated from the NVivo qualitative and demographic data suggested no obvious relationships between the participant SIMD level and the qualitative accounts of acceptability of the narrative texts. This is in line with quantitative data showing similar acceptability ratings for overall programme satisfaction and

For peer review only - http://bmjopen.bmj.com/site/about/guidelines.xhtml

helpfulness ratings of the narrative texts across SIMD levels (see online supplement 3). Some men would have liked more interactivity built into the texts, however, the text message system was set up for uni-directional messaging only.

Acceptability of endowment incentive

At three months, 31/36 SMS+I participants attended the appointment, with eight men losing 5% or more of their baseline weight and securing £50. Attendance at six months was lower (n=23/36), with five participants achieving the weight loss target of >10% weight loss securing £150 each, and a further three participants losing between ≥5-10% of weight securing between £75-150. At 12 months, 23/36 participants attended the appointment, with three achieving >10% weight loss securing £200, and a further seven losing between ≥5-10% of weight securing between £100-200.

Overall, £2,955 was paid in total to the 11 participants (11/36, 31%) successfully meeting or partially meeting weight loss targets. The full £400 was secured by three participants (3/36, 8%). The cost of the incentive per participant was £81.94 (95% CI £34.59-£129.30). No participant secured money at three or six months which was then lost due to weight regain to baseline weight at 12 months.

No negative views were expressed about the incentives in qualitative interviews. Only one man reported being motivated by the financial incentive, and many expressed indifference. Participants reported the weight loss and health benefits as sufficiently rewarding.

> *The money's not that much of an issue. I think the incentive for me is the health that, at the end of the day, your health improves. That's the most important incentive. The money, it's fine, it's there, but it's not the incentive* (120022, SMS+I, 3m)

Some interpreted the incentives as a final reward linked to their weight loss targets, whereas others fully understood the intended use of the loss aversion concept:

> [W]e *all know how much more distress it causes you to lose money than the pleasure of finding a fiver down the back of the couch, you know, so I think if you lose something it almost has a bigger impact on you than if you gain*. (220040, SMS+I, 3m)

For peer review only - http://bmjopen.bmj.com/site/about/guidelines.xhtml

Acceptability of study webpages and pedometer

Some participants used the self-monitoring features on the webpage (see online supplement 3) and reported valuing the ability to visually track their weight in interviews. However, most did not access the website. The majority (77%) did not own a pedometer at baseline, and pedometers were highly acceptable to participants.

Acceptability of Control Group

Most control group participants (30/26, 83%) attended the 12 month appointment and many reported being pleased to be involved in a research study. One participant had a strong negative reaction to being randomised to the control group and subsequently withdrew when invited to the 12 month assessment.

Weight outcomes

Table 3 displays weight change outcomes at three, six, and 12 months. The difference in mean percentage weight loss at 12 months for observed cases was −2.51% (95% CI −6.03 to 1.01) for SMS+I v control and −0.51% (95% CI −3.91 to 2.89) for SMS only v control. The difference in absolute weight loss at 12 months was −2.87kg (95% CI −6.82 to 1.08) for SMS+I v control and −0.57kg (95% CI −4.40 to 3.25) for SMS only v control.

---Table 3 ---

The SMS+I group displayed mean weight loss of over 3% at 12 months (-3.51%, SD=5.83). The SMS only and control groups remained below 3% weight loss on average (SMS only=-1.51%, 4.65; control=-1.00%, SD=5.31). The highest mean percentage weight loss at 12 months for BOCF was -2.24% (SD=4.93) in the SMS+I group, followed by -1.19% (SD=4.16) and -0.80% (SD=4.77) in the SMS only and control groups respectively.

Harms and unintended consequences

No harms or unintended consequences were reported.

DISCUSSION

Principal findings

This study successfully recruited 105 men from across the socioeconomic spectrum to a three-armed RCT and overall achieved 74% retention at 12 months' follow-up. Men living in more disadvantaged areas formed 60% of the sample. Narrative texts were broadly acceptable, but some participants disengaged or withdrew from the study due to dislike of

For peer review only - http://bmjopen.bmj.com/site/about/guidelines.xhtml

texts. The endowment incentives were acceptable, and improving health was reported as a key motivator for weight loss. Positive indicative effects of weight loss were found in intervention and control groups.

Strengths and weaknesses of the study

This study was underpinned by the ROMEO systematic reviews and qualitative evidence synthesis of weight loss interventions for men with obesity[33 34], with a continuing PPI partnership with the Men's Health Forum GB and Ireland charities. Mixed methods research combined quantitative and qualitative data which, together with patient, public and stakeholder involvement, provided a multi-lens perspective on this study.

Most assessments were not blind to group allocation. Researchers were unable to collect data via qualitative interviews, questionnaires and anthropometric measures with men who withdrew from the study or were lost to follow-up at the 12 month appointment. Those providing a withdrawal reason may have provided socially desirable responses. Text messages were personalised by including the participant's name and weight unit preference, but no tailoring or interactivity were possible due to technical limitations of the delivery system. This study was not powered to detect effects on weight loss; study and weight outcomes should be interpreted with caution.

Relation to other studies

The Game of Stones study adds to the evidence base demonstrating the feasibility of recruiting men for research on sensitive subjects, such as obesity, through community outreach and GP practice lists.[11-13]

The average BMI and age of the study participants of around 35kg/m^2 and 50-55 years is similar to UK weight management trials recruiting in the community[32], primary care[35], or a combination of community and primary care.[12] Three international text message based weight management studies examining outcomes after 12 months recruited younger participants with lower BMI.[36-38] However, three mixed sex weight management studies with financial incentives (two including text message components) reported broadly similar participant demographics to this study.[39-41]

Retention levels of 74% were acceptable and are similar to systematic review evidence of men-only weight loss interventions which found an average retention of 78%, ranging from 44% to 100%.[34] International text message studies with mixed sex participants report similar retention rates at 12 months of 70.3%[37] and 73%[36], with one study conducted in Latvia

For peer review only - http://bmjopen.bmj.com/site/about/guidelines.xhtml

reporting 93% retention[38]. Two large UK studies recruiting mixed sex participants with obesity to weight management in primary care reported 75%[35] and 81%[42] retention at 12 months.

Two previous narrative text message intervention studies in men targeting alcohol reduction reported high acceptability levels and no negative reactions.[12 13] However, these studies were only 12 weeks in duration and overall contained fewer texts. The narrative texts in this study were broadly acceptable, although some negative reactions were reported. Study withdrawal due to texts and requests to stop texts suggest that the current narrative texts may not be universally acceptable to men. The text messages were designed by a professional scriptwriter/researcher with PPI input from the target population of men from disadvantaged backgrounds, but the narrative texts were similarly acceptable across the socio-economic spectrum. Intervention fidelity was high with the majority of sent texts successfully delivered and no adverse events were encountered, similar to previous text message-based intervention studies.[11-13]

The use of a financial incentive strategy for weight loss in men with obesity across the socioeconomic spectrum was feasible and acceptable. Two previous incentive studies used text messages to inform participants about their incentive achievement of weight loss targets, and found it acceptable.[39 40] This extends the evidence base on the use of financial incentives as a complementary behaviour change strategy alongside other components.[19 43]

The financial incentive strategy was designed with future sustainability in mind and to be attractive to public sector funders. The text messages and incentive components are mostly automated, encourage self-management of weight and have a low administrative burden with one bank transfer pay out after 12 months, and verification of weight loss at three, six and 12 months. Yancy et al. similarly provided incentives at three and six months for their six months' incentive intervention[39]. John et al. asked participants to be weighed monthly, for which they received $20 per visit.[40] Few RCTs examine financial incentive strategies that are delivered for at least 12 months.[43] Two other studies providing financial incentives for 12 months provided an intensive financial incentive schedule either weekly[44] or monthly[45]. Previous studies typically provided participants with weekly weight loss goals such as 1lb (0.45kg) per week, on which financial incentives were contingent.[43] Whilst these goals are similar to the weight loss targets in the current study, there were no intermittent or weekly targets. A balance needs to be struck between having more regular weight measurements and more immediate pay-outs, costs and future sustainability.

For peer review only - http://bmjopen.bmj.com/site/about/guidelines.xhtml

Patel et al. applied a similar framing which they called a loss incentive[23]. University employees were allocated a monthly hypothetical incentive of $42 upfront and $1.40 was taken away each time their daily goal of 7000 steps was not met. The current study adds to the evidence base demonstrating the acceptability and feasibility of loss framed interventions for weight loss and in settings outside workplaces.

Conclusion

This weight management intervention recruited men from across the socioeconomic spectrum with the majority coming from disadvantaged areas, had an acceptable retention rate, and was broadly acceptable to most participants, with some weight loss benefits. A full trial of the study is warranted.

For peer review only

For peer review only - http://bmjopen.bmj.com/site/about/guidelines.xhtml

ACKNOWLEDGEMENT

We would like to acknowledge the following people who have contributed to this study:

All men who contributed to PPI and who participated in the study.

Colin Fowler from Men's Health Forum in Ireland, who was involved in the ROMEO systematic review that informed this study, commented on the grant application, protocol and final report. Together with Paula Carroll, they accessed wider PPI in Ireland.

Fiona Hogarth, Director Tayside Clinical Trials Unit, oversaw randomisation, data management and the running of the study from a Clinical Trials Unit perspective.

Hannah Collacott, who was employed as a research assistant at the Health Economics Research Unit, University of Aberdeen in 2016-2017. She undertook the think aloud interviews, PPI and assisted in the design and preliminary analysis of the DCE described in the final report to the funder.[10]

Clinical, Community and Academic Networks who advised our team on many aspects of this study, including: NHS Research Scotland Primary Care Network and the GP practices involved, Alliance Scotland, Community Health Councils and Health Centres

The following post-graduate Masters students, who accompanied the research assistants when recruiting men in the community in alphabetical order: Norelle Calder-Macphee, Ines Elenin Lucy Gunatillake, Bryony Lang, Heather Renwick and Amy Spiteri.

Karen Stanton, Lorna Kerr, Karen Murray, Siobhan McDermott, Suzannah Hunter, who provided administrative support.

The prior work of Professor Crombie and his team at Dundee who assisted with the grant application for this study.[11-13]

The Study Steering Committee:

Associate Professor Ed Juszczak, Director, NPEU Clinical Trials Unit, Nuffield Department of Population Health, University of Oxford (Chair).

Professor Kate Jolly, Professor of Public Health & Primary Care, University of Birmingham.

Mr Mark Kelvin, Programme Director (until 2018), Health and Social Care Alliance Scotland (the ALLIANCE).

Mr Andrew Shanahan, Director MAN v FAT - the weight loss website for men

For peer review only - http://bmjopen.bmj.com/site/about/guidelines.xhtml

Ms Martine Stead, Deputy Director, Institute for Social Marketing, University of Stirling

Miss Joyce Thompson Dietetic Consultant in Public Health Nutrition and Tayside Nutrition MCN Lead Clinician, NHS Tayside.

AUTHOR CONTRIBUTIONS

SD co-led the study with PH as joint Chief-Investigator and co-led the design and overall conduct of the study and led the writing of the manuscript, overseen by PH.

MM undertook the study set up, recruitment and data collection, co-ordinated various PPI activities, contributed substantially to qualitative and quantitative data analysis and interpretation. MvdP was a co-investigator, contributed to the design and the interpretation of findings and led the DCE and health economic analysis. MG was a co-investigator, contributed to the design and the interpretation of findings and led the development of the narrative SMS component, the related PPI. AA was a co-investigator, contributed to the design and the interpretation of findings. PC and MT linked to PPI networks, contributed to the design, and interpretation of the findings. AE was a co-investigator, contributed to the design and the interpretation of findings and oversaw the statistical analysis. NG and EC contributed to the analysis and interpretation of the qualitative data. CG was a co-investigator, facilitated PPI, contributed to the design, and interpretation of findings. FH was a co-investigator, oversaw the qualitative data collection and analysis and contributed to the interpretation of findings. AH undertook the statistical analysis at Tayside Clinical Trials Unit. CJ was a co-investigator, contributed to the design and the interpretation of findings and oversaw the automated SMS delivery system, recruitment tracking, the study website and integration of data with databases at Tayside Clinical Trials Unit. FK and MMcK were co-investigators and contributed to the design and the interpretation of findings. RS contributed to recruitment, data collection and undertaking qualitative interviews and contributed to the interpretation of findings.

FUNDING

This report presents independent research commissioned by the NIHR. The views and opinions expressed by authors in this publication are those of the authors and do not necessarily reflect those of the NHS, the NIHR, MRC, CCF, NETSCC, the Public Health Research programme or the Department of Health. The views and opinions expressed by the interviewees in this publication are those of the interviewees and do not necessarily reflect

For peer review only - http://bmjopen.bmj.com/site/about/guidelines.xhtml

those of the authors, those of the NHS, the NIHR, MRC, CCF, NETSCC, the Public Health Research programme or the Department of Health.

The Nursing Midwifery and Allied Health Professionals Research Unit, the Health Services Research Unit and Health Economics Research Unit are funded by the Chief Scientist Office of the Scottish Government Health and Social Care Directorate.

COMPETING INTERESTS

Dr Grindle is Director of Eos Digital Health Ltd which holds all rights to DNA - The Digital Narrative Approach deployed to design and write the narrative text intervention.

No other competing interests were declared.

DATA AVAILABILITY

Access to data can be arranged through the Co-PIs of the study: Prof. Pat Hoddinott, (University of Stirling, p.m.hoddinott@stir.ac.uk) and Dr. Stephan Dombrowski (University of New Brunswick, stephan.dombrowski@unb.ca) to discuss data sharing, data requirements and conflicts of interest, in line with any EU and other regulations, including ethics approvals.

For peer review only

REFERENCES

1. Ng M, Fleming T, Robinson M, et al. Global, regional, and national prevalence of overweight and obesity in children and adults during 1980–2013: a systematic analysis for the Global Burden of Disease Study 2013. *Lancet* 2014;384(9945):766-81. doi: 10.1016/S0140-6736(14)60460-8

2. The Scottish Government. The Scottish Health Survey 2016. Edinburgh, 2017.

3. Health and Social Care Information Centre. Health Survey for England 2016. London, 2016.

4. Robertson C, Archibald D, Avenell A, et al. Systematic reviews of and integrated report on the quantitative, qualitative and economic evidence base for the management of obesity in men. *HTA* 2014;18(35):1-424. doi: 10.3310/hta18350

5. Hall AK, Cole-Lewis H, Bernhardt JM. Mobile Text Messaging for Health: A Systematic Review of Reviews. *Annu Rev Public Health* 2015;36(1):393-415. doi: doi:10.1146/annurev-publhealth-031914-122855

6. Shaw R, Bosworth H. Short message service (SMS) text messaging as an intervention medium for weight loss: A literature review. *Health Informatics J* 2012;18(4):235-50. doi: 10.1177/1460458212442422

7. Palmer M, Sutherland J, Barnard S, et al. The effectiveness of smoking cessation, physical activity/diet and alcohol reduction interventions delivered by mobile phones for the prevention of non-communicable diseases: A systematic review of randomised controlled trials. *PLoS ONE* 2018;13(1):e0189801. doi: 10.1371/journal.pone.0189801

8. Ofcom. Adults' Media Use and Attitudes Report. In: Ofcom, ed. London, 2018.

9. Hinyard LJ, Kreuter MWJHE, Behavior. Using narrative communication as a tool for health behavior change: a conceptual, theoretical, and empirical overview. 2007;34(5):777-92.

10. Dombrowski SU, McDonald M, van der Pol M, et al. Feasibility trial of how to engage men with obesity in SMS (short message system) and incentive interventions for weight loss. in submission

For peer review only

For peer review only - http://bmjopen.bmj.com/site/about/guidelines.xhtml

11. Crombie IK, Irvine L, Falconer DW, et al. Alcohol and disadvantaged men: A feasibility trial of an intervention delivered by mobile phone. *Drug Alcohol Rev* 2017;36(4):468-76. doi: 10.1111/dar.12455

12. Irvine L, Crombie IK, Cunningham KB, et al. Modifying alcohol consumption to reduce obesity: A randomized controlled feasibility study of a complex community-based intervention for men. *Alcohol Alcohol* 2017;52(6):677-84. doi: 10.1093/alcalc/agx067

13. Crombie IK, Irvine L, Williams B, et al. Texting to Reduce Alcohol Misuse (TRAM): main findings from a randomized controlled trial of a text message intervention to reduce binge drinking among disadvantaged men. *Addiction* 2018;13(9):1609–18. doi: 10.1111/add.14229

14. Mantzari E, Vogt F, Shemilt I, et al. Personal financial incentives for changing habitual health-related behaviors: A systematic review and meta-analysis. *Prev Med* 2015;75:75-85. doi: 10.1016/j.ypmed.2015.03.001

15. Ryan M, Yi D, Avenell A, et al. Gaining pounds by losing pounds: preferences for lifestyle interventions to reduce obesity. *Health Econ Policy Law* 2014;10(2):161-82 doi: 10.1017/S1744133114000413

16. Burns RJ, Donovan AS, Ackermann RT, et al. A theoretically grounded systematic review of material incentives for weight loss: Implications for interventions. *Ann Behav Med* 2012;44(3):375-88. doi: 10.1007/s12160-012-9403-4

17. Giles EL, Robalino S, McColl E, et al. The effectiveness of financial incentives for health behaviour change: Systematic review and meta-analysis. *PLoS ONE* 2014;9(3):e90347. doi: 10.1371/journal.pone.0090347

18. Purnell JQ, Gernes R, Stein R, et al. A systematic review of financial incentives for dietary behavior change. *J Acad Nutr Diet* 2014;114(7):1023-35. doi: 10.1016/j.jand.2014.03.011

19. Ananthapavan J, Peterson A, Sacks G. Paying people to lose weight: the effectiveness of financial incentives provided by health insurers for the prevention and management of overweight and obesity – a systematic review. *Obes Rev* 2018;19(5):605-13. doi: 10.1111/obr.12657

For peer review only - http://bmjopen.bmj.com/site/about/guidelines.xhtml

20. Paloyo AR, Reichert AR, Reinermann H, et al. The causal link between financial incentives and weight loss: an evidence-based survey of the literature. *J Econ Surv* 2014;28(3):401-20. doi: 10.1111/joes.12010

21. Camerer CF, Loewenstein G, Rabin M. Advances in Behavioral Economics: Princeton University Press 2003.

22. Cawley J, Downs JS, Loewenstein G. Behavioral Economics and Obesity: Oxford University Press 2012.

23. Patel MS, Asch Md DA, Rosin R, et al. Framing financial incentives to increase physical activity among overweight and obese adults a randomized, controlled trial. *Ann Intern Med* 2016;164(6):385-94. doi: 10.7326/M15-1635

24. Teare M, Dimairo M, Shephard N, et al. Sample size requirements to estimate key design parameters from external pilot randomised controlled trials: a simulation study. *Trials* 2014;15(1):264. doi: 10.1186/1745-6215-15-264

25. The Scottish Government. Definition of SIMD quintiles. Edinburgh, 2013.

26. Dombrowski SU, Sniehotta FF, Johnston M, et al. Optimizing acceptability and feasibility of an evidence-based behavioral intervention for obese adults with obesity-related co-morbidities or additional risk factors for co-morbidities: An open-pilot intervention study in secondary care. *Patient Educ Couns* 2012;87(1):108-19. doi: 10.1016/j.pec.2011.08.003

27. Malterud K, Siersma VD, Guassora AD. Sample Size in Qualitative Interview Studies: Guided by Information Power. *Qual Health Res* 2016;26(13):1753-60. doi: 10.1177/1049732315617444

28. Eldridge SM, Chan CL, Campbell MJ, et al. CONSORT 2010 statement: extension to randomised pilot and feasibility trials. *BMJ* 2016;355:i5239. doi: 10.1136/bmj.i5239

29. Ritchie J, Spencer L. Qualitative Data Analysis for Applied Policy Research: SAGE 2002.

30. Gamble C, Dudley L, Allam A, et al. Health Services and Delivery Research. An evidence base to optimise methods for involving patient and public contributors in clinical trials: a mixed-methods study. Southampton (UK): NIHR Journals Library 2015.

For peer review only - http://bmjopen.bmj.com/site/about/guidelines.xhtml

31. Men's Health Forum. Mind your language: How men talk about mental health. London2018.

32. Hunt K, Wyke S, Gray CM, et al. A gender-sensitised weight loss and healthy living programme for overweight and obese men delivered by Scottish Premier League football clubs (FFIT): A pragmatic randomised controlled trial. *Lancet* 2014;383(9924):1211-21. doi: 10.1016/S0140-6736(13)62420-4

33. Archibald D, Douglas F, Hoddinott P, et al. A qualitative evidence synthesis on the management of male obesity. *BMJ Open* 2015;5(10):e008372. doi: 10.1136/bmjopen-2015-008372

34. Robertson C, Avenell A, Stewart F, et al. Clinical Effectiveness of Weight Loss and Weight Maintenance Interventions for Men: A Systematic Review of Men-Only Randomized Controlled Trials (The ROMEO Project). *Am J Mens Health* 2017;11(4):1096-123. doi: 10.1177/1557988315587550

35. Aveyard P, Lewis A, Tearne S, et al. Screening and brief intervention for obesity in primary care: a parallel, two-arm, randomised trial. *Lancet* 2016;388(10059):2492-500. doi: 10.1016/S0140-6736(16)31893-1

36. Haapala I, Barengo NC, Biggs S, et al. Weight loss by mobile phone: A 1-year effectiveness study. *Public Health Nutr* 2009;12(12):2382-91. doi: 10.1017/S1368980009005230

37. Shapiro JR, Koro T, Doran N, et al. Text4Diet: A randomized controlled study using text messaging for weight loss behaviors. *Prev Med* 2012;55(5):412-17. doi: 10.1016/j.ypmed.2012.08.011

38. Silina V, Tessma MK, Senkane S, et al. Text messaging (SMS) as a tool to facilitate weight loss and prevent metabolic deterioration in clinically healthy overweight and obese subjects: a randomised controlled trial. *Scand J Prim Health Care* 2017;35(3):262-70. doi: 10.1080/02813432.2017.1358435

39. Yancy Jr WS, Shaw PA, Wesby L, et al. Financial incentive strategies for maintenance of weight loss: results from an internet-based randomized controlled trial. *Nutrition & Diabetes* 2018;8(1):33. doi: 10.1038/s41387-018-0036-y

For peer review only - http://bmjopen.bmj.com/site/about/guidelines.xhtml

40. John LK, Loewenstein G, Troxel AB, et al. Financial incentives for extended weight loss: A randomized, controlled trial. *J Gen Intern Med* 2011;26(6):621-26. doi: 10.1007/s11606-010-1628-y

41. Leahey TM, Subak LL, Fava J, et al. Benefits of adding small financial incentives or optional group meetings to a web-based statewide obesity initiative. *Obesity* 2015;23(1):70-76. doi: 10.1002/oby.20937

42. Little P, Stuart B, Hobbs FR, et al. An internet-based intervention with brief nurse support to manage obesity in primary care (POWeR+): a pragmatic, parallel-group, randomised controlled trial. *Lancet Diabetes Endocrinol* 2016;4(10):821-28. doi: 10.1016/S2213-8587(16)30099-7

43. Paul-Ebhohimhen V, Avenell A. Systematic review of the use of financial incentives in treatments for obesity and overweight. *Obes Rev* 2008;9(4):355-67. doi: 10.1111/j.1467-789X.2007.00409.x

44. Jeffery RW, Wing RR, Thorson C, et al. Strengthening Behavioral Interventions for Weight Loss: A Randomized Trial of Food Provision and Monetary Incentives. *J Consult Clin Psychol* 1993;61(6):1038-45. doi: 10.1037/0022-006X.61.6.1038

45. Kramer FM, Jeffery RW, Snell MK, et al. Maintenance of successful weight loss over 1 year: Effects of financial contracts for weight maintenance or participation in skills training. *Behav Ther* 1986;17(3):295-301. doi: 10.1016/S0005-7894(86)80058-2

For peer review only

For peer review only - http://bmjopen.bmj.com/site/about/guidelines.xhtml

Table 1 Participant baseline characteristics

	SMS + I N=36	SMS only N=33	Control N=36	Total N=105
Age (years), mean (SD)	50.9 (14.2)[a]	52.5 (15.1)[b]	53.1 (10.1)	52.2 (13.1)[c]
Waist circumference (cm), mean SD	115.8 (10.0)	114.9 (12.7)	119.5 (12.2)	116.8 (11.8)
Weight (kg), mean (SD)	108.6 (16.4)	107.8 (20.2)	110.7 (19.0)	109.1 (18.4)
Height (cm), mean (SD)	175.9 (6.6)	175.2 (6.7)	173.8 (5.9)	175.0 (6.4)
BMI (kg/m^2), mean (SD)	35.1 (5.3)	35.1 (5.9)	36.7 (6.5)	35.7 (5.9)
**BMI (kg/m^2) categories, n (%)**				
<30	5 (13.9)	3 (9.1)	3 (8.3)	11 (10.5)
≥30->35	16 (44.4)	19 (57.6)	14 (38.9)	49 (46.7)
≥35->40	10 (27.8)	6 (18.2)	7 (19.4)	23 (21.8)
≥40->45	4 (11.1)	2 (6.1)	10 (27.8)	16 (15.2)
≥45->50	0 (0.0)	2 (6.1)	1 (2. 8)	3 (2.9)
≥50	1 (2.8)	1 (3.0)	1 (2. 8)	3 (2.9)
**SIMD deprivation category, n (%)**				
SIMD 1 (most disadvantaged)	11 (31.4)[a]	12 (36.4)	15 (41.7)	38 (36.5)[d]
SIMD 2	8 (22.9)[a]	9 (27.3)	7 (19.4)	24 (23.1)[d]
SIMD 3	6 (17.1)[a]	3 (9.1)	3 (8.3)	12 (11.5)[d]
SIMD 4	4 (11.4)[a]	4 (12.1)	6 (16.7)	14 (13.5)[d]
SIMD 5 (least disadvantaged)	6 (17.5)[a]	5 (15.2)	5 (13.9)	16 (15.4)[d]

For peer review only - http://bmjopen.bmj.com/site/about/guidelines.xhtml

**Marital status, n (%)**				
Cohabiting	4 (11.1)	2 (6.3) [e]	5 (13.8)	11 (10.6)[d]
Divorced	2 (5.6)	0 (0.0) [e]	2 (5.6)	4 (3.9)[d]
Married	22 (61.1)	21 (65.6)[e]	17 (47.2)	60 (57.7)[d]
Separated	1 (2.8)	3 (9.4)[e]	2 (5.6)	6 (5.8)[d]
Single	7 (19.4)	5 (15.6)[e]	8 (22.2)	20 (19.2)[d]
Widowed	0 (0)	1 (3.1)[e]	1 (2.8)	2 (19.2)[d]
Prefer not to say	0 (0.0)	0 (0.0)[e]	1 (2.8)	1 (1.0)[d]
**Comorbidities, n (%)**				
Arthritis	11 (30.6)	3 (9.1)	8 (22.2)	22 (21.0)
Cancer	0 (0.0)	0 (0.0)	1 (2.8)	1 (1.0)
Diabetes	4 (11.1)	5 (15.2)	6 (16.7)	15 (14.3)
Myocardial infarction	2 (5.6)	1 (3.0)	0 (0.0)	3 (2.9)
Hypertension	7 (19.4)	8 (24.2)	7 (19.4)	22 (21.0)
Stroke (including TIA)	1 (2.8)	1 (3.0)	0 (0.0)	2 (1.9)
**Ethnic group, n (%)**				
Asian	2 (5.6)	1 (3.1)[e]	1 (2.8)	4 (3.8)[d]
Black	2 (5.6)	1 (3.1)[e]	1 (2.8)	4 (3.8)[d]
White	32 (88.8)	29 (90.7)[e]	34 (94.4)	95 (91.4)[d]
Prefer not to say	0 (0.0)	1 (3.1) [e]	0 (0.0)	1 (1.0)[d]
**Education**				

For peer review only - http://bmjopen.bmj.com/site/about/guidelines.xhtml
For peer review only

Bachelor Degree (=SVQ5)	7 (19.4)	6 (19.5)[b]	11 (30.6)	24 (23.3)[f]
HNC/ HND (=SVQ4)	4 (11.1)	6 (19.5)[b]	2 (5.6)	12 (11.6)[f]
Higher Grade/ Advanced Higher/ A-Level or equivalent (=SVQ3)	3 (8.3)	5 (16.1)[b]	1 (2.8)	9 (8.7)[f]
Masters/ PhD or equivalent	1 (2.8)	1 (3.2)[b]	3 (8.3)	5 (4.8)[f]
No formal qualifications	7 (19.4)	3 (9.7)[b]	10 (27.8)	20 (19.4)[f]
Standard Grade/GCSE/ Intermediate 1 or 2	6 (16.7)	7 (22.6)[b]	4 (11.1)	17 (16.5)[f]
Still studying	2 (5.6)	1 (3.2)[b]	3 (8.3)	6 (5.8)[f]
Vocational qualifications (=SVQ1+2)	4 (11.1)	1 (3.2)[b]	0 (0.0)	5 (4.8)[f]
Other	0 (0.0)	1 (3.2)[b]	0 (0.0)	1 (1.0)[f]
Prefer not to say	2 (5.6)	0 (0.0)[b]	2 (5.6)	4 (3.9)[f]
**Working status, n (%)**				
Full time student	4 (11.1)	2 (6.3)[e]	1 (2.8)	7 (6.7)[d]
Employed - Full time (30+ hours per week)	16 (44.4)	16 (50.0)[e]	18 (50.0)	50 (48.1)[d]
Employed - Part time (8-29 hours per week)	3 (8.3)	3 (9.4)[e]	0 (0.0)	6 (5.8)[d]
Self-employed	3 (8.3)	3 (9.4)[e]	1 (2.8)	7 (6.7)[d]
Not in paid work	2 (5.6)	2 (6.2)[e]	12 (33.3)	16 (15.4)[d]
Retired	8 (22.2)	6 (18.7)[e]	4 (11.1)	18 (17.3)[d]
Household size, mean (SD)	2.6 (1.3)	2.6 (1.0)	2.4 (1.4)	2.5 (1.3)

For peer review only - http://bmjopen.bmj.com/site/about/guidelines.xhtml

Note: [a] N=35, [b] N=31, [c] N=102, [d] N=104, [e] N=32, [f] N=103; BMI = Body Mass Index, CM = centimetres, GCSE = General Certificate of Secondary Education, kg = kilogram, N = overall participants, n = participants within specific category, SD = Standard Deviation, SIMD = Scottish index of multiple deprivation 1 represents the most disadvantaged area; quintile SIMD 5 represents the least disadvantaged area, SVQ = Scottish Vocational Qualification

For peer review only

Table 2 Programme satisfaction, and contamination at three, six and 12 months

	3 months		6 months		12 months		
	SMS + I (N=31)	SMS only (N=27)	SMS + I (N=23)	SMS only (N=21)	SMS + I (N=22)	SMS only (N=26)	Control (N=30)
Programme satisfaction (0-100), mean (SD)	80.3 (21.1)	75.0 (22.2)	76.2 (29.6)	79.0 (20.7)	80.9 (20.0)	77.0 (20.8)	87.3 (17.5)[a]
**Programme has been… (1 = low, 5 = high), mean (SD)**							
understandable	4.5 (0.9)	4.4 (0.8)[b]	4.4 (1.2)[c]	4.4 (1.2)	4.6 (1.1)[d]	4.6 (1.1)[e]	4.6 (0.9)[a]
useful	4.4 (0.9)	4.0 (1.0)	4.1 (1.3)	4.1 (1.1)[d]	4.1 (1.2)	4.0 (1.0)	4.3 (0.9)
helpful	4.3 (0.9)[f]	3.9 (1.0)	4.1 (1.3)	4.1 (1.1)[d]	4.1 (1.10)	4.0 (1.0)	4.3 (0.9)
interesting	4.3 (1.0)	4.2 (1.1)	4.2 (1.1)	4.4 (1.3)[d]	4.3 (0.8)	4.3 (1.2)	4.5 (1.0)
relevant	4.2 (1.1)	3.9 (1.2)	4.0 (1.3)	4.3 (1.1)[d]	4.1 (1.1)	4.2 (1.1)	4.5 (1.0)
**Helpfulness (1 = low, 5 = high), mean (SD)**							
Text messages	3.4 (1.5)	3.1 (1.3)	3.1 (1.3)	3.6 (1.3)	3.4 (1.3)	3.3 (1.4)	n/a

Website	3.3 (1.2)[g]	3.3 (0.9)[b]	3.3 (1.2)	3.2 (0.8)[d]	3.6 (1.0)	3.4 (0.7)[h]	3.5 (1.1) [b]
Pedometer	4.1 (1.2)[f]	4.1 (0.9)	3.7 (1.4)	4.0 (1.0)	3.9 (1.2)[i]	4.0 (1.0)[e]	3.7, (1.4)
**Met other men in programme, n (%)**							
Yes	0 (0.0)	0 (0.0)	1 (4.3)	0 (0.0)	1 (4.5)	0 (0.0)	1 (3.3)

Note. [a] N=29, [b] N=26, [c] N=22, [d] N=20, [e] N=25, [f] N=30, [g] N=28, [h] N=24, SD = Standard Deviation.

For peer review only - http://bmjopen.bmj.com/site/about/guidelines.xhtml

Table 3 Weight change at three, six, and 12 months

	3 months		6 months		12 months		
	SMS + I<br>N=36	SMS only<br>N=33	SMS + I<br>N=36	SMS only<br>N=33	SMS + I<br>N=36	SMS only<br>N=33	Control<br>N=36
**Weight change (kg), mean (SD)**							
Observed cases only	2.79 (3.50)[a]	-1.97 (3.97)[b]	-4.59 (5.62)[c]	-3.30 (4.92)[d]	-3.93 (5.74)[c]	-1.64 (5.64)[b]	-1.06 (6.29)[e]
BOCF	-2.40 (3.38)	-1.55 (3.60)	-2.93 (4.98)	-2.10 (4.21)	-2.51 (4.94)	-1.29 (5.03)	-0.86 (5.64)
LOCF	-2.40 (3.38)	-1.86 (3.88)	-3.38 (4.92)	-2.21 (4.39)	-2.98 (4.91)	-1.33 (5.04)	-0.86 (5.64)
**Weight change (%), mean (SD)**							
Observed cases only	-2.54 (3.47)[a]	-1.95 (3.72)[b]	-4.20 (5.54)[c]	-3.02 (4.22)[d]	-3.51 (5.83)[c]	-1.51 (4.65)[b]	-1.00 (5.31)[e]
BOCF	-2.18 (3.33)	-1.53 (3.39)	-2.69 (4.84)	-1.92 (3.65)	-2.24 (4.93)	-1.19 (4.16)	-0.80 (4.77)
LOCF	-2.18 (3.33)	-1.53 (3.39)	-3.11 (4.80)	-2.06 (3.85)	-2.68 (4.92)	-1.22 (4.16)	-0.80 (4.77)
**Weight change categories (observed cases only), n (%)**							

weight gain	6 (19.4)[a]	8 (30.8)[b]	2 (8.7)[c]	6 (28.6)[d]	5 (21.7)[c]	10 (38.5)[b]	14 (48.3)[e]
0-<3% weight loss	13 (41.9)[a]	10 (38.5)[b]	10 (43.5)[c]	7 (33.3)[d]	5 (21.7)[c]	9 (34.6)[b]	7 (24.1)[e]
≥3-<5% weight loss	4 (12.9)[a]	4 (15.4)[b]	3 (13.0)[c]	2 (9.5)[d]	3 (13.0)[c]	4 (15.4)[b]	0 (0.0)[e]
≥5-<10% weight loss	8 (25.8)[a]	2 (7.7)[b]	3 (13.0)[c]	4 (19.1)[d]	7 (30.4)[c]	2 (7.7)[b]	6 (20.7)[e]
≥10% weight loss	0 (0.0)[a]	2 (7.7)[b]	5 (21.7)[c]	2 (9.5)[d]	3 (13.0)[c]	1 (3.9)[b]	2 (6.9)
**BMI change, mean (SD)**							
Observed cases only	-0.89 (1.1) [a]	-0.73 (1.32)[b]	-1.45 (1.79)[c]	-1.04 (1.49)[d]	-1.24 (1.89)[c]	-0.49 (1.68)[b]	-0.47 (2.13)[e]
BOCF	-0.75 (1.07)	-0.59 (1.22)	-0.92 (1.58)	-0.66 (1.28)	-0.78 (1.60)	-0.37 (1.50)	-0.37 (1.96)
LOCF	-0.75 (1.07)	-0.59 (1.22)	-0.93 (1.56)	-0.69 (1.34)	-0.93 (1.59)	-0.38 (1.50)	-0.37 (1.96)
**Waist circumference change (cm), mean (SD)**							
Observed cases only	-3.74 (4.62) [a]	-3.14 (4.3)[b]	-4.70 (6.29)[c]	-3.14 (4.34)[d]	-4.40 (6.08)[c]	-2.30 (4.37)[b]	-2.26 (4.97)[e]
BOCF	-3.22 (4.47)	-2.49 (3.9)	-3.00 (5.49)	-2.00 (3.76)	-2.81 (5.82)	-1.82 (3.98)	-1.82 (4.53)
LOCF	-3.22 (4.47)	-2.49 (3.9)	-3.71 (5.76)	-2.23 (4.03)	-3.51 (4.48)	-1.85 (3.97)	-1.82 (4.53)

Note. [a] N=31, [b] N=26, [c] N=23, [d] N=21, BOCF = baseline observation carried forward, cm = centimetres, LOCF = last observation carried forward, kg = kilogram, M = mean, SD = Standard Deviation.

For peer review only

For peer review only - http://bmjopen.bmj.com/site/about/guidelines.xhtml

Figure 1 Consort flow diagram

For peer review only

For peer review only - http://bmjopen.bmj.com/site/about/guidelines.xhtml

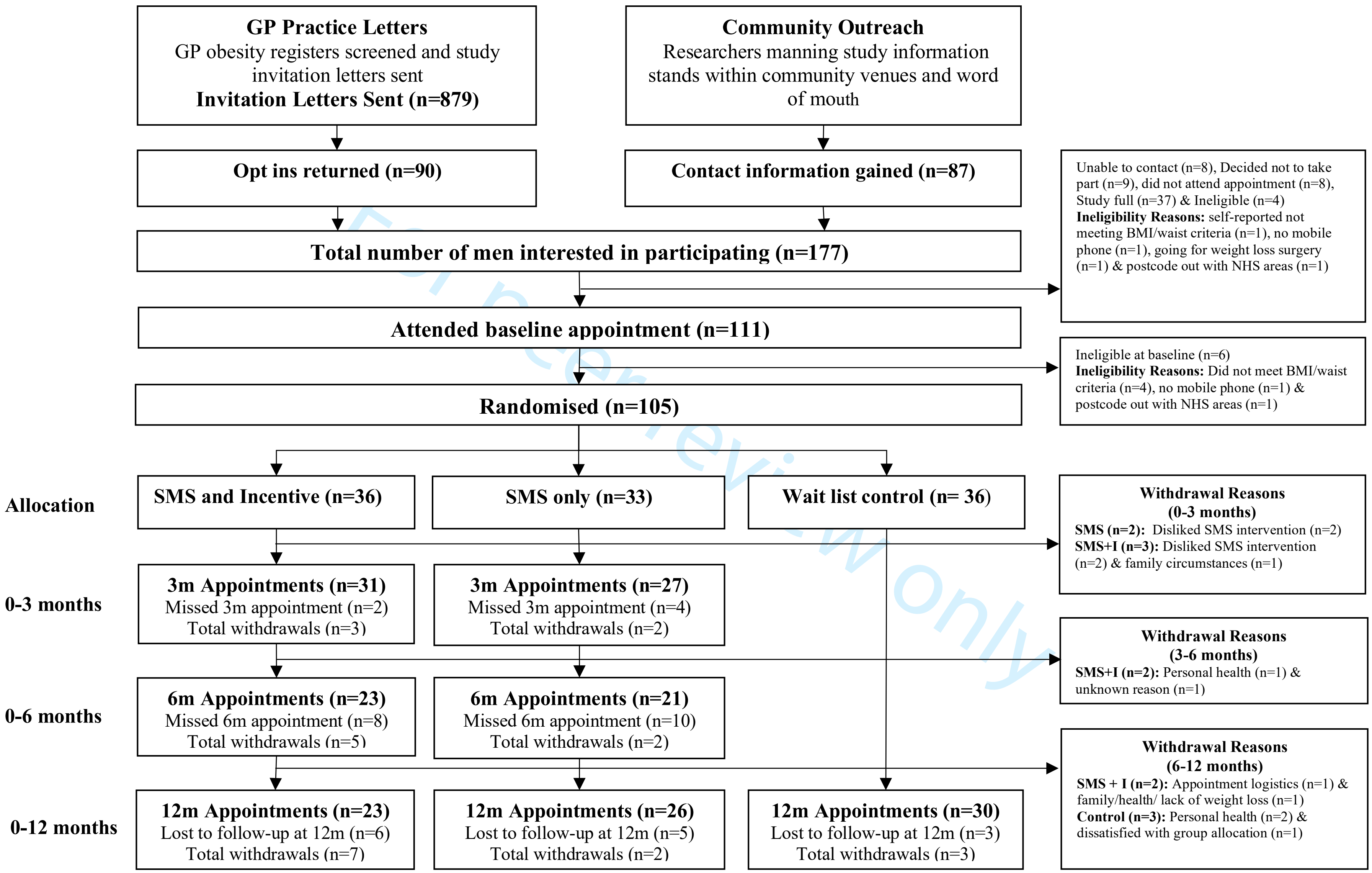
GP Practice Letters
GP obesity registers screened and study invitation letters sent
Invitation Letters Sent (n=879)
Community Outreach
Researchers manning study information stands within community venues and word of mouth
Opt ins returned (n=90)
Contact information gained (n=87)
Unable to contact (n=8), Decided not to take part (n=9), did not attend appointment (n=8), Study full (n=37) & Ineligible (n=4)
Ineligibility Reasons: self-reported not meeting BMI/waist criteria (n=1), no mobile phone (n=1), going for weight loss surgery (n=1) & postcode out with NHS areas (n=1)
Total number of men interested in participating (n=177)
Attended baseline appointment (n=111)
Ineligible at baseline (n=6)
Ineligibility Reasons: Did not meet BMI/waist criteria (n=4), no mobile phone (n=1) & postcode out with NHS areas (n=1)
Randomised (n=105)
Allocation
SMS and Incentive (n=36)
SMS only (n=33)
Wait list control (n= 36)
Withdrawal Reasons
(0-3 months)
SMS (n=2): Disliked SMS intervention (n=2)
SMS+I (n=3): Disliked SMS intervention (n=2) & family circumstances (n=1)
0-3 months
3m Appointments (n=31)
Missed 3m appointment (n=2)
Total withdrawals (n=3)
3m Appointments (n=27)
Missed 3m appointment (n=4)
Total withdrawals (n=2)
Withdrawal Reasons
(3-6 months)
SMS+I (n=2): Personal health (n=1) & unknown reason (n=1)
0-6 months
6m Appointments (n=23)
Missed 6m appointment (n=8)
Total withdrawals (n=5)
6m Appointments (n=21)
Missed 6m appointment (n=10)
Total withdrawals (n=2)
Withdrawal Reasons
(6-12 months)
SMS + I (n=2): Appointment logistics (n=1) & family/health/ lack of weight loss (n=1)
Control (n=3): Personal health (n=2) & dissatisfied with group allocation (n=1)
0-12 months
12m Appointments (n=23)
Lost to follow-up at 12m (n=6)
Total withdrawals (n=7)
12m Appointments (n=26)
Lost to follow-up at 12m (n=5)
Total withdrawals (n=2)
12m Appointments (n=30)
Lost to follow-up at 12m (n=3)
Total withdrawals (n=3)

1

Online supplement 1: Incentive strategy contingent on meeting weight loss targets over 12 months

	Money secured	Money lost
3 months: £50 – target 5% weight loss		
<5%	£0	£50
≥5%	£50	£0
6 months: £150 – target 10%		
<5%	£0	£150
5%	£75	£75
6%	£90	£60
7%	£105	£45
8%	£120	£30
9%	£135	£15
≥10%	£150	£0
12 months: £200 – target 10%		
<5%	£0	£200
5%	£100	£100
6%	£120	£80
7%	£140	£60
8%	£160	£40
9%	£180	£20
≥10%	£200	£0

For peer review only

For peer review only - http://bmjopen.bmj.com/site/about/guidelines.xhtml

Online supplement 2 Intervention engagement over time for narrative texts and study webpage

	0-3 months		3-6 months		6-12 months	
	SMS + I N=36	SMS only N=33	SMS + I N=36	SMS only N=33	SMS + I N=36	SMS only N=33
Replies to texts, count	289	81	10	6	28	11
Replies to texts, mean (SD)	8.0 (24.2)	2.5 (7.9)	0.3 (0.7)	0.3 (1.0)	0.8 (3.3)	0.3 (1.0)
**Text replies, n (%)**						
0 replies	21 (58.3)	23 (69.7)	30 (83.3)	31 (93.9)	28 (77.8)	28 (84.8)
1-5 replies	9 (25.0)	7 (22.3)	6 (16.7)	2 (6.1)	7 (19.4)	5 (15.2)
6-10 replies	2 (5.6)	1 (3.0)	0 (0.0)	0 (0.0)	0 (0.0)	0 (0.0)
11-15 replies	0 (0.0)	0 (0.0)	0 (0.0)	0 (0.0)	0 (0.0)	0 (0.0)
16-20 replies	1 (2.8)	1 (3.0)	0 (0.0)	0 (0.0)	0 (0.0)	0 (0.0)
>20 replies	3 (8.3)	1 (3.0)	0 (0.0)	0 (0.0)	1 (2.8)	0 (0.0)

For peer review only - http://bmjopen.bmj.com/site/about/guidelines.xhtml

Stopping texts, n (%)	1 (2.8)	2 (6.1)	3 (8.3)	3 (9.1)	1 (2.8)	1 (3.0)
Withdrawal due to texts (self-report)	2 (5.6)	2 (6.1)	0 (0.0)	0 (0.0)	0 (0.0)	0 (0.0)
**Participants using website self-monitoring features, n (%)**						
Weight	8 (22.2)	5 (15.2)	6 (16.7)	3 (9.1)	4 (11.1)	3 (9.1)
Steps	10 (27.8)	6 (18.2)	7 (19.4)	2 (6.1)	3 (8.3)	2 (6.1)
Waistline	4 (11.1)	3 (9.1)	3 (8.3)	0 (0.0)	1 (2.8)	0 (0.0)
Belt notches	2 (5.6)	0 (0.0)	2 (5.6)	0 (0.0)	0 (0.0)	0 (0.0)
**Number self-monitoring features used, n (%)**						
None	24 (66.7)	26 (78.8)	28 (77.8)	30 (90.1)	31 (86.1)	29 (87.9)
One	5 (13.9)	2 (6.1)	2 (5.6)	1 (3.1)	3 (8.3)	3 (9.1)
Two	3 (8.3)	3 (9.1)	3 (8.3)	2 (6.1)	1 (2.8)	1 (3.0)

For peer review only

Three	3 (8.3)	2 (6.1)	2 (5.6)	0 (0.0)	1 (2.8)	0 (0.0)
Four	1 (2.8)	0 (0.0)	1 (2.8)	0 (0.0)	0 (0.0)	0 (0.0)
**Number of entries on self-monitoring feature, count**						
Weight	69	115	45	23	42	46
Steps	419	296	350	102	397	74
Waistline	4	7	5	0	2	0
Belt notches	8	0	4	0	0	0

Note. M = mean, SD = Standard Deviation.

For peer review only

Online supplement 3 Satisfaction and helpfulness ratings for overall programme and narrative texts at 12 months by deprivation status

	SMS+I		SMS only		Total	
	SIMD 1, 2 (n=14)	SIMD 3+ (n=7)	SIMD 1, 2 (n=16)	SIMD 3+ (n=10)	SIMD 1, 2 (n=30)	SIMD 3+ (n=17)
Satisfaction programme[a]	81.8, 21.7	79.9, 19.2	74.6, 23.5	81.0, 16.0	77.9, 22.6	80.5, 16.8
Helpfulness programme[b]	4.2, 1.1	3.9, 1.1	3.9, 1.1	4.2, 1.0	4.1, 1.1	4.1, 1.0
Helpfulness of narrative texts[b]	3.4, 1.3	3.4, 1.4	3.0, 1.5	3.8, 1.2	3.2, 1.4	3.6, 1.3

Note. All data mean (SD), [a] scored 0-100 (higher = more satisfied), [b] scored 1-5 **(**1 = low, 5 = high**),** SIMD = Scottish index of multiple deprivation 1 represents the most disadvantaged area; quintile SIMD 5 represents the least disadvantaged area SMS = Short message service, SMS+I = SMS plus incentive group.

For peer review only

CONSORT 2010 checklist of information to include when reporting a pilot or feasibility trial*

Section/Topic	Item No	Checklist item	Reported on page No
**Title and abstract**			
	1a	Identification as a pilot or feasibility randomised trial in the title	1
	1b	Structured summary of pilot trial design, methods, results, and conclusions (for specific guidance see CONSORT abstract extension for pilot trials)	3, 4
**Introduction**			
Background and objectives	2a	Scientific background and explanation of rationale for future definitive trial, and reasons for randomised pilot trial	6, 7
	2b	Specific objectives or research questions for pilot trial	7
**Methods**			
Trial design	3a	Description of pilot trial design (such as parallel, factorial) including allocation ratio	7
	3b	Important changes to methods after pilot trial commencement (such as eligibility criteria), with reasons	n/a
Participants	4a	Eligibility criteria for participants	8
	4b	Settings and locations where the data were collected	8,9
	4c	How participants were identified and consented	8,9
Interventions	5	The interventions for each group with sufficient details to allow replication, including how and when they were actually administered	10, 11
Outcomes	6a	Completely defined prespecified assessments or measurements to address each pilot trial objective specified in 2b, including how and when they were assessed	11-13
	6b	Any changes to pilot trial assessments or measurements after the pilot trial commenced, with reasons	n/a
	6c	If applicable, prespecified criteria used to judge whether, or how, to proceed with future definitive trial	7
Sample size	7a	Rationale for numbers in the pilot trial	8
	7b	When applicable, explanation of any interim analyses and stopping guidelines	n/a
Randomisation:			
Sequence generation	8a	Method used to generate the random allocation sequence	9
	8b	Type of randomisation(s); details of any restriction (such as blocking and block size)	9
Allocation concealment mechanism	9	Mechanism used to implement the random allocation sequence (such as sequentially numbered containers), describing any steps taken to conceal the sequence until interventions were assigned	9

For peer review only

Implementation	10	Who generated the random allocation sequence, who enrolled participants, and who assigned participants to interventions	9
Blinding	11a	If done, who was blinded after assignment to interventions (for example, participants, care providers, those assessing outcomes) and how	9
	11b	If relevant, description of the similarity of interventions	n/a
Statistical methods	12	Methods used to address each pilot trial objective whether qualitative or quantitative	11-13
**Results**			
Participant flow (a diagram is strongly recommended)	13a	For each group, the numbers of participants who were approached and/or assessed for eligibility, randomly assigned, received intended treatment, and were assessed for each objective	40
	13b	For each group, losses and exclusions after randomisation, together with reasons	40
Recruitment	14a	Dates defining the periods of recruitment and follow-up	15
	14b	Why the pilot trial ended or was stopped	15
Baseline data	15	A table showing baseline demographic and clinical characteristics for each group	31-33
Numbers analysed	16	For each objective, number of participants (denominator) included in each analysis. If relevant, these numbers should be by randomised group	31-45
Outcomes and estimation	17	For each objective, results including expressions of uncertainty (such as 95% confidence interval) for any estimates. If relevant, these results should be by randomised group	15-19
Ancillary analyses	18	Results of any other analyses performed that could be used to inform the future definitive trial	n/a
Harms	19	All important harms or unintended effects in each group (for specific guidance see CONSORT for harms)	19
	19a	If relevant, other important unintended consequences	n/a
**Discussion**			
Limitations	20	Pilot trial limitations, addressing sources of potential bias and remaining uncertainty about feasibility	20
Generalisability	21	Generalisability (applicability) of pilot trial methods and findings to future definitive trial and other studies	22
Interpretation	22	Interpretation consistent with pilot trial objectives and findings, balancing potential benefits and harms, and considering other relevant evidence	22
	22a	Implications for progression from pilot to future definitive trial, including any proposed amendments	22
**Other information**			
Registration	23	Registration number for pilot trial and name of trial registry	4
Protocol	24	Where the pilot trial protocol can be accessed, if available	7
Funding	25	Sources of funding and other support (such as supply of drugs), role of funders	24,25
	26	Ethical approval or approval by research review committee, confirmed with reference number	7

For peer review only

Citation: Eldridge SM, Chan CL, Campbell MJ, Bond CM, Hopewell S, Thabane L, et al. CONSORT 2010 statement: extension to randomised pilot and feasibility trials. BMJ. 2016;355.
*We strongly recommend reading this statement in conjunction with the CONSORT 2010, extension to randomised pilot and feasibility trials, Explanation and Elaboration for important clarifications on all the items. If relevant, we also recommend reading CONSORT extensions for cluster randomised trials, non-inferiority and equivalence trials, non-pharmacological treatments, herbal interventions, and pragmatic trials. Additional extensions are forthcoming: for those and for up to date references relevant to this checklist, see www.consort-statement.org.

For peer review only

BMJ Open

Game of Stones: Feasibility randomised controlled trial of how to engage men with obesity in text message and incentive interventions for weight loss

Journal:	*BMJ Open*
Manuscript ID	bmjopen-2019-032653.R1
Article Type:	Original research
Date Submitted by the Author:	12-Nov-2019
Complete List of Authors:	Dombrowski, Stephan U; University of New Brunswick Fredericton, Kinesiology<br>McDonald, Matthew; University of Stirling<br>van der Pol, Marjon; University of Aberdeen, Health Economics Research Unit<br>Grindle, Mark; University of the Highlands and Islands<br>Avenell, Alison; University of Aberdeen<br>Carroll, Paula; Mens Health Forum in Ireland<br>Calveley, Eileen; University of Stirling<br>Elders, A; Glasgow Caledonian University School of Health and Life Sciences, NMAHP Research Unit ; Glasgow Caledonian University<br>Glennie, Nicola ; University of Stirling<br>Gray, Cindy; University of Glasgow, Institute of Health and Wellbeing<br>Harris, Fiona; University of Stirling,<br>Hapca, Adrian; University of Dundee, Population Health Sciences<br>Jones, Claire; University of Dundee<br>Kee, Frank; Queen's University Belfast, UKCRC Centre of Excellence for Public Health (NI)<br>McKinley, Michelle C.; Queen's University Belfast<br>Skinner, Rebecca; University of Stirling<br>Tod, Martin; Mens Health Forum<br>Hoddinott, Pat; University of Stirling, Nursing, Midwifery and Allied Health Professional Research Unit
**Primary Subject Heading**:	Public health
Secondary Subject Heading:	Public health
Keywords:	obesity, men, feasibility, trial, SMS, weight loss

SCHOLARONE™
Manuscripts

I, the Submitting Author has the right to grant and does grant on behalf of all authors of the Work (as defined in the below author licence), an exclusive licence and/or a non-exclusive licence for contributions from authors who are: i) UK Crown employees; ii) where BMJ has agreed a CC-BY licence shall apply, and/or iii) in accordance with the terms applicable for US Federal Government officers or employees acting as part of their official duties; on a worldwide, perpetual, irrevocable, royalty-free basis to BMJ Publishing Group Ltd ("BMJ") its licensees and where the relevant Journal is co-owned by BMJ to the co-owners of the Journal, to publish the Work in this journal and any other BMJ products and to exploit all rights, as set out in our licence.

The Submitting Author accepts and understands that any supply made under these terms is made by BMJ to the Submitting Author unless you are acting as an employee on behalf of your employer or a postgraduate student of an affiliated institution which is paying any applicable article publishing charge ("APC") for Open Access articles. Where the Submitting Author wishes to make the Work available on an Open Access basis (and intends to pay the relevant APC), the terms of reuse of such Open Access shall be governed by a Creative Commons licence – details of these licences and which Creative Commons licence will apply to this Work are set out in our licence referred to above.

Other than as permitted in any relevant BMJ Author's Self Archiving Policies, I confirm this Work has not been accepted for publication elsewhere, is not being considered for publication elsewhere and does not duplicate material already published. I confirm all authors consent to publication of this Work and authorise the granting of this licence.

For peer review only

Game of Stones: Feasibility randomised controlled trial of how to engage men with obesity in text message and incentive interventions for weight loss

Stephan U Dombrowski[1, 2], Matthew McDonald[3], Marjon van der Pol[4], Mark Grindle[5], Alison Avenell[6], Paula Carroll[7], Eileen Calveley[3], Andrew Elders[8], Nicola Glennie[3], Cindy M Gray[9], Fiona M Harris[3], Adrian Hapca[10], Claire Jones[11], Frank Kee[12], Michelle C McKinley[13], Rebecca Skinner[2], Martin Tod[13], Pat Hoddinott[3]

Corresponding author:

Stephan U Dombrowski

Assistant Professor

Faculty of Kinesiology

90 Mackay Drive

Fredericton, New Brunswick,

Canada, E3B 5A3,

Email: stephan.dombrowski@unb.ca

Tel: 1 506 453 4803

[1]Faculty of Kinesiology, University of New Brunswick

[2]Division of Psychology, University of Stirling

[3]Nursing, Midwifery and Allied Health Professions Research Unit, University of Stirling

[4]Health Economics Research Unit, University of Aberdeen

[5]Institute for Health Research and Innovation, University of the Highlands and Islands

[6]Health Services Research Unit, University of Aberdeen

[7]Men's Health Forum in Ireland

[8]Nursing, Midwifery and Allied Health Professions Research Unit, Glasgow Caledonian University

For peer review only

[9]Institute of Health & Wellbeing, University of Glasgow

[10]Tayside Clinical Trials Unit, University of Dundee

[11]Health Informatics Centre, University of Dundee

[12] Centre for Public Health, Queen's University Belfast

[13]The Men's Health Forum (GB), London

Word count: 5624

Key words: obesity, men, feasibility, trial, SMS, weight loss, behaviour change, financial incentives

For peer review only

ABSTRACT

Objectives

To examine the acceptability and feasibility of narrative text messages with or without financial incentives to support weight loss for men.

Design

Individually randomised three-arm feasibility trial with 12 months' follow-up.

Setting

Two sites in Scotland with high levels of disadvantage according to Scottish Index for Multiple Deprivation (SIMD).

Participants

Men with obesity (n=105) recruited through community outreach and general practitioner registers.

Interventions

Participants randomised to a) narrative text messages plus financial incentive for 12 months (SMS+I), b) narrative text messages for 12 months (SMS only), or c) waiting list control.

Outcomes

Acceptability and feasibility of recruitment, retention, intervention components and trial procedures assessed by analysing quantitative and qualitative data at three, six and 12 months.

Results

105 men were recruited, 60% from more disadvantaged areas (SIMD quintiles 1 or 2). Retention at 12 months was 74%. Fewer SMS+I participants group (64%) completed 12-month assessments compared to SMS only (79%) and control (83%). Narrative texts were acceptable to many men, but some reported negative reactions. No evidence emerged that level of disadvantage was related to acceptability of narrative texts. Eleven SMS+I participants (31%) successfully met or partially met weight loss targets. The cost of the incentive per participant was £81.94 (95% CI £34.59-£129.30). Incentives were acceptable, but improving health was reported as the key motivator for weight loss. All groups lost

For peer review only

weight (SMS+I -2.51kg (SD=4.94); SMS only -1.29kg (SD=5.03); control -0.86kg (SD=5.64) at 12 months).

Conclusions

This three-arm weight management feasibility trial recruited and retained men from across the socioeconomic spectrum with the majority from areas of disadvantage, was broadly acceptable to most participants and feasible to deliver.

Trial registration

ClinicalTrials.gov Identifier: NCT03040518

Funding details

Funded by the National Institute for Health Research Public Health Research Programme (14/185).

For peer review only

STRENGTHS AND LIMITATIONS OF THIS STUDY

- This weight management study for men with obesity recruited men across the socio-economic spectrum, with a specific focus on more disadvantaged areas, to a randomised controlled feasibility study examining SMS with or without endowment incentives.
- Acceptability and feasibility were established using a multi-lens perspective drawing on quantitative, qualitative and trial procedure data.
- Effectiveness of intervention components will need to be established in a full multi-centre trial.

For peer review only

BACKGROUND

Obesity is associated with an increased risk of serious health conditions such as type 2 diabetes, cardiovascular disease and some cancers.[1] In 2016, 26% of men in the UK were classified as obese, and men were less likely to be a healthy weight than women (31% of men compared with 35% of women).[2 3]

Despite the growing prevalence of obesity, men contribute a disproportionately low number of participants to evidence-based weight management programmes.[4] Most interventions are designed for mixed sex populations, but systematic review evidence suggests that, compared to women, men often benefit from different ways of providing interventions.[4]

Text message interventions

The evidence for text message interventions supporting changes in lifestyle behaviours, including behaviour change for weight loss, is promising.[5-7] However, systematic reviews[4-7] report no text message delivered trials that target weight loss designed for men only. Text-based interventions can reach large numbers of people, including men from disadvantaged backgrounds,[8] and mobile technologies such as standard mobile phones allow delivery of evidence-based strategies anywhere and anytime.

Narrative approaches to promoting behaviour change have been suggested as a tool for communication-based interventions.[9] Narrative SMS can be broadly defined as interactive life stories, which are based around a group of characters with whom recipients can identify.[10] Text message based interventions using narratives have been used to engage hard to reach men in moderating their alcohol consumption, and were found to be acceptable.[11-13] Narrative approaches to weight management in men may be a promising tool to support behaviour and weight change.

Financial incentive interventions

Systematic review evidence of financial incentives for behaviour change highlights the potential for incentives to change behaviours in low-income adults[14] and help reduce health inequalities.[15] However, little evidence exists that focuses on using financial incentives to support weight management in individuals from disadvantaged backgrounds[16 17], particularly for men[4]. A discrete choice experiment found that paying people (£10-£30/week varying by age and weight) to take part in diet and physical activity (PA) interventions is likely to improve uptake, adherence and maintenance of behaviour change.[18] Moreover, the evidence for financial incentives for weight loss is growing.[19-22] In particular, deposit contracts, where

For peer review only - http://bmjopen.bmj.com/site/about/guidelines.xhtml

participants deposit their own money and are reimbursed only if they achieve the target weight loss, are effective whilst the incentives are in place.[4 23] Deposit contracts draw on loss aversion where people are more motivated to avoid losses than they are to achieve similarly sized gains.[24 25] However, deposit contracts may not be not equitable, as committing one's own money up front may not be possible, particularly for individuals with lower income.

Endowment incentive contracts may overcome limitations of deposit contracts. Endowment incentive contracts are financial incentives where a person is endowed with a (hypothetical) amount of money which they either 'secure' or 'lose' depending on achieving certain targets. A trial that framed financial incentives in this way found these incentives were effective for achieving PA goals in the short term.[26]

Aim

The aim of the Game of Stones feasibility trial was to examine the acceptability and feasibility of a men-only weight management intervention consisting of narrative text messages, with and without an endowment incentive, compared to waiting list control. The objectives were to:

1. Assess the acceptability and willingness to be randomised to: i) narrative text message and endowment incentives; ii) narrative text messages only; or iii) waiting list for text messages (control).
2. Assess the feasibility of recruiting from general practitioner (GP) practice obesity registers and community venues.
3. Determine the acceptability of intervention content, feasibility of delivery, fidelity and any unintended consequences.
4. Assess indicative effects on weight change and progression criteria for a full trial.

METHODS

Trial design

A three-arm individually randomised parallel-group controlled feasibility trial was conducted with an integrated qualitative and quantitative mixed methods approach[27]. Informed by MRC guidance on the evaluation of complex interventions, the study included an integrated mixed methods process evaluation[28 29]. Drawing on both qualitative and quantitative approaches allows the team to explore participants views (acceptability) of the intervention with

For peer review only - http://bmjopen.bmj.com/site/about/guidelines.xhtml

For peer review only

participants as well as explore implementation processes such as recruitment, retention and barriers and facilitators to these.

Participants were randomised to receive narrative text messages and endowment incentives (SMS+I), narrative text messages only (SMS only), or a waiting list for text messages (Control). The protocol the full study are available: https://www.journalslibrary.nihr.ac.uk/programmes/phr/1418509/#/

Ethical approval for the study was obtained from the North of Scotland Research Ethics Service (Ref: 16/NS/0120).

Setting

Two Health Board areas in Scotland (Sites A and B) with high levels of disadvantage were the setting for this study (see participant recruitment section for additional details).

Eligibility criteria

Participants met the following eligibility criteria: men over 18 years old; BMI ≥30kg/m^2 and/or a waist circumference of ≥40 inches (102 cm); owned a mobile phone capable of receiving text messages; could understand English language text messages; not already taking part in a weight loss study; not planning or waiting to have bariatric surgery; not planning to move within the next 12 months; considered by practice clinical staff as suitable for participation (GP practice recruitment only), for example, no severe medical, terminal or psychiatric illness (in patient or close family member) or no significantly impaired cognitive function.

Sample size

This study aimed to randomise 105 men, 35 to each arm, in line with recent recommendations for pilot trials as sufficient to estimate key parameters for a full trial.[30] A sample size of 35 per arm is sufficient to allow the population variance to be estimated (e.g. the SD in weight loss) with enough precision to deliver at least 80% power and 90% confidence in a full trial, with a standardised effect size between 0.2 and 0.5.[30]

Participant recruitment

Participants were recruited via: 1) community outreach, and 2) GP practice obesity registers.

For peer review only - http://bmjopen.bmj.com/site/about/guidelines.xhtml

Community outreach

Community recruitment strategies that report success for recruiting men in disadvantaged areas were used.[11-13] This included researchers working on stands with study information leaflets and table banners in supermarkets, fitness centres, hospital foyers, health centres, council workplaces and community centres across both sites. Eleven of the 13 recruitment venues were within more disadvantaged areas based on the Scottish Index of Multiple Deprivation (SIMD quintiles 1 and 2).[31] Men who showed interest were given study information and asked to leave their contact details with researchers.

In addition, some men were recruited through word of mouth. This included researcher discussions with family, friends, colleagues and local workers who passed on study information to eligible men they knew. Information leaflets were distributed across localities in shops, libraries, barbers and community centres. Interested men then contacted researchers to get more information.

GP practice obesity register letters

Practices within more disadvantaged areas (SIMD quintiles 1 or 2) were invited to participate in the study. Of the 33 practices invited (n= 13 in Site A, n=20 in Site B), five practices participated (n=4 in Site A, n=1 in Site B).

Practice database searches identified potentially eligible men with a documented BMI of at least 30kg/m^2. Lists were screened by clinical practice staff and details passed to the Health Informatics Centre at the University of Dundee who sent out GP-headed study invitation letters and information leaflets. Interested men contacted the research team or returned an 'opt-in' card.

Baseline Appointment

Men interested in participating were invited to attend a face-to-face appointment. Detailed study information was discussed and written informed consent provided. Anthropometric measurements were conducted and eligibility assessed. Participants then completed a baseline questionnaire, and were randomised. Group-specific post-randomisation information was provided and discussed with the participant.

For peer review only - http://bmjopen.bmj.com/site/about/guidelines.xhtml

Randomisation

At the baseline appointment, independent randomisation was performed by the researcher using the clinical trials unit's secure remote web-based system, stratifying by recruitment method (GP and community) and recruitment site (Site A and Site B).

Blinding

The two recruitment researchers who also assessed outcomes and conducted qualitative interviews were mostly not blinded to group allocations. All other study team members were blinded. A demonstration of the feasibility for researcher outcome assessors to be blind to group allocation was conducted with eleven participants at the six month assessment. In this case, a researcher who had not previously met the participant arranged and undertook the assessment. These participants all complied with the request not to reveal their group allocation.

The trial statistician was fully blinded to intervention groups, and partially blinded for the control group due to the different response schedule (control participants were only assessed at baseline months and 12 months to reflect "usual life").

Intervention components

Narrative text messages

A narrative text message library consisting of 604 texts was written by a professional scriptwriter/researcher (MG) who designed the overall narrative with enough interlinked stories to engage participants over 12 months. Full details of the narrative texts development process are available elsewhere.[10] Narrative texts were sent to participants over the course of 12 months and were written from the point of view of a fictional character aiming to lose weight over 12 months. Narrative texts were written to appeal to men from disadvantaged backgrounds. All participants in the SMS+I and SMS only groups received automated texts according to a pre-determined schedule. Participants could reply to texts but did not receive a response. Texts were sent between 8:00AM and 10:00PM and ranged from 0-5 texts per day. Some texts were personalised including participants' names and men could select whether weight information should be presented in kilogrammes or stones and pounds. No further personalisation and tailoring options were offered. Texts were delivered by the Health Information Centre in Dundee using existing automated technology linked to a clinical trials unit database.

For peer review only - http://bmjopen.bmj.com/site/about/guidelines.xhtml

Endowment incentive

The incentive strategy was informed by existing evidence and men's preferences elicited using a Discrete Choice Experiment completed by 1,045 men with obesity and reported elsewhere.[10] SMS+I participants were 'endowed' with a £400 incentive at baseline which was placed into a hypothetical personal account at the University of Stirling and given a mock-up personalised cheque. The full £400 could be secured by meeting weight loss targets at researcher assessments: 5% of body weight lost since baseline at three months (£50 secured/lost), 10% lost since baseline at six months (£150 secured/lost) and 10% lost since baseline at 12 months (£200 secured/lost) (online supplement 1). Weight loss was verified at all face-to-face appointments. At six and 12 months, men lost a proportion of the money for each % weight loss not attained between 5-10%. Weight at 12 months had to be less than at baseline to receive any money, regardless of whether interim weight loss targets had been met. Men received the money by direct bank transfer after the 12-month assessment. Feedback on meeting incentive targets was sent by automated text message and displayed on the personalised SMS+I webpage.

Website

All trial participants were provided with a unique login ID for the Game of Stones website. The front page was accessible to all participants and included trial information and links to existing evidence-based online weight management resources. SMS +I and SMS only webpages had a brief biography and images of the fictional characters featured in narrative texts. Participants could enter their weight, pedometer steps, waist circumference and belt notches, which were displayed as basic visual progress charts. Only individual performance was displayed on the webpage, and no group averages were shown for social comparison. SMS+I webpages described the financial incentives and a visual progress chart of money secured/lost.

Printed information

All participants received a weight loss fact sheet (British Dietetic Association, Weight Loss Food Fact Sheet) and a small card which could be carried in their wallet for noting website details and their appointment weight, weight loss targets and appointments.

Pedometer

All participants received a study pedometer (3DFitBud, A420S, manufactured by 3DActive).

For peer review only - http://bmjopen.bmj.com/site/about/guidelines.xhtml

Comparator group

At baseline, participants received access to the information section of the webpage, printed information (i.e. a weight loss fact sheet) and a pedometer. They attended a baseline and 12-month appointment only. Control group participants were offered texts for three months commencing after the 12 month data collection point.

Outcomes

The outcomes for this study related to whether the design of Game of Stones was both acceptable and feasible to deliver as a full scale randomised controlled trial. An independent study steering committee advised whether the following pre-specified progression criteria in the study protocol were met sufficiently to proceed to a full trial.

1. Acceptability of the intervention and the control group (by the majority of the target group); willingness to be randomised.
2. Feasibility of recruiting 105 men in four months.
3. 12-month outcomes on at least 72% of men randomised per group.
4. Evidence of mean weight loss of at least 3% of baseline weight at 12 months in any intervention group.
5. Commitment by, for example, government or NHS/local authorities to fund the incentive intervention to ensure translation and sustainability.

Outcome assessment

Outcomes were assessed at baseline, three, six and 12 months for intervention participants (SMS+I and SMS only), and at baseline and 12 months for control participants. Individual appointments were at community centres, universities, NHS clinical research facilities, voluntary sector organisations, GP practice premises, or the participant's home if no suitable alternative venue could be found. At the 12 month appointment, all participants received a £20 voucher as reimbursement for their time. No travel expenses were provided.

The text message delivery system automatically the frequency of responses received to the texts. The website automatically recorded engagement with self-monitoring tools.

Self-report questionnaires were completed during appointments and measured socio-demographics, co-morbidities, disability, ethnicity, and perceptions on intervention acceptability. Overall satisfaction with the intervention was assessed with the item: "*On a scale from 0 (not satisfied at all) to 100 (completely satisfied). How satisfied are you with the*

For peer review only - http://bmjopen.bmj.com/site/about/guidelines.xhtml

Game of Stones programme?". Acceptability of the intervention was assessed with the stem "*Overall the Game of Stones programme has been…*" followed by options '*understandable*', '*useful*', '*helpful*', '*interesting*' and '*relevant*', with responses captured on a 5-point scale ranging from '*totally disagree*' to '*totally agree*'. Helpfulness of the intervention was assessed with the item "*How helpful have you found the following in helping you lose weight?*" followed by options '*text messages*', '*website*', and '*pedometer*', with responses captured on a 5-point scale ranging from '*totally unhelpful*' to '*totally helpful*'. Measures of overall satisfaction, acceptability and helpfulness were adapted from Dombrowski et al., (2012).[32]

Anthropometry measures included height measured at baseline using a portable standing stadiometer (Seca 217, Birmingham, UK) to the nearest 0.1cm. Prior to weight measurements, participants removed shoes and bulky clothing and items from their pockets. Weight was recorded using portable calibrated electronic scales (Marsden M420, Rotherham, UK) to the nearest 0.01kg and waist circumference using a tape measure (Seca 203, Birmingham, UK) to the nearest 0.1cm.

Information on possible adverse events was recorded at assessment visits via open questions and through automatic monitoring of text message replies including words like 'suicide', 'die', and 'death' indicative of potential adverse events, which were notified to the research team by email.

Any negative participant reactions to their randomised group were recorded in researcher field notes to assess willingness to be randomised.

Interviews took place during three and 12 month appointments and were conducted face-to-face and detailed methods are described elsewhere.[10] All audio recorded interviews were transcribed verbatim and anonymised. At three months, all SMS+I and SMS only men were invited to participate in either a brief feedback or an in-depth interview (participant choice). The three month topic guide focussed on the early acceptability of the intervention components in order to identify any refinements required over the remaining nine month intervention. For the 12 month qualitative interviews, separate topic guides for the three trial groups were informed by the analysis of the three-month interviews, researcher field notes and to gain information power[33] to address the study objectives. Purposive sampling from the three trial groups was informed by the three month interview data and researcher field notes

For peer review only - http://bmjopen.bmj.com/site/about/guidelines.xhtml

to provide diversity of perspectives. Researcher field notes taken at all assessments were referred to when interpreting interview data.

Qualitative interviews were conducted face-to-face with 50 of 58 men who attended the three month assessment (7-61 minutes, median=23 minutes). At 12 months, interviews were conducted with 14 participants from SMS+I (13-65 minutes, median=33 minutes), 13 from SMS only (10-59 minutes, median=28 minutes) and six from the control group (7-18 minutes, median=11 minutes). Fourteen interviews at 12 months lasted <20 minutes, mostly for control participants who had received no contact from the research team between baseline and 12 months.

Analysis

Quantitative analysis

All continuous variables were summarised and tabulated using the following descriptive statistics: N (number of valid non-missing responses), mean, standard deviation (SD). Likert-scale variables were treated as continuous measures. The frequency and percentages (based on the non-missing sample size) of observed levels are reported for all categorical measures. The proportion of individuals contacted who were recruited and the proportions retained and withdrawn at each assessment by group was determined. Missing weight data are presented as baseline observation carried forward (BOCF) and last observation carried forward (LOCF) in addition to observed cases only.

CONSORT guidance for reporting randomised pilot and feasibility studies was followed[34].

Qualitative and mixed method data analysis

Anonymised transcripts of interviews together with: attributes for recruitment channel; attendance at assessments, trial group and participant characteristics and weight loss outcomes were entered into NVivo12 software for analysis guided by the framework approach.[35] Reference to researcher field notes contributed to interview data interpretation. Iterative data collection and analysis were driven by the key feasibility and acceptability research questions and objectives. A coding frame was developed by three researchers independently reading a diverse sample of six interviews, followed by a team discussion to finalise the coding frame and identify key themes. Independent coding was conducted by four researchers (EC, NG, MM and RS) and checked for consistency by FH. Two independent researchers (EC and NG) who were not involved in any other aspect of the study, assisted with coding and analysis to enhance rigour and reliability of data analysis. Emergent themes

For peer review only - http://bmjopen.bmj.com/site/about/guidelines.xhtml

and interpretive analysis were discussed at weekly researcher meetings and at two Co-Investigator qualitative interpretation meetings.

At the final stage of mixed methods data interpretation, the quantitative attributes on NVivo were drawn on to triangulate the analysis and suggest further avenues for interrogation. Matrix coding queries in NVivo were generated to cross-reference attributes for trial group, participant SIMD and weight loss outcome at 12 months, with nodes coded for views of incentives and texts messages respectively. Credibility and reliability of qualitative analysis was enhanced by independent coding, use of memos to ensure transparency of interpretation, and interpretive charting conducted with input from the wider study team.

Patient and Public Involvement

A continuous and responsive approach to patient and public involvement (PPI) was adopted to prepare the grant application and throughout the study, as described by Gamble and colleagues.[36]

Continuous PPI was provided by a Co-Investigator partnership with the Charities Men's Health Forum GB and Men's Health Forum in Ireland. The partnership commenced in 2011 with the ROMEO evidence syntheses of weight loss interventions for men with obesity.[4]

During the study, Co-Investigators from each of the Men's Health Forum charities attended trial management meetings to contribute to decisions, intervention development, data analysis, interpretation of findings and reporting. They provided feedback on the grant application, protocol, text messages, information materials and engaged wider involvement of men from their organisation to assist with appropriate language.[37]

Continuous PPI at the study oversight level was provided by two independent lay members of the study steering committee which met on three occasions.

Responsive PPI occurred at the study funding application stage. Co-Investigator CG engaged men who provided PPI input in the FFIT Trial.[38] During the study, relevant members of the public were identified through several sources including: Men's Health Forum GB, Men's Health Forum in Ireland, Scottish Community Health Councils, Men's Sheds, University of Stirling PPI group, Alliance Scotland and other Co-Investigator contacts. Effort was made to engage men from more disadvantaged communities. A total of 121 PPI contributors were involved in a range of study activities, including finding a study name, helping with wording of information material, and helping with designing the intervention content. For full details see the report to the funder.[10]

For peer review only - http://bmjopen.bmj.com/site/about/guidelines.xhtml

RESULTS

Recruitment and retention

Recruitment was completed in four months between March to June 2017 meeting the target number of 105 men randomised (Figure 1).

Overall, 177 men expressed an interest in the study. Baseline appointments were attended by 111 (63%), of which six (5%) were ineligible. All 105 men consented to be randomised to the SMS+I (n=36), SMS only (n=33) and control (n=36) groups.

GP practice recruitment was undertaken in five GP practices (Site A n=4, Site B n=1) with 45 men recruited and randomised through GP practices. The additional 60 men were recruited through community outreach.

--- Figure 1 ---

The 12 month assessment was completed by 79/105 participants (75%). One of the 79 participants (control group) could not attend and provided self-reported information because he was out of the region due to work commitments, which means that 74% (78/105) of participants were retained for weight assessments at 12 months. Overall 12 month retention differed by group (SMS+I=23/36, 64%; SMS only=26/33, 79%; control=30/36, 83%). Fourteen participants (13%) were lost to follow-up, and 12 participants (11%) withdrew. Reasons for withdrawal were dislike of narrative texts (n=4), health (n=3), family (n=1), unknown (n=1), dissatisfaction with group allocation (n=1), appointment logistics (n=1) and multiple reasons (n=1).

Baseline characteristics

Baseline characteristics are displayed in Table 1. On average, participants were 52.2 (SD=13.1) years old, had a BMI of 35.7 (SD=5.9) and a waist circumference of 116.8 cm (SD=11.8). BMI ranged from 27.5 kg/m^2 to 62.5kg/m^2. Twelve participants (11%) had a baseline BMI less than 30 kg/m^2 due to the additional weight circumference entry criterion of ≥40 inches (102 cm).

The majority of men lived in disadvantaged areas defined as SIMD quintiles 1 or 2 (n=62/104; 60%). Most participants were married (58%), reported at least one co-morbidity (63%), were of white ethnicity (91%), reported having children (76%), and were in full-time employment (48%).

--- Table 1 ---

For peer review only - http://bmjopen.bmj.com/site/about/guidelines.xhtml

For peer review only

Intervention fidelity

The intervention components were feasible to deliver with fidelity. The majority (95.4%) of the 38,214 text messages sent to the two intervention groups were delivered to the mobile phone, with 1,782 (4.6%) having no delivery status (i.e. lack of response from the mobile phone provider after 48 hrs and invalid phone numbers). No major technical errors occurred. All participants who secured financial incentives were paid by direct bank transfer, in line with their stated preference.

Acceptability of intervention components

Overall acceptability ratings and contamination

Table 2 displays intervention satisfaction indicators over the course of the study for participants attending assessments. Overall mean study satisfaction at 12 months was 81%, 77% and 87% for the SMS+I, SMS only and control groups respectively. Satisfaction over time was comparable between both intervention groups. For scores on specific programme attributes see Table 2.

Helpfulness ratings of the narrative texts, webpage and pedometer were highest for the pedometer and relatively stable throughout the measurement time points. At 12 months, helpfulness ratings (out of 5) for the pedometer were 3.9, 4.0, and 3.7 for the SMS+I, SMS only and control groups respectively. The narrative texts (SMS+I=3.4; SMS only=3.3) and the webpage (SMS+I=3.6; SMS only=3.4; control=3.5) were perceived as somewhat helpful on average at 12 months.

Minimal contamination between intervention groups was observed, with one participant at six and 12 months (SMS+I group) and another at 12 months (control group) reporting meeting other men in the study.

--- Table 2 ---

Acceptability of narrative text messages

Some men sent spontaneous replies to the narrative texts (0-3 months n=25/69, 36%; 3-6 months n=8/69, 12%, 6-12 months n=13/69, 19%; see online supplement 2). Most spontaneous replies were received between 0-3 months (n=370 replies) and decreased at 3-6 months (n=16 replies) and 6-12 months (n=39 replies).

Eleven participants (11/69, 16%) requested to no longer receive texts but asked to remain in the trial. Requests to stop narrative texts were similar in both groups (SMS+I=5/36, 14%;

For peer review only - http://bmjopen.bmj.com/site/about/guidelines.xhtml

SMS only=6/33, 18%), and occurred throughout the study. Four participants (4/69, 6%) withdrew due to dislike of narrative texts between baseline and three months, two in each intervention group.

Qualitative interviews at three and 12 months demonstrated varied views on the narrative texts. Participants' views ranged from positive to indifferent to negative. Those who liked the narrative texts found the storyline entertaining, engaging and some participants felt a certain camaraderie for the main character:

> *You get involved in the content and you start following the script, they're very funny and me being a [from city name] you can see the funny side and you can see the wit in that, ... and that's what keeps you reading them*. (210010, SMS only, 12m)

For other participants, the frequency of texts became a source of irritation, the storyline did not resonate with their own experience nor could they empathise with the fictional characters:

> *I'm not a lover of chocolate, biscuits, cake, ice cream, I hardly ever touch them, hardly ever, so hearing about somebody eating pizza or sweets means nothing to me because I don't eat them anyway* (120002, SMS+I, 12m)

Some were uncertain of the role of the texts in helping to support weight loss. Others expressed an indifference towards the texts, and a decreasing interest in the story over time leading to infrequent engagement with text content. However, the regular reminder of being a participant in a weight loss programme through receiving texts was seen as important:

> *I often wonder, do they relate to myself here. And the only commonality is both trying to lose weight. So in that respect it's just a reminder all the time, you should be reviewing your weight and watching what you're eating, so…from that it's a positive, yeah.* (220017, SMS+I, 3m)

A matrix coding query and analysis of the quotations generated from the NVivo qualitative and demographic data suggested no obvious relationships between the participant SIMD level and the qualitative accounts of acceptability of the narrative texts. This is in line with quantitative data showing similar acceptability ratings for overall programme satisfaction and helpfulness ratings of the narrative texts across SIMD levels (see online supplement 3). Some men would have liked more interactivity built into the texts, however, the text message system was set up for uni-directional messaging only.

For peer review only - http://bmjopen.bmj.com/site/about/guidelines.xhtml

Acceptability of endowment incentive

At three months, 31/36 SMS+I participants attended the appointment, with eight men losing 5% or more of their baseline weight and securing £50. Attendance at six months was lower (n=23/36), with five participants achieving the weight loss target of >10% weight loss securing £150 each, and a further three participants losing between ≥5-10% of weight securing between £75-150. At 12 months, 23/36 participants attended the appointment, with three achieving >10% weight loss securing £200, and a further seven losing between ≥5-10% of weight securing between £100-200.

Overall, £2,955 was paid in total to the 11 participants (11/36, 31%) successfully meeting or partially meeting weight loss targets. The full £400 was secured by three participants (3/36, 8%). The cost of the incentive per participant was £81.94 (95% CI £34.59-£129.30). All participants who completed the study and secured money at three or six months received it at the end of the study. No participant lost previously secured money due to weight regain to baseline weight at 12 months. One participant secured money at an early appointment but withdrew from the study, did not attend at 12 months, and did not receive any money.

No negative views were expressed about the incentives in qualitative interviews. Only one man reported being motivated by the financial incentive, and many expressed indifference. Participants reported the weight loss and health benefits as sufficiently rewarding.

> *The money's not that much of an issue. I think the incentive for me is the health that, at the end of the day, your health improves. That's the most important incentive. The money, it's fine, it's there, but it's not the incentive* (120022, SMS+I, 3m)

Some interpreted the incentives as a final reward linked to their weight loss targets, whereas others fully understood the intended use of the loss aversion concept:

> [W]e *all know how much more distress it causes you to lose money than the pleasure of finding a fiver down the back of the couch, you know, so I think if you lose something it almost has a bigger impact on you than if you gain*. (220040, SMS+I, 3m)

Acceptability of study webpages and pedometer

Some participants used the self-monitoring features on the webpage (see online appendix 3) and reported valuing the ability to visually track their weight in interviews. However, most

For peer review only - http://bmjopen.bmj.com/site/about/guidelines.xhtml

did not access the website. The majority (77%) did not own a pedometer at baseline, and pedometers were highly acceptable to participants.

Acceptability of Control Group

Most control group participants (30/36, 83%) attended the 12 month appointment. Six qualitative interviews were conducted with men in the control group covering topics including acceptability of waiting 12 months for text messages, views about the study and improvement suggestions. Many reported being pleased to be involved in a research study. One participant had a strong negative reaction to being randomised to the control group and subsequently withdrew when invited to the 12 month assessment.

Weight outcomes

Table 3 displays weight change outcomes at three, six, and 12 months. The difference in mean percentage weight loss at 12 months for observed cases was −2.51% (95% CI −6.03 to 1.01) for SMS+I v control and −0.51% (95% CI −3.91 to 2.89) for SMS only v control. The difference in absolute weight loss at 12 months was −2.87kg (95% CI −6.82 to 1.08) for SMS+I v control and −0.57kg (95% CI −4.40 to 3.25) for SMS only v control.

---Table 3 ---

The SMS+I group displayed mean weight loss of over 3% at 12 months (-3.51%, SD=5.83). The SMS only and control groups remained below 3% weight loss on average (SMS only=-1.51%, 4.65; control=-1.00%, SD=5.31). The highest mean percentage weight loss at 12 months for BOCF was -2.24% (SD=4.93) in the SMS+I group, followed by -1.19% (SD=4.16) and -0.80% (SD=4.77) in the SMS only and control groups respectively.

Harms and unintended consequences

No harms or unintended consequences were reported.

Progression to full trial

An independent study steering committee agreed that the Game of Stones study had demonstrated acceptability and feasibility agreeing that overall the pre-specified progression criteria were sufficiently met to support a full three-arm multi-site RCT.

For peer review only - http://bmjopen.bmj.com/site/about/guidelines.xhtml

DISCUSSION

Principal findings

This study successfully recruited 105 men from across the socioeconomic spectrum to a three-armed RCT and overall achieved 74% retention at 12 months' follow-up. Men living in more disadvantaged areas formed 60% of the sample. Narrative texts were broadly acceptable, but some participants disengaged or withdrew from the study due to dislike of texts. The endowment incentives were acceptable, and improving health was reported as a key motivator for weight loss. Positive indicative effects of weight loss were found in intervention and control groups.

Strengths and weaknesses of the study

This study was underpinned by the ROMEO systematic reviews and qualitative evidence synthesis of weight loss interventions for men with obesity[39 40], with a continuing PPI partnership with the Men's Health Forum GB and Ireland charities. Mixed methods research combined quantitative and qualitative data which, together with patient, public and stakeholder involvement, provided a multi-lens perspective on this study.

Most assessments were not blind to group allocation. The difference in assessment schedules for intervention and control groups meant that study staff were only partially blind. A full trial should ensure full blinding of all outcome assessments. Researchers were unable to collect data via qualitative interviews, questionnaires and anthropometric measures with men who withdrew from the study or were lost to follow-up at the 12 month appointment. Those providing a withdrawal reason may have provided socially desirable responses. Text messages were personalised by including the participant's name and weight unit preference, but no tailoring or interactivity were possible due to technical limitations of the delivery system. This feasibility trial was not powered to detect effects on weight loss and, in line with recommendations[41], no p-values are reported. Weight outcomes should be interpreted with caution.

Relation to other studies

The Game of Stones study adds to the evidence base demonstrating the feasibility of recruiting men for research on sensitive subjects, such as obesity, through community outreach and GP practice lists.[11-13]

For peer review only - http://bmjopen.bmj.com/site/about/guidelines.xhtml

The average BMI and age of the study participants of around 35kg/m^2 and 50-55 years is similar to UK weight management trials recruiting in the community[38], primary care[42], or a combination of community and primary care.[12] Three international text message based weight management studies examining outcomes after 12 months recruited younger participants with lower BMI.[43-45] However, three mixed sex weight management studies with financial incentives (two including text message components) reported broadly similar participant demographics to this study.[46-48]

Retention levels of 74% were acceptable and are similar to systematic review evidence of men-only weight loss interventions which found an average retention of 78%, ranging from 44% to 100%.[40] International text message studies with mixed sex participants report similar retention rates at 12 months of 70.3%[44] and 73%[43], with one study conducted in Latvia reporting 93% retention[45]. Two large UK studies recruiting mixed sex participants with obesity to weight management in primary care reported 75%[42] and 81%[49] retention at 12 months.

Two previous narrative text message intervention studies in men targeting alcohol reduction reported high acceptability levels and no negative reactions.[12 13] However, these studies were only 12 weeks in duration and overall contained fewer texts. The narrative texts in this study were broadly acceptable, although some negative reactions were reported. Study withdrawal due to texts and requests to stop texts suggest that the current narrative texts may not be universally acceptable to men. The text messages were designed by a professional scriptwriter/researcher with PPI input from the target population of men from disadvantaged backgrounds, but the narrative texts were similarly acceptable across the socio-economic spectrum. Intervention fidelity was high with the majority of sent texts successfully delivered and no adverse events were encountered, similar to previous text message-based intervention studies.[11-13]

The use of a financial incentive strategy for weight loss in men with obesity across the socioeconomic spectrum was feasible and acceptable. Two previous incentive studies used text messages to inform participants about their incentive achievement of weight loss targets, and found it acceptable.[46 47] This extends the evidence base on the use of financial incentives as a complementary behaviour change strategy alongside other components.[22 50]

The financial incentive strategy was designed with future sustainability in mind and to be attractive to public sector funders. The text messages and incentive components are mostly

For peer review only - http://bmjopen.bmj.com/site/about/guidelines.xhtml

automated, encourage self-management of weight and have a low administrative burden with one bank transfer pay out after 12 months, and verification of weight loss at three, six and 12 months. Yancy et al. similarly provided incentives at three and six months for their six months' incentive intervention[46]. John et al. asked participants to be weighed monthly, for which they received $20 per visit.[47] Few RCTs examine financial incentive strategies that are delivered for at least 12 months.[50] Two other studies providing financial incentives for 12 months provided an intensive financial incentive schedule either weekly[51] or monthly[52]. Previous studies typically provided participants with weekly weight loss goals such as 1lb (0.45kg) per week, on which financial incentives were contingent.[50] Whilst these goals are similar to the weight loss targets in the current study, there were no intermittent or weekly targets. A balance needs to be struck between having more regular weight measurements and more immediate pay-outs, costs and future sustainability.

Patel et al. applied a similar framing which they called a loss incentive[26]. University employees were allocated a monthly hypothetical incentive of $42 upfront and $1.40 was taken away each time their daily goal of 7000 steps was not met. The current study adds to the evidence base demonstrating the acceptability and feasibility of loss framed interventions for weight loss and in settings outside workplaces.

Conclusion

This three-arm weight management feasibility trial recruited men from across the socioeconomic spectrum with the majority coming from disadvantaged areas, had an acceptable retention rate, and was broadly acceptable to most participants, and feasible to deliver. A full trial of the study is warranted.

For peer review only

ACKNOWLEDGEMENT

We would like to acknowledge the following people who have contributed to this study:

All men who contributed to PPI and who participated in the study.

Colin Fowler from Men's Health Forum in Ireland, who was involved in the ROMEO systematic review that informed this study, commented on the grant application, protocol and final report. Together with Paula Carroll, they accessed wider PPI in Ireland.

Fiona Hogarth, Director Tayside Clinical Trials Unit, oversaw randomisation, data management and the running of the study from a Clinical Trials Unit perspective.

Hannah Collacott, who was employed as a research assistant at the Health Economics Research Unit, University of Aberdeen in 2016-2017. She undertook the think aloud interviews, PPI and assisted in the design and preliminary analysis of the DCE described in the final report to the funder.(Dombrowski et al., in submission)

Clinical, Community and Academic Networks who advised our team on many aspects of this study, including: NHS Research Scotland Primary Care Network and the GP practices involved, Alliance Scotland, Community Health Councils and Health Centres

The following post-graduate Masters students, who accompanied the research assistants when recruiting men in the community in alphabetical order: Norelle Calder-Macphee, Ines Elenin Lucy Gunatillake, Bryony Lang, Heather Renwick and Amy Spiteri.

Karen Stanton, Lorna Kerr, Karen Murray, Siobhan McDermott, Suzannah Hunter, who provided administrative support.

The prior work of Professor Crombie and his team at Dundee who developed the software to deliver the SMS and assisted with the grant application for this study.(Crombie et al., 2017; Crombie et al., 2018; Irvine et al., 2017)

The Study Steering Committee:

Associate Professor Ed Juszczak, Director, NPEU Clinical Trials Unit, Nuffield Department of Population Health, University of Oxford (Chair).

Professor Kate Jolly, Professor of Public Health & Primary Care, University of Birmingham.

Mr. Mark Kelvin, Programme Director (until 2018), Health and Social Care Alliance Scotland (the ALLIANCE).

For peer review only - http://bmjopen.bmj.com/site/about/guidelines.xhtml

Mr. Andrew Shanahan, Director MAN v FAT - the weight loss website for men

Ms. Martine Stead, Deputy Director, Institute for Social Marketing, University of Stirling

Miss Joyce Thompson Dietetic Consultant in Public Health Nutrition and Tayside Nutrition MCN Lead Clinician, NHS Tayside.

AUTHOR CONTRIBUTIONS

SD co-led the study with PH as joint Chief-Investigator and co-led the design and overall conduct of the study and led the writing of the manuscript, overseen by PH.

MM undertook the study set up, recruitment and data collection, co-ordinated various PPI activities, contributed substantially to qualitative and quantitative data analysis and interpretation. MvdP was a co-investigator, contributed to the design and the interpretation of findings and led the DCE and health economic analysis. MG was a co-investigator, contributed to the design and the interpretation of findings and led the development of the narrative SMS component, the related PPI. AA was a co-investigator, contributed to the design and the interpretation of findings. PC and MT linked to PPI networks, contributed to the design, and interpretation of the findings. AE was a co-investigator, contributed to the design and the interpretation of findings and oversaw the statistical analysis. NG and EC contributed to the analysis and interpretation of the qualitative data. CG was a co-investigator, facilitated PPI, contributed to the design, and interpretation of findings. FH was a co-investigator, oversaw the qualitative data collection and analysis and contributed to the interpretation of findings. AH undertook the statistical analysis at Tayside Clinical Trials Unit. CJ was a co-investigator, contributed to the design and the interpretation of findings and oversaw the automated SMS delivery system, recruitment tracking, the study website and integration of data with databases at Tayside Clinical Trials Unit. FK and MMcK were co-investigators and contributed to the design and the interpretation of findings. RS contributed to recruitment, data collection and undertaking qualitative interviews and contributed to the interpretation of findings.

FUNDING

This report presents independent research commissioned by the NIHR. The views and opinions expressed by authors in this publication are those of the authors and do not necessarily reflect those of the NHS, the NIHR, MRC, CCF, NETSCC, the Public Health Research programme or the Department of Health. The views and opinions expressed by the interviewees in this publication are those of the interviewees and do not necessarily reflect

For peer review only - http://bmjopen.bmj.com/site/about/guidelines.xhtml

those of the authors, those of the NHS, the NIHR, MRC, CCF, NETSCC, the Public Health Research programme or the Department of Health.

The Nursing Midwifery and Allied Health Professionals Research Unit, the Health Services Research Unit and Health Economics Research Unit are funded by the Chief Scientist Office of the Scottish Government Health and Social Care Directorate.

COMPETING INTERESTS

Dr Grindle is Director of Eos Digital Health Ltd which holds all rights to DNA - The Digital Narrative Approach deployed to design and write the narrative text intervention.

No other competing interests were declared.

DATA AVAILABILITY

Access to data can be arranged through the Co-PIs of the study: Prof. Pat Hoddinott, (University of Stirling, p.m.hoddinott@stir.ac.uk) and Dr. Stephan Dombrowski (University of New Brunswick, stephan.dombrowski@unb.ca) to discuss data sharing, data requirements and conflicts of interest, in line with any EU and other regulations, including ethics approvals.

For peer review only

REFERENCES

1. Ng M, Fleming T, Robinson M, et al. Global, regional, and national prevalence of overweight and obesity in children and adults during 1980–2013: a systematic analysis for the Global Burden of Disease Study 2013. *The Lancet* 2014;384(9945):766-81. doi: 10.1016/S0140-6736(14)60460-8

2. The Scottish Government. The Scottish Health Survey 2016. Edinburgh, 2017.

3. Health Survey for England. http://www.hscic.gov.uk/public-health. 2014

4. Robertson C, Archibald D, Avenell A, et al. Systematic reviews of and integrated report on the quantitative, qualitative and economic evidence base for the management of obesity in men. *HTA* 2014;18(35):1-424. doi: 10.3310/hta18350

5. Hall AK, Cole-Lewis H, Bernhardt JM. Mobile Text Messaging for Health: A Systematic Review of Reviews. *Annual Review of Public Health* 2015;36(1):393-415. doi: doi:10.1146/annurev-publhealth-031914-122855

6. Shaw R, Bosworth H. Short message service (SMS) text messaging as an intervention medium for weight loss: A literature review. *Health Informatics Journal* 2012;18(4):235-50. doi: 10.1177/1460458212442422

7. Palmer M, Sutherland J, Barnard S, et al. The effectiveness of smoking cessation, physical activity/diet and alcohol reduction interventions delivered by mobile phones for the prevention of non-communicable diseases: A systematic review of randomised controlled trials. *PLoS ONE* 2018;13(1):e0189801. doi: 10.1371/journal.pone.0189801

8. Ofcom. Adults' Media Use and Attitudes Report. 2016

9. Hinyard LJ, Kreuter MWJHE, Behavior. Using narrative communication as a tool for health behavior change: a conceptual, theoretical, and empirical overview. 2007;34(5):777-92.

10. Dombrowski SU, McDonald M, van der Pol M, et al. Feasibility trial of how to engage men with obesity in SMS (short message system) and incentive interventions for weight loss. in submission

For peer review only

11. Crombie IK, Irvine L, Falconer DW, et al. Alcohol and disadvantaged men: A feasibility trial of an intervention delivered by mobile phone. *Drug and Alcohol Review* 2017;36(4):468-76. doi: 10.1111/dar.12455

12. Irvine L, Crombie IK, Cunningham KB, et al. Modifying alcohol consumption to reduce obesity: A randomized controlled feasibility study of a complex community-based intervention for men. *Alcohol and Alcoholism* 2017;52(6):677-84. doi: 10.1093/alcalc/agx067

13. Crombie IK, Irvine L, Williams B, et al. Texting to Reduce Alcohol Misuse (TRAM): main findings from a randomized controlled trial of a text message intervention to reduce binge drinking among disadvantaged men. *Addiction* 2018;13(9):1609–18. doi: 10.1111/add.14229

14. Haff N, Patel MS, Lim R, et al. The role of behavioral economic incentive design and demographic characteristics in financial incentive-based approaches to changing health behaviors: A meta-analysis. *Am J Health Promot* 2015;29(5):314-23. doi: 10.4278/ajhp.140714-LIT-333

15. Mantzari E, Vogt F, Shemilt I, et al. Personal financial incentives for changing habitual health-related behaviors: A systematic review and meta-analysis. *Preventive Medicine* 2015;75:75-85. doi: 10.1016/j.ypmed.2015.03.001

16. Jay M, Orstad SL, Wali S, et al. Goal-directed versus outcome-based financial incentives for weight loss among low-income patients with obesity: Rationale and design of the Financial Incentives for Weight Reduction (FIReWoRk) randomised controlled trial. *BMJ Open* 2019;9(4) doi: 10.1136/bmjopen-2018-025278

17. Leahey TM, LaRose JG, Mitchell MS, et al. Small Incentives Improve Weight Loss in Women From Disadvantaged Backgrounds. *American Journal of Preventive Medicine* 2018;54(3):e41-e47. doi: 10.1016/j.amepre.2017.11.007

18. Ryan M, Yi D, Avenell A, et al. Gaining pounds by losing pounds: preferences for lifestyle interventions to reduce obesity. *Health Economics, Policy and Law* 2014;10(2):161-82 doi: 10.1017/S1744133114000413

19. Burns RJ, Donovan AS, Ackermann RT, et al. A theoretically grounded systematic review of material incentives for weight loss: Implications for interventions. *Annals of Behavioral Medicine* 2012;44(3):375-88. doi: 10.1007/s12160-012-9403-4

For peer review only - http://bmjopen.bmj.com/site/about/guidelines.xhtml

20. Giles EL, Robalino S, McColl E, et al. The effectiveness of financial incentives for health behaviour change: Systematic review and meta-analysis. *PLoS ONE* 2014;9(3):e90347. doi: 10.1371/journal.pone.0090347

21. Purnell JQ, Gernes R, Stein R, et al. A systematic review of financial incentives for dietary behavior change. *Journal of the Academy of Nutrition and Dietetics* 2014;114(7):1023-35. doi: 10.1016/j.jand.2014.03.011

22. Ananthapavan J, Peterson A, Sacks G. Paying people to lose weight: the effectiveness of financial incentives provided by health insurers for the prevention and management of overweight and obesity – a systematic review. *Obesity Reviews* 2018;19(5):605-13. doi: 10.1111/obr.12657

23. Paloyo AR, Reichert AR, Reinermann H, et al. The causal link between financial incentives and weight loss: an evidence-based survey of the literature. *Journal of Economic Surveys* 2014;28(3):401-20. doi: 10.1111/joes.12010

24. Camerer CF, Loewenstein G, Rabin M. Advances in Behavioral Economics: Princeton University Press 2003.

25. Cawley J, Downs JS, Loewenstein G. Behavioral Economics and Obesity: Oxford University Press 2012.

26. Patel MS, Asch Md DA, Rosin R, et al. Framing financial incentives to increase physical activity among overweight and obese adults a randomized, controlled trial. *Annals of Internal Medicine* 2016;164(6):385-94. doi: 10.7326/M15-1635

27. O'Cathain A, Murphy E, Nicholl J. Three techniques for integrating data in mixed methods studies. *Bmj* 2010;341:c4587. doi: 10.1136/bmj.c4587 [published Online First: 2010/09/21]

28. Craig P, Dieppe P, Macintyre S, et al. Developing and evaluating complex interventions: the new Medical Research Council guidance. *Bmj* 2008;337:a1655. doi: 10.1136/bmj.a1655 [published Online First: 2008/10/01]

29. Moore GF, Audrey S, Barker M, et al. Process evaluation of complex interventions: Medical Research Council guidance. *Bmj* 2015;350:h1258. doi: 10.1136/bmj.h1258 [published Online First: 2015/03/21]

For peer review only

30. Teare M, Dimairo M, Shephard N, et al. Sample size requirements to estimate key design parameters from external pilot randomised controlled trials: a simulation study. *Trials* 2014;15(1):264. doi: 10.1186/1745-6215-15-264

31. The Scottish Government. Definition of SIMD quintiles. Edinburgh, 2013.

32. Dombrowski SU, Sniehotta FF, Johnston M, et al. Optimizing acceptability and feasibility of an evidence-based behavioral intervention for obese adults with obesity-related co-morbidities or additional risk factors for co-morbidities: An open-pilot intervention study in secondary care. *Patient Educ Couns* 2012;87(1):108-19. doi: 10.1016/j.pec.2011.08.003

33. Malterud K, Siersma VD, Guassora AD. Sample Size in Qualitative Interview Studies: Guided by Information Power. *Qualitative Health Research* 2016;26(13):1753-60. doi: 10.1177/1049732315617444

34. Eldridge SM, Chan CL, Campbell MJ, et al. CONSORT 2010 statement: extension to randomised pilot and feasibility trials. *BMJ* 2016;355:i5239. doi: 10.1136/bmj.i5239

35. Ritchie J, Spencer L. Qualitative Data Analysis for Applied Policy Research: SAGE 2002.

36. Gamble C, Dudley L, Allam A, et al. Health Services and Delivery Research. An evidence base to optimise methods for involving patient and public contributors in clinical trials: a mixed-methods study. Southampton (UK): NIHR Journals Library 2015.

37. Men's Health Forum. Mind your language: How men talk about mental health. London2018.

38. Hunt K, Wyke S, Gray CM, et al. A gender-sensitised weight loss and healthy living programme for overweight and obese men delivered by Scottish Premier League football clubs (FFIT): A pragmatic randomised controlled trial. *The Lancet* 2014;383(9924):1211-21. doi: 10.1016/S0140-6736(13)62420-4

39. Archibald D, Douglas F, Hoddinott P, et al. A qualitative evidence synthesis on the management of male obesity. *BMJ Open* 2015;5(10):e008372. doi: 10.1136/bmjopen-2015-008372

40. Robertson C, Avenell A, Stewart F, et al. Clinical Effectiveness of Weight Loss and Weight Maintenance Interventions for Men: A Systematic Review of Men-Only

For peer review only

Randomized Controlled Trials (The ROMEO Project). *Am J Mens Health* 2017;11(4):1096-123. doi: 10.1177/1557988315587550

41. Eldridge SM, Chan CL, Campbell MJ, et al. CONSORT 2010 statement: extension to randomised pilot and feasibility trials. 2016;355 doi: 10.1136/bmj.i5239 %J BMJ

42. Aveyard P, Lewis A, Tearne S, et al. Screening and brief intervention for obesity in primary care: a parallel, two-arm, randomised trial. *The Lancet* 2016;388(10059):2492-500. doi: 10.1016/S0140-6736(16)31893-1

43. Haapala I, Barengo NC, Biggs S, et al. Weight loss by mobile phone: A 1-year effectiveness study. *Public Health Nutrition* 2009;12(12):2382-91. doi: 10.1017/S1368980009005230

44. Shapiro JR, Koro T, Doran N, et al. Text4Diet: A randomized controlled study using text messaging for weight loss behaviors. *Preventive Medicine* 2012;55(5):412-17. doi: 10.1016/j.ypmed.2012.08.011

45. Silina V, Tessma MK, Senkane S, et al. Text messaging (SMS) as a tool to facilitate weight loss and prevent metabolic deterioration in clinically healthy overweight and obese subjects: a randomised controlled trial. *Scandinavian Journal of Primary Health Care* 2017;35(3):262-70. doi: 10.1080/02813432.2017.1358435

46. Yancy Jr WS, Shaw PA, Wesby L, et al. Financial incentive strategies for maintenance of weight loss: results from an internet-based randomized controlled trial. *Nutrition & Diabetes* 2018;8(1):33. doi: 10.1038/s41387-018-0036-y

47. John LK, Loewenstein G, Troxel AB, et al. Financial incentives for extended weight loss: A randomized, controlled trial. *Journal of General Internal Medicine* 2011;26(6):621-26. doi: 10.1007/s11606-010-1628-y

48. Leahey TM, Subak LL, Fava J, et al. Benefits of adding small financial incentives or optional group meetings to a web-based statewide obesity initiative. *Obesity* 2015;23(1):70-76. doi: 10.1002/oby.20937

49. Little P, Stuart B, Hobbs FR, et al. An internet-based intervention with brief nurse support to manage obesity in primary care (POWeR+): a pragmatic, parallel-group, randomised controlled trial. *Lancet Diabetes Endocrinol* 2016;4(10):821-28. doi: 10.1016/S2213-8587(16)30099-7

For peer review only - http://bmjopen.bmj.com/site/about/guidelines.xhtml

50. Paul-Ebhohimhen V, Avenell A. Systematic review of the use of financial incentives in treatments for obesity and overweight. *Obesity Reviews* 2008;9(4):355-67. doi: 10.1111/j.1467-789X.2007.00409.x

51. Jeffery RW, Wing RR, Thorson C, et al. Strengthening Behavioral Interventions for Weight Loss: A Randomized Trial of Food Provision and Monetary Incentives. *Journal of Consulting and Clinical Psychology* 1993;61(6):1038-45. doi: 10.1037/0022-006X.61.6.1038

52. Kramer FM, Jeffery RW, Snell MK, et al. Maintenance of successful weight loss over 1 year: Effects of financial contracts for weight maintenance or participation in skills training. *Behavior Therapy* 1986;17(3):295-301. doi: 10.1016/S0005-7894(86)80058-2

For peer review only

Table 1 Participant baseline characteristics

	SMS + I N=36	SMS only N=33	Control N=36	Total N=105
Age (years), mean (SD)	50.9 (14.2)[a]	52.5 (15.1)[b]	53.1 (10.1)	52.2 (13.1)[c]
Waist circumference (cm), mean SD	115.8 (10.0)	114.9 (12.7)	119.5 (12.2)	116.8 (11.8)
Weight (kg), mean (SD)	108.6 (16.4)	107.8 (20.2)	110.7 (19.0)	109.1 (18.4)
Height (cm), mean (SD)	175.9 (6.6)	175.2 (6.7)	173.8 (5.9)	175.0 (6.4)
BMI (kg/m^2), mean (SD)	35.1 (5.3)	35.1 (5.9)	36.7 (6.5)	35.7 (5.9)
**BMI (kg/m^2) categories, n (%)**				
<30	5 (13.9)	3 (9.1)	3 (8.3)	11 (10.5)
≥30->35	16 (44.4)	19 (57.6)	14 (38.9)	49 (46.7)
≥35->40	10 (27.8)	6 (18.2)	7 (19.4)	23 (21.8)
≥40->45	4 (11.1)	2 (6.1)	10 (27.8)	16 (15.2)
≥45->50	0 (0.0)	2 (6.1)	1 (2. 8)	3 (2.9)
≥50	1 (2.8)	1 (3.0)	1 (2. 8)	3 (2.9)
**SIMD deprivation category, n (%)**				
SIMD 1 (most disadvantaged)	11 (31.4)[a]	12 (36.4)	15 (41.7)	38 (36.5)[d]
SIMD 2	8 (22.9)[a]	9 (27.3)	7 (19.4)	24 (23.1)[d]
SIMD 3	6 (17.1)[a]	3 (9.1)	3 (8.3)	12 (11.5)[d]
SIMD 4	4 (11.4)[a]	4 (12.1)	6 (16.7)	14 (13.5)[d]
SIMD 5 (least disadvantaged)	6 (17.5)[a]	5 (15.2)	5 (13.9)	16 (15.4)[d]

For peer review only - http://bmjopen.bmj.com/site/about/guidelines.xhtml

**Marital status, n (%)**				
Cohabiting	4 (11.1)	2 (6.3) [e]	5 (13.8)	11 (10.6)[d]
Divorced	2 (5.6)	0 (0.0) [e]	2 (5.6)	4 (3.9)[d]
Married	22 (61.1)	21 (65.6)[e]	17 (47.2)	60 (57.7)[d]
Separated	1 (2.8)	3 (9.4)[e]	2 (5.6)	6 (5.8)[d]
Single	7 (19.4)	5 (15.6)[e]	8 (22.2)	20 (19.2)[d]
Widowed	0 (0)	1 (3.1)[e]	1 (2.8)	2 (19.2)[d]
Prefer not to say	0 (0.0)	0 (0.0)[e]	1 (2.8)	1 (1.0)[d]
**Comorbidities, n (%)**				
Arthritis	11 (30.6)	3 (9.1)	8 (22.2)	22 (21.0)
Cancer	0 (0.0)	0 (0.0)	1 (2.8)	1 (1.0)
Diabetes	4 (11.1)	5 (15.2)	6 (16.7)	15 (14.3)
Myocardial infarction	2 (5.6)	1 (3.0)	0 (0.0)	3 (2.9)
Hypertension	7 (19.4)	8 (24.2)	7 (19.4)	22 (21.0)
Stroke (including TIA)	1 (2.8)	1 (3.0)	0 (0.0)	2 (1.9)
**Ethnic group, n (%)**				
Asian	2 (5.6)	1 (3.1)[e]	1 (2.8)	4 (3.8)[d]
Black	2 (5.6)	1 (3.1)[e]	1 (2.8)	4 (3.8)[d]
White	32 (88.8)	29 (90.7)[e]	34 (94.4)	95 (91.4)[d]
Prefer not to say	0 (0.0)	1 (3.1) [e]	0 (0.0)	1 (1.0)[d]
**Education**				

For peer review only - http://bmjopen.bmj.com/site/about/guidelines.xhtml

Bachelor Degree (=SVQ5)	7 (19.4)	6 (19.5)[b]	11 (30.6)	24 (23.3)[f]
HNC/ HND (=SVQ4)	4 (11.1)	6 (19.5)[b]	2 (5.6)	12 (11.6)[f]
Higher Grade/ Advanced Higher/ A-Level or equivalent (=SVQ3)	3 (8.3)	5 (16.1)[b]	1 (2.8)	9 (8.7)[f]
Masters/ PhD or equivalent	1 (2.8)	1 (3.2)[b]	3 (8.3)	5 (4.8)[f]
No formal qualifications	7 (19.4)	3 (9.7)[b]	10 (27.8)	20 (19.4)[f]
Standard Grade/GCSE/ Intermediate 1 or 2	6 (16.7)	7 (22.6)[b]	4 (11.1)	17 (16.5)[f]
Still studying	2 (5.6)	1 (3.2)[b]	3 (8.3)	6 (5.8)[f]
Vocational qualifications (=SVQ1+2)	4 (11.1)	1 (3.2)[b]	0 (0.0)	5 (4.8)[f]
Other	0 (0.0)	1 (3.2)[b]	0 (0.0)	1 (1.0)[f]
Prefer not to say	2 (5.6)	0 (0.0)[b]	2 (5.6)	4 (3.9)[f]
**Working status, n (%)**				
Full time student	4 (11.1)	2 (6.3)[e]	1 (2.8)	7 (6.7)[d]
Employed - Full time (30+ hours per week)	16 (44.4)	16 (50.0)[e]	18 (50.0)	50 (48.1)[d]
Employed - Part time (8-29 hours per week)	3 (8.3)	3 (9.4)[e]	0 (0.0)	6 (5.8)[d]
Self-employed	3 (8.3)	3 (9.4)[e]	1 (2.8)	7 (6.7)[d]
Not in paid work	2 (5.6)	2 (6.2)[e]	12 (33.3)	16 (15.4)[d]
Retired	8 (22.2)	6 (18.7)[e]	4 (11.1)	18 (17.3)[d]
Household size, mean (SD)	2.6 (1.3)	2.6 (1.0)	2.4 (1.4)	2.5 (1.3)

For peer review only - http://bmjopen.bmj.com/site/about/guidelines.xhtml

Note: [a] N=35, [b] N=31, [c] N=102, [d] N=104, [e] N=32, [f] N=103; BMI = Body Mass Index, CM = centimetres, GCSE = General Certificate of Secondary Education, kg = kilogram, N = overall participants, n = participants within specific category, SD = Standard Deviation, SIMD = Scottish index of multiple deprivation 1 represents the most disadvantaged area; quintile SIMD 5 represents the least disadvantaged area, SVQ = Scottish Vocational Qualification

For peer review only

Table 2 Programme satisfaction, and contamination at three, six and 12 months

	3 months		6 months		12 months		
	SMS + I (N=31)	SMS only (N=27)	SMS + I (N=23)	SMS only (N=21)	SMS + I (N=22)	SMS only (N=26)	Control (N=30)
Programme satisfaction (0-100), mean (SD)	80.3 (21.1)	75.0 (22.2)	76.2 (29.6)	79.0 (20.7)	80.9 (20.0)	77.0 (20.8)	87.3 (17.5)[a]
**Programme has been… (1 = low, 5 = high), mean (SD)**							
understandable	4.5 (0.9)	4.4 (0.8)[b]	4.4 (1.2)[c]	4.4 (1.2)	4.6 (1.1)[d]	4.6 (1.1)[e]	4.6 (0.9)[a]
useful	4.4 (0.9)	4.0 (1.0)	4.1 (1.3)	4.1 (1.1)[d]	4.1 (1.2)	4.0 (1.0)	4.3 (0.9)
helpful	4.3 (0.9)[f]	3.9 (1.0)	4.1 (1.3)	4.1 (1.1)[d]	4.1 (1.10)	4.0 (1.0)	4.3 (0.9)
interesting	4.3 (1.0)	4.2 (1.1)	4.2 (1.1)	4.4 (1.3)[d]	4.3 (0.8)	4.3 (1.2)	4.5 (1.0)
relevant	4.2 (1.1)	3.9 (1.2)	4.0 (1.3)	4.3 (1.1)[d]	4.1 (1.1)	4.2 (1.1)	4.5 (1.0)
**Helpfulness (1 = low, 5 = high), mean (SD)**							
Text messages	3.4 (1.5)	3.1 (1.3)	3.1 (1.3)	3.6 (1.3)	3.4 (1.3)	3.3 (1.4)	n/a

Website	3.3 (1.2)[g]	3.3 (0.9)[b]	3.3 (1.2)	3.2 (0.8)[d]	3.6 (1.0)	3.4 (0.7)[h]	3.5 (1.1) [b]
Pedometer	4.1 (1.2)[f]	4.1 (0.9)	3.7 (1.4)	4.0 (1.0)	3.9 (1.2)[i]	4.0 (1.0)[e]	3.7, (1.4)
**Met other men in programme, n (%)**							
Yes	0 (0.0)	0 (0.0)	1 (4.3)	0 (0.0)	1 (4.5)	0 (0.0)	1 (3.3)

Note. [a] N=29, [b] N=26, [c] N=22, [d] N=20, [e] N=25, [f] N=30, [g] N=28, [h] N=24, SD = Standard Deviation.

For peer review only

For peer review only - http://bmjopen.bmj.com/site/about/guidelines.xhtml

Table 3 Weight change at three, six, and 12 months

	3 months		6 months		12 months		
	SMS + I<br>N=36	SMS only<br>N=33	SMS + I<br>N=36	SMS only<br>N=33	SMS + I<br>N=36	SMS only<br>N=33	Control<br>N=36
**Weight change (kg), mean (SD)**							
Observed cases only	2.79 (3.50)[a]	-1.97 (3.97)[b]	-4.59 (5.62)[c]	-3.30 (4.92)[d]	-3.93 (5.74)[c]	-1.64 (5.64)[b]	-1.06 (6.29)[e]
BOCF	-2.40 (3.38)	-1.55 (3.60)	-2.93 (4.98)	-2.10 (4.21)	-2.51 (4.94)	-1.29 (5.03)	-0.86 (5.64)
LOCF	-2.40 (3.38)	-1.86 (3.88)	-3.38 (4.92)	-2.21 (4.39)	-2.98 (4.91)	-1.33 (5.04)	-0.86 (5.64)
**Weight change (%), mean (SD)**							
Observed cases only	-2.54 (3.47)[a]	-1.95 (3.72)[b]	-4.20 (5.54)[c]	-3.02 (4.22)[d]	-3.51 (5.83)[c]	-1.51 (4.65)[b]	-1.00 (5.31)[e]
BOCF	-2.18 (3.33)	-1.53 (3.39)	-2.69 (4.84)	-1.92 (3.65)	-2.24 (4.93)	-1.19 (4.16)	-0.80 (4.77)
LOCF	-2.18 (3.33)	-1.53 (3.39)	-3.11 (4.80)	-2.06 (3.85)	-2.68 (4.92)	-1.22 (4.16)	-0.80 (4.77)
**Weight change categories (observed cases only), n (%)**							

weight gain	6 (19.4)[a]	8 (30.8)[b]	2 (8.7)[c]	6 (28.6)[d]	5 (21.7)[c]	10 (38.5)[b]	14 (48.3)[e]
0-<3% weight loss	13 (41.9)[a]	10 (38.5)[b]	10 (43.5)[c]	7 (33.3)[d]	5 (21.7)[c]	9 (34.6)[b]	7 (24.1)[e]
≥3-<5% weight loss	4 (12.9)[a]	4 (15.4)[b]	3 (13.0)[c]	2 (9.5)[d]	3 (13.0)[c]	4 (15.4)[b]	0 (0.0)[e]
≥5-<10% weight loss	8 (25.8)[a]	2 (7.7)[b]	3 (13.0)[c]	4 (19.1)[d]	7 (30.4)[c]	2 (7.7)[b]	6 (20.7)[e]
≥10% weight loss	0 (0.0)[a]	2 (7.7)[b]	5 (21.7)[c]	2 (9.5)[d]	3 (13.0)[c]	1 (3.9)[b]	2 (6.9)
**BMI change, mean (SD)**							
Observed cases only	-0.89 (1.1) [a]	-0.73 (1.32)[b]	-1.45 (1.79)[c]	-1.04 (1.49)[d]	-1.24 (1.89)[c]	-0.49 (1.68)[b]	-0.47 (2.13)[e]
BOCF	-0.75 (1.07)	-0.59 (1.22)	-0.92 (1.58)	-0.66 (1.28)	-0.78 (1.60)	-0.37 (1.50)	-0.37 (1.96)
LOCF	-0.75 (1.07)	-0.59 (1.22)	-0.93 (1.56)	-0.69 (1.34)	-0.93 (1.59)	-0.38 (1.50)	-0.37 (1.96)
**Waist circumference change (cm), mean (SD)**							
Observed cases only	-3.74 (4.62) [a]	-3.14 (4.3)[b]	-4.70 (6.29)[c]	-3.14 (4.34)[d]	-4.40 (6.08)[c]	-2.30 (4.37)[b]	-2.26 (4.97)[e]
BOCF	-3.22 (4.47)	-2.49 (3.9)	-3.00 (5.49)	-2.00 (3.76)	-2.81 (5.82)	-1.82 (3.98)	-1.82 (4.53)
LOCF	-3.22 (4.47)	-2.49 (3.9)	-3.71 (5.76)	-2.23 (4.03)	-3.51 (4.48)	-1.85 (3.97)	-1.82 (4.53)

Note. [a] N=31, [b] N=26, [c] N=23, [d] N=21, BOCF = baseline observation carried forward, cm = centimetres, LOCF = last observation carried forward, kg = kilogram, M = mean, SD = Standard Deviation.

For peer review only

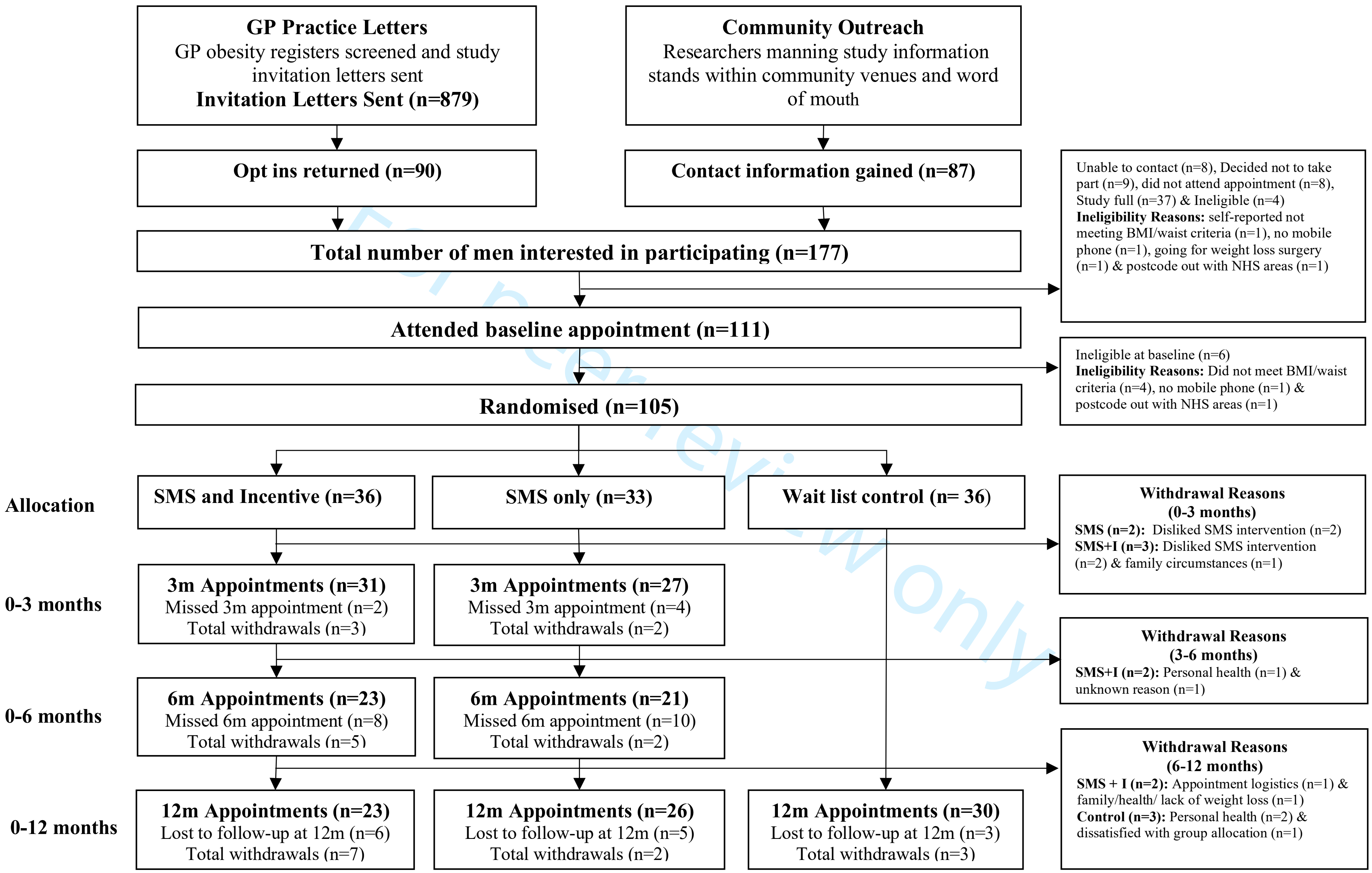

For peer review only - http://bmjopen.bmj.com/site/about/guidelines.xhtml

Online supplement 1: Incentive strategy contingent on meeting weight loss targets over 12 months

	Money secured	Money lost
3 months: £50 – target 5% weight loss		
<5%	£0	£50
≥5%	£50	£0
6 months: £150 – target 10%		
<5%	£0	£150
5%	£75	£75
6%	£90	£60
7%	£105	£45
8%	£120	£30
9%	£135	£15
≥10%	£150	£0
12 months: £200 – target 10%		
<5%	£0	£200
5%	£100	£100
6%	£120	£80
7%	£140	£60
8%	£160	£40
9%	£180	£20
≥10%	£200	£0

For peer review only

Online supplement 2 Intervention engagement over time for narrative texts and study webpage

	0-3 months		3-6 months		6-12 months	
	SMS + I N=36	SMS only N=33	SMS + I N=36	SMS only N=33	SMS + I N=36	SMS only N=33
Replies to texts, count	289	81	10	6	28	11
Replies to texts, mean (SD)	8.0 (24.2)	2.5 (7.9)	0.3 (0.7)	0.3 (1.0)	0.8 (3.3)	0.3 (1.0)
**Text replies, n (%)**						
0 replies	21 (58.3)	23 (69.7)	30 (83.3)	31 (93.9)	28 (77.8)	28 (84.8)
1-5 replies	9 (25.0)	7 (22.3)	6 (16.7)	2 (6.1)	7 (19.4)	5 (15.2)
6-10 replies	2 (5.6)	1 (3.0)	0 (0.0)	0 (0.0)	0 (0.0)	0 (0.0)
11-15 replies	0 (0.0)	0 (0.0)	0 (0.0)	0 (0.0)	0 (0.0)	0 (0.0)
16-20 replies	1 (2.8)	1 (3.0)	0 (0.0)	0 (0.0)	0 (0.0)	0 (0.0)
>20 replies	3 (8.3)	1 (3.0)	0 (0.0)	0 (0.0)	1 (2.8)	0 (0.0)

For peer review only - http://bmjopen.bmj.com/site/about/guidelines.xhtml

Stopping texts, n (%)	1 (2.8)	2 (6.1)	3 (8.3)	3 (9.1)	1 (2.8)	1 (3.0)
Withdrawal due to texts (self-report)	2 (5.6)	2 (6.1)	0 (0.0)	0 (0.0)	0 (0.0)	0 (0.0)
**Participants using website self-monitoring features, n (%)**						
Weight	8 (22.2)	5 (15.2)	6 (16.7)	3 (9.1)	4 (11.1)	3 (9.1)
Steps	10 (27.8)	6 (18.2)	7 (19.4)	2 (6.1)	3 (8.3)	2 (6.1)
Waistline	4 (11.1)	3 (9.1)	3 (8.3)	0 (0.0)	1 (2.8)	0 (0.0)
Belt notches	2 (5.6)	0 (0.0)	2 (5.6)	0 (0.0)	0 (0.0)	0 (0.0)
**Number self-monitoring features used, n (%)**						
None	24 (66.7)	26 (78.8)	28 (77.8)	30 (90.1)	31 (86.1)	29 (87.9)
One	5 (13.9)	2 (6.1)	2 (5.6)	1 (3.1)	3 (8.3)	3 (9.1)
Two	3 (8.3)	3 (9.1)	3 (8.3)	2 (6.1)	1 (2.8)	1 (3.0)

For peer review only

Three	3 (8.3)	2 (6.1)	2 (5.6)	0 (0.0)	1 (2.8)	0 (0.0)
Four	1 (2.8)	0 (0.0)	1 (2.8)	0 (0.0)	0 (0.0)	0 (0.0)
**Number of entries on self-monitoring feature, count**						
Weight	69	115	45	23	42	46
Steps	419	296	350	102	397	74
Waistline	4	7	5	0	2	0
Belt notches	8	0	4	0	0	0

Note. M = mean, SD = Standard Deviation.

For peer review only

For peer review only - http://bmjopen.bmj.com/site/about/guidelines.xhtml

Online supplement 3 Satisfaction and helpfulness ratings for overall programme and narrative texts at 12 months by deprivation status

	SMS+I		SMS only		Total	
	SIMD 1, 2 (n=14)	SIMD 3+ (n=7)	SIMD 1, 2 (n=16)	SIMD 3+ (n=10)	SIMD 1, 2 (n=30)	SIMD 3+ (n=17)
Satisfaction programme[a]	81.8, 21.7	79.9, 19.2	74.6, 23.5	81.0, 16.0	77.9, 22.6	80.5, 16.8
Helpfulness programme[b]	4.2, 1.1	3.9, 1.1	3.9, 1.1	4.2, 1.0	4.1, 1.1	4.1, 1.0
Helpfulness of narrative texts[b]	3.4, 1.3	3.4, 1.4	3.0, 1.5	3.8, 1.2	3.2, 1.4	3.6, 1.3

Note. All data mean (SD), [a] scored 0-100 (higher = more satisfied), [b] scored 1-5 **(**1 = low, 5 = high**),** SIMD = Scottish index of multiple deprivation 1 represents the most disadvantaged area; quintile SIMD 5 represents the least disadvantaged area SMS = Short message service, SMS+I = SMS plus incentive group.

For peer review only

CONSORT 2010 checklist of information to include when reporting a pilot or feasibility trial*

Section/Topic	Item No	Checklist item	Reported on page No
**Title and abstract**			
	1a	Identification as a pilot or feasibility randomised trial in the title	1
	1b	Structured summary of pilot trial design, methods, results, and conclusions (for specific guidance see CONSORT abstract extension for pilot trials)	3, 4
**Introduction**			
Background and objectives	2a	Scientific background and explanation of rationale for future definitive trial, and reasons for randomised pilot trial	6, 7
	2b	Specific objectives or research questions for pilot trial	7
**Methods**			
Trial design	3a	Description of pilot trial design (such as parallel, factorial) including allocation ratio	7
	3b	Important changes to methods after pilot trial commencement (such as eligibility criteria), with reasons	n/a
Participants	4a	Eligibility criteria for participants	8
	4b	Settings and locations where the data were collected	8,9
	4c	How participants were identified and consented	8,9
Interventions	5	The interventions for each group with sufficient details to allow replication, including how and when they were actually administered	10, 11
Outcomes	6a	Completely defined prespecified assessments or measurements to address each pilot trial objective specified in 2b, including how and when they were assessed	11-13
	6b	Any changes to pilot trial assessments or measurements after the pilot trial commenced, with reasons	n/a
	6c	If applicable, prespecified criteria used to judge whether, or how, to proceed with future definitive trial	7
Sample size	7a	Rationale for numbers in the pilot trial	8
	7b	When applicable, explanation of any interim analyses and stopping guidelines	n/a
Randomisation:			
Sequence generation	8a	Method used to generate the random allocation sequence	9
	8b	Type of randomisation(s); details of any restriction (such as blocking and block size)	9
Allocation concealment mechanism	9	Mechanism used to implement the random allocation sequence (such as sequentially numbered containers), describing any steps taken to conceal the sequence until interventions were assigned	9

For peer review only

Implementation	10	Who generated the random allocation sequence, who enrolled participants, and who assigned participants to interventions	9
Blinding	11a	If done, who was blinded after assignment to interventions (for example, participants, care providers, those assessing outcomes) and how	9
	11b	If relevant, description of the similarity of interventions	n/a
Statistical methods	12	Methods used to address each pilot trial objective whether qualitative or quantitative	11-13
**Results**			
Participant flow (a diagram is strongly recommended)	13a	For each group, the numbers of participants who were approached and/or assessed for eligibility, randomly assigned, received intended treatment, and were assessed for each objective	40
	13b	For each group, losses and exclusions after randomisation, together with reasons	40
Recruitment	14a	Dates defining the periods of recruitment and follow-up	15
	14b	Why the pilot trial ended or was stopped	15
Baseline data	15	A table showing baseline demographic and clinical characteristics for each group	31-33
Numbers analysed	16	For each objective, number of participants (denominator) included in each analysis. If relevant, these numbers should be by randomised group	31-45
Outcomes and estimation	17	For each objective, results including expressions of uncertainty (such as 95% confidence interval) for any estimates. If relevant, these results should be by randomised group	15-19
Ancillary analyses	18	Results of any other analyses performed that could be used to inform the future definitive trial	n/a
Harms	19	All important harms or unintended effects in each group (for specific guidance see CONSORT for harms)	19
	19a	If relevant, other important unintended consequences	n/a
**Discussion**			
Limitations	20	Pilot trial limitations, addressing sources of potential bias and remaining uncertainty about feasibility	20
Generalisability	21	Generalisability (applicability) of pilot trial methods and findings to future definitive trial and other studies	22
Interpretation	22	Interpretation consistent with pilot trial objectives and findings, balancing potential benefits and harms, and considering other relevant evidence	22
	22a	Implications for progression from pilot to future definitive trial, including any proposed amendments	22
**Other information**			
Registration	23	Registration number for pilot trial and name of trial registry	4
Protocol	24	Where the pilot trial protocol can be accessed, if available	7
Funding	25	Sources of funding and other support (such as supply of drugs), role of funders	24,25
	26	Ethical approval or approval by research review committee, confirmed with reference number	7

For peer review only

Citation: Eldridge SM, Chan CL, Campbell MJ, Bond CM, Hopewell S, Thabane L, et al. CONSORT 2010 statement: extension to randomised pilot and feasibility trials. BMJ. 2016;355.
*We strongly recommend reading this statement in conjunction with the CONSORT 2010, extension to randomised pilot and feasibility trials, Explanation and Elaboration for important clarifications on all the items. If relevant, we also recommend reading CONSORT extensions for cluster randomised trials, non-inferiority and equivalence trials, non-pharmacological treatments, herbal interventions, and pragmatic trials. Additional extensions are forthcoming: for those and for up to date references relevant to this checklist, see www.consort-statement.org.

For peer review only

BMJ Open

Game of Stones: Feasibility randomised controlled trial of how to engage men with obesity in text message and incentive interventions for weight loss

Journal:	*BMJ Open*
Manuscript ID	bmjopen-2019-032653.R2
Article Type:	Original research
Date Submitted by the Author:	06-Dec-2019
Complete List of Authors:	Dombrowski, Stephan U; University of New Brunswick Fredericton, Kinesiology<br>McDonald, Matthew; University of Stirling<br>van der Pol, Marjon; University of Aberdeen, Health Economics Research Unit<br>Grindle, Mark; University of the Highlands and Islands<br>Avenell, Alison; University of Aberdeen<br>Carroll, Paula; Mens Health Forum in Ireland<br>Calveley, Eileen; University of Stirling<br>Elders, A; Glasgow Caledonian University School of Health and Life Sciences, NMAHP Research Unit ; Glasgow Caledonian University<br>Glennie, Nicola ; University of Stirling<br>Gray, Cindy; University of Glasgow, Institute of Health and Wellbeing<br>Harris, Fiona; University of Stirling,<br>Hapca, Adrian; University of Dundee, Population Health Sciences<br>Jones, Claire; University of Dundee<br>Kee, Frank; Queen's University Belfast, UKCRC Centre of Excellence for Public Health (NI)<br>McKinley, Michelle C.; Queen's University Belfast<br>Skinner, Rebecca; University of Stirling<br>Tod, Martin; Mens Health Forum<br>Hoddinott, Pat; University of Stirling, Nursing, Midwifery and Allied Health Professional Research Unit
**Primary Subject Heading**:	Public health
Secondary Subject Heading:	Public health
Keywords:	obesity, men, feasibility, trial, SMS, weight loss

SCHOLARONE™ Manuscripts

I, the Submitting Author has the right to grant and does grant on behalf of all authors of the Work (as defined in the below author licence), an exclusive licence and/or a non-exclusive licence for contributions from authors who are: i) UK Crown employees; ii) where BMJ has agreed a CC-BY licence shall apply, and/or iii) in accordance with the terms applicable for US Federal Government officers or employees acting as part of their official duties; on a worldwide, perpetual, irrevocable, royalty-free basis to BMJ Publishing Group Ltd ("BMJ") its licensees and where the relevant Journal is co-owned by BMJ to the co-owners of the Journal, to publish the Work in this journal and any other BMJ products and to exploit all rights, as set out in our licence.

The Submitting Author accepts and understands that any supply made under these terms is made by BMJ to the Submitting Author unless you are acting as an employee on behalf of your employer or a postgraduate student of an affiliated institution which is paying any applicable article publishing charge ("APC") for Open Access articles. Where the Submitting Author wishes to make the Work available on an Open Access basis (and intends to pay the relevant APC), the terms of reuse of such Open Access shall be governed by a Creative Commons licence – details of these licences and which Creative Commons licence will apply to this Work are set out in our licence referred to above.

Other than as permitted in any relevant BMJ Author's Self Archiving Policies, I confirm this Work has not been accepted for publication elsewhere, is not being considered for publication elsewhere and does not duplicate material already published. I confirm all authors consent to publication of this Work and authorise the granting of this licence.

For peer review only

Game of Stones: Feasibility randomised controlled trial of how to engage men with obesity in text message and incentive interventions for weight loss

Stephan U Dombrowski[1, 2], Matthew McDonald[3], Marjon van der Pol[4], Mark Grindle[5], Alison Avenell[6], Paula Carroll[7], Eileen Calveley[3], Andrew Elders[8], Nicola Glennie[3], Cindy M Gray[9], Fiona M Harris[3], Adrian Hapca[10], Claire Jones[11], Frank Kee[12], Michelle C McKinley[13], Rebecca Skinner[2], Martin Tod[13], Pat Hoddinott[3]

Corresponding author:

Stephan U Dombrowski

Assistant Professor

Faculty of Kinesiology

90 Mackay Drive

Fredericton, New Brunswick,

Canada, E3B 5A3,

Email: stephan.dombrowski@unb.ca

Tel: 1 506 453 4803

[1]Faculty of Kinesiology, University of New Brunswick

[2]Division of Psychology, University of Stirling

[3]Nursing, Midwifery and Allied Health Professions Research Unit, University of Stirling

[4]Health Economics Research Unit, University of Aberdeen

[5]Institute for Health Research and Innovation, University of the Highlands and Islands

[6]Health Services Research Unit, University of Aberdeen

[7]Men's Health Forum in Ireland

[8]Nursing, Midwifery and Allied Health Professions Research Unit, Glasgow Caledonian University

For peer review only

For peer review only - http://bmjopen.bmj.com/site/about/guidelines.xhtml

[9]Institute of Health & Wellbeing, University of Glasgow

[10]Tayside Clinical Trials Unit, University of Dundee

[11]Health Informatics Centre, University of Dundee

[12] Centre for Public Health, Queen's University Belfast

[13]The Men's Health Forum (GB), London

Word count: 5624

Key words: obesity, men, feasibility, trial, SMS, weight loss, behaviour change, financial incentives

For peer review only

ABSTRACT

Objectives

To examine the acceptability and feasibility of narrative text messages with or without financial incentives to support weight loss for men.

Design

Individually randomised three-arm feasibility trial with 12 months' follow-up.

Setting

Two sites in Scotland with high levels of disadvantage according to Scottish Index for Multiple Deprivation (SIMD).

Participants

Men with obesity (n=105) recruited through community outreach and general practitioner registers.

Interventions

Participants randomised to a) narrative text messages plus financial incentive for 12 months (SMS+I), b) narrative text messages for 12 months (SMS only), or c) waiting list control.

Outcomes

Acceptability and feasibility of recruitment, retention, intervention components and trial procedures assessed by analysing quantitative and qualitative data at three, six and 12 months.

Results

105 men were recruited, 60% from more disadvantaged areas (SIMD quintiles 1 or 2). Retention at 12 months was 74%. Fewer SMS+I participants group (64%) completed 12-month assessments compared to SMS only (79%) and control (83%). Narrative texts were acceptable to many men, but some reported negative reactions. No evidence emerged that level of disadvantage was related to acceptability of narrative texts. Eleven SMS+I participants (31%) successfully met or partially met weight loss targets. The cost of the incentive per participant was £81.94 (95% CI £34.59-£129.30). Incentives were acceptable, but improving health was reported as the key motivator for weight loss. All groups lost

For peer review only

weight (SMS+I -2.51kg (SD=4.94); SMS only -1.29kg (SD=5.03); control -0.86kg (SD=5.64) at 12 months).

Conclusions

This three-arm weight management feasibility trial recruited and retained men from across the socioeconomic spectrum with the majority from areas of disadvantage, was broadly acceptable to most participants and feasible to deliver.

Trial registration

ClinicalTrials.gov Identifier: NCT03040518

Funding details

Funded by the National Institute for Health Research Public Health Research Programme (14/185).

For peer review only

For peer review only - http://bmjopen.bmj.com/site/about/guidelines.xhtml

STRENGTHS AND LIMITATIONS OF THIS STUDY

- This weight management study for men with obesity recruited men across the socio-economic spectrum, with a specific focus on more disadvantaged areas, to a randomised controlled feasibility study examining SMS with or without endowment incentives.
- Acceptability and feasibility were established using a multi-lens perspective drawing on quantitative, qualitative and trial procedure data.
- Effectiveness of intervention components will need to be established in a full multi-centre trial.

For peer review only

For peer review only - http://bmjopen.bmj.com/site/about/guidelines.xhtml

BACKGROUND

Obesity is associated with an increased risk of serious health conditions such as type 2 diabetes, cardiovascular disease and some cancers.[1] In 2016, 26% of men in the UK were classified as obese, and men were less likely to be a healthy weight than women (31% of men compared with 35% of women).[2 3]

Despite the growing prevalence of obesity, men contribute a disproportionately low number of participants to evidence-based weight management programmes.[4] Most interventions are designed for mixed sex populations, but systematic review evidence suggests that, compared to women, men often benefit from different ways of providing interventions.[4]

Text message interventions

The evidence for text message interventions supporting changes in lifestyle behaviours, including behaviour change for weight loss, is promising.[5-7] However, systematic reviews[4-7] report no text message delivered trials that target weight loss designed for men only. Text-based interventions can reach large numbers of people, including men from disadvantaged backgrounds,[8] and mobile technologies such as standard mobile phones allow delivery of evidence-based strategies anywhere and anytime.

Narrative approaches to promoting behaviour change have been suggested as a tool for communication-based interventions.[9] Narrative SMS can be broadly defined as interactive life stories, which are based around a group of characters with whom recipients can identify.[10] Text message based interventions using narratives have been used to engage hard to reach men in moderating their alcohol consumption, and were found to be acceptable.[11-13] Narrative approaches to weight management in men may be a promising tool to support behaviour and weight change.

Financial incentive interventions

Systematic review evidence of financial incentives for behaviour change highlights the potential for incentives to change behaviours in low-income adults[14] and help reduce health inequalities.[15] However, little evidence exists that focuses on using financial incentives to support weight management in individuals from disadvantaged backgrounds[16 17], particularly for men[4]. A discrete choice experiment found that paying people (£10-£30/week varying by age and weight) to take part in diet and physical activity (PA) interventions is likely to improve uptake, adherence and maintenance of behaviour change.[18] Moreover, the evidence

For peer review only - http://bmjopen.bmj.com/site/about/guidelines.xhtml

for financial incentives for weight loss is growing.[19-22] In particular, deposit contracts, where participants deposit their own money and are reimbursed only if they achieve the target weight loss, are effective whilst the incentives are in place.[4 23] Deposit contracts draw on loss aversion where people are more motivated to avoid losses than they are to achieve similarly sized gains.[24 25] However, deposit contracts may not be not equitable, as committing one's own money up front may not be possible, particularly for individuals with lower income.

Endowment incentive contracts may overcome limitations of deposit contracts. Endowment incentive contracts are financial incentives where a person is endowed with a (hypothetical) amount of money which they either 'secure' or 'lose' depending on achieving certain targets. A trial that framed financial incentives in this way found these incentives were effective for achieving PA goals in the short term.[26]

Aim

The aim of the Game of Stones feasibility trial was to examine the acceptability and feasibility of a men-only weight management intervention consisting of narrative text messages, with and without an endowment incentive, compared to waiting list control. The objectives were to:

1. Assess the acceptability and willingness to be randomised to: i) narrative text message and endowment incentives; ii) narrative text messages only; or iii) waiting list for text messages (control).
2. Assess the feasibility of recruiting from general practitioner (GP) practice obesity registers and community venues.
3. Determine the acceptability of intervention content, feasibility of delivery, fidelity and any unintended consequences.
4. Assess indicative effects on weight change and progression criteria for a full trial.

METHODS

CONSORT guidance for reporting randomised pilot and feasibility studies was followed[27 28].

Trial design

A three-arm individually randomised parallel-group controlled feasibility trial was conducted with an integrated qualitative and quantitative mixed methods approach[29]. Informed by MRC guidance on the evaluation of complex interventions, the study included an integrated mixed methods process evaluation[30 31]. Drawing on both qualitative and quantitative approaches

For peer review only - http://bmjopen.bmj.com/site/about/guidelines.xhtml

For peer review only

allowed the team to explore participants views (acceptability) of the intervention with participants as well as explore implementation processes such as recruitment, retention and barriers and facilitators to these.

Participants were randomised to receive narrative text messages and endowment incentives (SMS+I), narrative text messages only (SMS only), or a waiting list for text messages (Control). The protocol to the full study is available online: https://www.journalslibrary.nihr.ac.uk/programmes/phr/1418509/#/

Ethical approval for the study was obtained from the North of Scotland Research Ethics Service (Ref: 16/NS/0120).

Setting

Two Health Board areas in Scotland (Sites A and B) with high levels of disadvantage were the setting for this study (see participant recruitment section for additional details).

Eligibility criteria

Participants met the following eligibility criteria: men over 18 years old; BMI ≥30kg/m^2 and/or a waist circumference of ≥40 inches (102 cm); owned a mobile phone capable of receiving text messages; could understand English language text messages; not already taking part in a weight loss study; not planning or waiting to have bariatric surgery; not planning to move within the next 12 months; considered by practice clinical staff as suitable for participation (GP practice recruitment only), for example, no severe medical, terminal or psychiatric illness (in patient or close family member) or no significantly impaired cognitive function.

Sample size

This study aimed to randomise 105 men, 35 to each arm, in line with recent recommendations for pilot trials as sufficient to estimate key parameters for a full trial.[32] A sample size of 35 per arm is sufficient to allow the population variance to be estimated (e.g. the SD in weight loss) with enough precision to deliver at least 80% power and 90% confidence in a full trial, with a standardised effect size between 0.2 and 0.5.[32]

Participant recruitment

Participants were recruited via: 1) community outreach, and 2) GP practice obesity registers.

For peer review only - http://bmjopen.bmj.com/site/about/guidelines.xhtml

Community outreach

Community recruitment strategies that report success for recruiting men in disadvantaged areas were used.[11-13] This included researchers working on stands with study information leaflets and table banners in supermarkets, fitness centres, hospital foyers, health centres, council workplaces and community centres across both sites. Eleven of the 13 recruitment venues were within more disadvantaged areas based on the Scottish Index of Multiple Deprivation (SIMD quintiles 1 and 2).[33] Men who showed interest were given study information and asked to leave their contact details with researchers.

In addition, some men were recruited through word of mouth. This included researcher discussions with family, friends, colleagues and local workers who passed on study information to eligible men they knew. Information leaflets were distributed across localities in shops, libraries, barbers and community centres. Interested men then contacted researchers to get more information.

GP practice obesity register letters

Practices within more disadvantaged areas (SIMD quintiles 1 or 2) were invited to participate in the study. Of the 33 practices invited (n= 13 in Site A, n=20 in Site B), five practices participated (n=4 in Site A, n=1 in Site B).

Practice database searches identified potentially eligible men with a documented BMI of at least 30kg/m^2. Lists were screened by clinical practice staff and details passed to the Health Informatics Centre at the University of Dundee who sent out GP-headed study invitation letters and information leaflets. Interested men contacted the research team or returned an 'opt-in' card.

Baseline Appointment

Men interested in participating were invited to attend a face-to-face appointment. Detailed study information was discussed and written informed consent provided. Anthropometric measurements were conducted and eligibility assessed. Participants then completed a baseline questionnaire, and were randomised. Group-specific post-randomisation information was provided and discussed with the participant.

For peer review only - http://bmjopen.bmj.com/site/about/guidelines.xhtml

Randomisation

At the baseline appointment, independent randomisation was performed by the researcher using the clinical trials unit's secure remote web-based system, stratifying by recruitment method (GP and community) and recruitment site (Site A and Site B).

Blinding

The two recruitment researchers who also assessed outcomes and conducted qualitative interviews were mostly not blinded to group allocations. All other study team members were blinded. A demonstration of the feasibility for researcher outcome assessors to be blind to group allocation was conducted with eleven participants at the six month assessment. In this case, a researcher who had not previously met the participant arranged and undertook the assessment. These participants all complied with the request not to reveal their group allocation.

The trial statistician was fully blinded to intervention groups, and partially blinded for the control group due to the different response schedule (control participants were only assessed at baseline months and 12 months to reflect "usual life").

Intervention components

Narrative text messages

A narrative text message library consisting of 604 texts was written by a professional scriptwriter/researcher (MG) who designed the overall narrative with enough interlinked stories to engage participants over 12 months. Full details of the narrative texts development process are available elsewhere.[10] Narrative texts were sent to participants over the course of 12 months and were written from the point of view of a fictional character aiming to lose weight over 12 months. Narrative texts were written to appeal to men from disadvantaged backgrounds. All participants in the SMS+I and SMS only groups received automated texts according to a pre-determined schedule. Participants could reply to texts but did not receive a response. Texts were sent between 8:00AM and 10:00PM and ranged from 0-5 texts per day depending in the requirements of the narrative approach used. All participants were scheduled to receive the same number of text messages. Some texts were personalised including participants' names and men could select whether weight information should be presented in kilogrammes or stones and pounds. No further personalisation and tailoring

For peer review only - http://bmjopen.bmj.com/site/about/guidelines.xhtml

options were offered. Texts were delivered by the Health Information Centre in Dundee using existing automated technology linked to a clinical trials unit database.

Endowment incentive

The incentive strategy was informed by existing evidence and men's preferences elicited using a Discrete Choice Experiment completed by 1,045 men with obesity and reported elsewhere.[10] SMS+I participants were 'endowed' with a £400 incentive at baseline which was placed into a hypothetical personal account at the University of Stirling and given a mock-up personalised cheque. The full £400 could be secured by meeting weight loss targets at researcher assessments: 5% of body weight lost since baseline at three months (£50 secured/lost), 10% lost since baseline at six months (£150 secured/lost) and 10% lost since baseline at 12 months (£200 secured/lost) (see online supplement 1). Weight loss was verified at all face-to-face appointments. At six and 12 months, men lost a proportion of the money for each % weight loss not attained between 5-10%. Weight at 12 months had to be less than at baseline to receive any money, regardless of whether interim weight loss targets had been met. Men received the money by direct bank transfer after the 12-month assessment. Feedback on meeting incentive targets was sent by automated text message and displayed on the personalised SMS+I webpage.

Website

All trial participants were provided with a unique login ID for the Game of Stones website. The front page was accessible to all participants and included trial information and links to existing evidence-based online weight management resources. SMS +I and SMS only webpages had a brief biography and images of the fictional characters featured in narrative texts. Participants could enter their weight, pedometer steps, waist circumference and belt notches, which were displayed as basic visual progress charts. Only individual performance was displayed on the webpage, and no group averages were shown for social comparison. SMS+I webpages described the financial incentives and a visual progress chart of money secured/lost.

Printed information

All participants received a weight loss fact sheet (British Dietetic Association, Weight Loss Food Fact Sheet) and a small card which could be carried in their wallet for noting website details and their appointment weight, weight loss targets and appointments.

For peer review only - http://bmjopen.bmj.com/site/about/guidelines.xhtml

Pedometer

All participants received a study pedometer (3DFitBud, A420S, manufactured by 3DActive).

Comparator group

At baseline, participants received access to the information section of the webpage, printed information (i.e. a weight loss fact sheet) and a pedometer. They attended a baseline and 12-month appointment only. Control group participants were offered texts for three months commencing after the 12 month data collection point.

Outcomes

The outcomes for this study related to whether the design of Game of Stones was both acceptable and feasible to deliver as a full scale randomised controlled trial. An independent study steering committee advised whether the following pre-specified progression criteria in the study protocol were met sufficiently to proceed to a full trial.

1. Acceptability of the intervention and the control group (by the majority of the target group); willingness to be randomised.
2. Feasibility of recruiting 105 men in four months.
3. 12-month outcomes on at least 72% of men randomised per group, consistent with a recent UK weight management trial in men[34] and systematic reviews of male obesity literature[4].
4. Evidence of mean weight loss of at least 3% of baseline weight at 12 months in any intervention group.
5. Commitment by, for example, government or NHS/local authorities to fund the incentive intervention to ensure translation and sustainability.

Outcome assessment

Outcomes were assessed at baseline, three, six and 12 months for intervention participants (SMS+I and SMS only), and at baseline and 12 months for control participants. Individual appointments were at community centres, universities, NHS clinical research facilities, voluntary sector organisations, GP practice premises, or the participant's home if no suitable alternative venue could be found. At the 12 month appointment, all participants received a £20 voucher as reimbursement for their time. No travel expenses were provided.

The text message delivery system automatically the frequency of responses received to the texts. The website automatically recorded engagement with self-monitoring tools.

For peer review only - http://bmjopen.bmj.com/site/about/guidelines.xhtml

Self-report questionnaires were completed during appointments and measured socio-demographics, co-morbidities, disability, ethnicity, and perceptions on intervention acceptability. Overall satisfaction with the intervention was assessed with the item: "*On a scale from 0 (not satisfied at all) to 100 (completely satisfied). How satisfied are you with the Game of Stones programme?*". Acceptability of the intervention was assessed with the stem "*Overall the Game of Stones programme has been...*" followed by options '*understandable*', '*useful*', '*helpful*', '*interesting*' and '*relevant*', with responses captured on a 5-point scale ranging from '*totally disagree*' to '*totally agree*'. Helpfulness of the intervention was assessed with the item "*How helpful have you found the following in helping you lose weight?*" followed by options '*text messages*', '*website*', and '*pedometer*', with responses captured on a 5-point scale ranging from '*totally unhelpful*' to '*totally helpful*'. Measures of overall satisfaction, acceptability and helpfulness were adapted from Dombrowski et al., (2012).[35]

Anthropometry measures included height measured at baseline using a portable standing stadiometer (Seca 217, Birmingham, UK) to the nearest 0.1cm. Prior to weight measurements, participants removed shoes and bulky clothing and items from their pockets. Weight was recorded using portable calibrated electronic scales (Marsden M420, Rotherham, UK) to the nearest 0.01kg and waist circumference using a tape measure (Seca 203, Birmingham, UK) to the nearest 0.1cm.

Information on possible adverse events was recorded at assessment visits via open questions and through automatic monitoring of text message replies including words like 'suicide', 'die', and 'death' indicative of potential adverse events, which were notified to the research team by email.

Any negative participant reactions to their randomised group were recorded in researcher field notes. Willingness to be randomised was assessed by recording the number of participants refusing randomisation.

Interviews took place during three and 12 month appointments and were conducted face-to-face and detailed methods are described elsewhere.[10] All audio recorded interviews were transcribed verbatim and anonymised. At three months, all SMS+I and SMS only men were invited to participate in either a brief feedback or an in-depth interview (participant choice). The three month topic guide focussed on the early acceptability of the intervention components in order to identify any refinements required over the remaining nine month

For peer review only - http://bmjopen.bmj.com/site/about/guidelines.xhtml

intervention. For the 12 month qualitative interviews, separate topic guides for the three trial groups were informed by the analysis of the three-month interviews, researcher field notes and to gain information power[36] to address the study objectives. Purposive sampling from the three trial groups was informed by the three month interview data and researcher field notes to provide diversity of perspectives. Researcher field notes taken at all assessments were referred to when interpreting interview data.

Qualitative interviews were conducted face-to-face with 50 of 58 men who attended the three month assessment (7-61 minutes, median=23 minutes). At 12 months, interviews were conducted with 14 participants from SMS+I (13-65 minutes, median=33 minutes), 13 from SMS only (10-59 minutes, median=28 minutes) and six from the control group (7-18 minutes, median=11 minutes). Fourteen interviews at 12 months lasted <20 minutes, mostly for control participants who had received no contact from the research team between baseline and 12 months.

Analysis

Quantitative analysis

All continuous variables were summarised and tabulated using the following descriptive statistics: N (number of valid non-missing responses), mean, standard deviation (SD). Likert-scale variables were treated as continuous measures. The frequency and percentages (based on the non-missing sample size) of observed levels are reported for all categorical measures. The proportion of individuals contacted who were recruited and the proportions retained and withdrawn at each assessment by group was determined. Missing weight data are presented as baseline observation carried forward (BOCF) and last observation carried forward (LOCF) in addition to observed cases only.

Qualitative and mixed method data analysis

Anonymised transcripts of interviews together with: attributes for recruitment channel; attendance at assessments, trial group and participant characteristics and weight loss outcomes were entered into NVivo12 software for analysis guided by the framework approach.[37] Reference to researcher field notes contributed to interview data interpretation. Iterative data collection and analysis were driven by the key feasibility and acceptability research questions and objectives. A coding frame was developed by three researchers independently reading a diverse sample of six interviews, followed by a team discussion to finalise the coding frame and identify key themes. Independent coding was conducted by four

For peer review only - http://bmjopen.bmj.com/site/about/guidelines.xhtml

researchers (EC, NG, MM and RS) and checked for consistency by FH. Two independent researchers (EC and NG) who were not involved in any other aspect of the study, assisted with coding and analysis to enhance rigour and reliability of data analysis. Emergent themes and interpretive analysis were discussed at weekly researcher meetings and at two Co-Investigator qualitative interpretation meetings.

At the final stage of mixed methods data interpretation, the quantitative attributes on NVivo were drawn on to triangulate the analysis and suggest further avenues for interrogation. Matrix coding queries in NVivo were generated to cross-reference attributes for trial group, participant SIMD and weight loss outcome at 12 months, with nodes coded for views of incentives and texts messages respectively. Credibility and reliability of qualitative analysis was enhanced by independent coding, use of memos to ensure transparency of interpretation, and interpretive charting conducted with input from the wider study team.

Patient and Public Involvement

A continuous and responsive approach to patient and public involvement (PPI) was adopted to prepare the grant application and throughout the study, as described by Gamble and colleagues.[38]

Continuous PPI was provided by a Co-Investigator partnership with the Charities Men's Health Forum GB and Men's Health Forum in Ireland. The partnership commenced in 2011 with the ROMEO evidence syntheses of weight loss interventions for men with obesity.[4]

During the study, Co-Investigators from each of the Men's Health Forum charities attended trial management meetings to contribute to decisions, intervention development, data analysis, interpretation of findings and reporting. They provided feedback on the grant application, protocol, text messages, information materials and engaged wider involvement of men from their organisation to assist with appropriate language.[39]

Continuous PPI at the study oversight level was provided by two independent lay members of the study steering committee which met on three occasions.

Responsive PPI occurred at the study funding application stage. Co-Investigator CG engaged men who provided PPI input in the FFIT Trial.[34] During the study, relevant members of the public were identified through several sources including: Men's Health Forum GB, Men's Health Forum in Ireland, Scottish Community Health Councils, Men's Sheds, University of Stirling PPI group, Alliance Scotland and other Co-Investigator contacts. Effort was made to

For peer review only - http://bmjopen.bmj.com/site/about/guidelines.xhtml

engage men from more disadvantaged communities. A total of 121 PPI contributors were involved in a range of study activities, including finding a study name, helping with wording of information material, and helping with designing the intervention content. For full details see the report to the funder.[10]

RESULTS

Recruitment and retention

Recruitment was completed in four months between March to June 2017 meeting the target number of 105 men randomised (Figure 1).

Overall, 177 men expressed an interest in the study. Baseline appointments were attended by 111 (63%), of which six (5%) were ineligible. All 105 men were willing to be randomised and consented to be randomised to the SMS+I (n=36), SMS only (n=33) and control (n=36) groups.

GP practice recruitment was undertaken in five GP practices (Site A n=4, Site B n=1) with 45 men recruited and randomised through GP practices. The additional 60 men were recruited through community outreach.

--- Figure 1 ---

The 12 month assessment was completed by 79/105 participants (75%). One of the 79 participants (control group) could not attend and provided self-reported information because he was out of the region due to work commitments, which means that 74% (78/105) of participants were retained for weight assessments at 12 months. Overall 12 month retention differed by group (SMS+I=23/36, 64%; SMS only=26/33, 79%; control=30/36, 83%). Fourteen participants (13%) were lost to follow-up, and 12 participants (11%) withdrew. Reasons for withdrawal were dislike of narrative texts (n=4), health (n=3), family (n=1), unknown (n=1), dissatisfaction with group allocation (n=1), appointment logistics (n=1) and multiple reasons (n=1).

Baseline characteristics

Baseline characteristics are displayed in Table 1. On average, participants were 52.2 (SD=13.1) years old, had a BMI of 35.7 (SD=5.9) and a waist circumference of 116.8 cm (SD=11.8). BMI ranged from 27.5 kg/m^2 to 62.5kg/m^2. Twelve participants (11%) had a baseline BMI less than 30 kg/m^2 due to the additional weight circumference entry criterion of ≥40 inches (102 cm).

For peer review only - http://bmjopen.bmj.com/site/about/guidelines.xhtml

The majority of men lived in disadvantaged areas defined as SIMD quintiles 1 or 2 (n=62/104; 60%). Most participants were married (58%), reported at least one co-morbidity (63%), were of white ethnicity (91%), reported having children (76%), and were in full-time employment (48%).

--- Table 1 ---

Intervention fidelity

The intervention components were feasible to deliver with fidelity. The majority (95.4%) of the 38,214 text messages sent to the two intervention groups were delivered to the mobile phone, with 1,782 (4.6%) having no delivery status (i.e. lack of response from the mobile phone provider after 48 hrs and invalid phone numbers). No major technical errors occurred. All participants who secured financial incentives were paid by direct bank transfer, in line with their stated preference.

Acceptability of intervention components

Overall acceptability ratings and contamination

Table 2 displays intervention satisfaction indicators over the course of the study for participants attending assessments. Overall mean study satisfaction at 12 months was 81%, 77% and 87% for the SMS+I, SMS only and control groups respectively. Satisfaction over time was comparable between both intervention groups. For scores on specific programme attributes see Table 2.

Helpfulness ratings of the narrative texts, webpage and pedometer were highest for the pedometer and relatively stable throughout the measurement time points. At 12 months, helpfulness ratings (out of 5) for the pedometer were 3.9, 4.0, and 3.7 for the SMS+I, SMS only and control groups respectively. The narrative texts (SMS+I=3.4; SMS only=3.3) and the webpage (SMS+I=3.6; SMS only=3.4; control=3.5) were perceived as somewhat helpful on average at 12 months.

Minimal contamination between intervention groups was observed, with one participant at six and 12 months (SMS+I group) and another at 12 months (control group) reporting meeting other men in the study.

--- Table 2 ---

For peer review only - http://bmjopen.bmj.com/site/about/guidelines.xhtml

Acceptability of narrative text messages

Some men sent spontaneous replies to the narrative texts (0-3 months n=25/69, 36%; 3-6 months n=8/69, 12%, 6-12 months n=13/69, 19%; see online supplement 2). Most spontaneous replies were received between 0-3 months (n=370 replies) and decreased at 3-6 months (n=16 replies) and 6-12 months (n=39 replies).

Eleven participants (11/69, 16%) requested to no longer receive texts but asked to remain in the trial. Requests to stop narrative texts were similar in both groups (SMS+I=5/36, 14%; SMS only=6/33, 18%), and occurred throughout the study. Four participants (4/69, 6%) withdrew due to dislike of narrative texts between baseline and three months, two in each intervention group.

Qualitative interviews at three and 12 months demonstrated varied views on the narrative texts. Participants' views ranged from positive to indifferent to negative. Those who liked the narrative texts found the storyline entertaining, engaging and some participants felt a certain camaraderie for the main character:

> *You get involved in the content and you start following the script, they're very funny and me being a [from city name] you can see the funny side and you can see the wit in that, ... and that's what keeps you reading them.* (210010, SMS only, 12m)

For other participants, the frequency of texts became a source of irritation, the storyline did not resonate with their own experience nor could they empathise with the fictional characters:

> *I'm not a lover of chocolate, biscuits, cake, ice cream, I hardly ever touch them, hardly ever, so hearing about somebody eating pizza or sweets means nothing to me because I don't eat them anyway* (120002, SMS+I, 12m)

Some were uncertain of the role of the texts in helping to support weight loss. Others expressed an indifference towards the texts, and a decreasing interest in the story over time leading to infrequent engagement with text content. However, the regular reminder of being a participant in a weight loss programme through receiving texts was seen as important:

> *I often wonder, do they relate to myself here. And the only commonality is both trying to lose weight. So in that respect it's just a reminder all the time, you should be reviewing your weight and watching what you're eating, so…from that it's a positive, yeah.* (220017, SMS+I, 3m)

For peer review only - http://bmjopen.bmj.com/site/about/guidelines.xhtml

A matrix coding query and analysis of the quotations generated from the NVivo qualitative and demographic data suggested no obvious relationships between the participant SIMD level and the qualitative accounts of acceptability of the narrative texts. This is in line with quantitative data showing similar acceptability ratings for overall programme satisfaction and helpfulness ratings of the narrative texts across SIMD levels (see online supplement 3). Some men would have liked more interactivity built into the texts, however, the text message system was set up for uni-directional messaging only.

Acceptability of endowment incentive

At three months, 31/36 SMS+I participants attended the appointment, with eight men losing 5% or more of their baseline weight and securing £50. Attendance at six months was lower (n=23/36), with five participants achieving the weight loss target of >10% weight loss securing £150 each, and a further three participants losing between ≥5-10% of weight securing between £75-150. At 12 months, 23/36 participants attended the appointment, with three achieving >10% weight loss securing £200, and a further seven losing between ≥5-10% of weight securing between £100-200.

Overall, £2,955 was paid in total to the 11 participants (11/36, 31%) successfully meeting or partially meeting weight loss targets. The full £400 was secured by three participants (3/36, 8%). The cost of the incentive per participant was £81.94 (95% CI £34.59-£129.30). All participants who completed the study and secured money at three or six months received it at the end of the study. No participant lost previously secured money due to weight regain to baseline weight at 12 months. One participant secured money at an early appointment but withdrew from the study, did not attend at 12 months, and did not receive any money.

No negative views were expressed about the incentives in qualitative interviews. Only one man reported being motivated by the financial incentive, and many expressed indifference. Participants reported the weight loss and health benefits as sufficiently rewarding.

> *The money's not that much of an issue. I think the incentive for me is the health that, at the end of the day, your health improves. That's the most important incentive. The money, it's fine, it's there, but it's not the incentive* (120022, SMS+I, 3m)

Some interpreted the incentives as a final reward linked to their weight loss targets, whereas others fully understood the intended use of the loss aversion concept:

For peer review only - http://bmjopen.bmj.com/site/about/guidelines.xhtml

> [W]e *all know how much more distress it causes you to lose money than the pleasure of finding a fiver down the back of the couch, you know, so I think if you lose something it almost has a bigger impact on you than if you gain*. (220040, SMS+I, 3m)

Acceptability of study webpages and pedometer

Some participants used the self-monitoring features on the webpage (see online supplement 2) and reported valuing the ability to visually track their weight in interviews. However, most did not access the website. The majority (77%) did not own a pedometer at baseline, and pedometers were highly acceptable to participants.

Acceptability of Control Group

Most control group participants (30/36, 83%) attended the 12 month appointment. Six qualitative interviews were conducted with men in the control group covering topics including acceptability of waiting 12 months for text messages, views about the study and improvement suggestions. Many reported being pleased to be involved in a research study. One participant had a strong negative reaction to being randomised to the control group and subsequently withdrew when invited to the 12 month assessment.

Weight outcomes

Table 3 displays weight change outcomes at three, six, and 12 months. The difference in mean percentage weight loss at 12 months for observed cases was −2.51% (95% CI −6.03 to 1.01) for SMS+I v control and −0.51% (95% CI −3.91 to 2.89) for SMS only v control. The difference in absolute weight loss at 12 months was −2.87kg (95% CI −6.82 to 1.08) for SMS+I v control and −0.57kg (95% CI −4.40 to 3.25) for SMS only v control.

---Table 3 ---

The SMS+I group displayed mean weight loss of over 3% at 12 months (-3.51%, SD=5.83). The SMS only and control groups remained below 3% weight loss on average (SMS only=-1.51%, 4.65; control=-1.00%, SD=5.31). The highest mean percentage weight loss at 12 months for BOCF was -2.24% (SD=4.93) in the SMS+I group, followed by -1.19% (SD=4.16) and -0.80% (SD=4.77) in the SMS only and control groups respectively.

Harms and unintended consequences

No harms or unintended consequences were reported.

For peer review only - http://bmjopen.bmj.com/site/about/guidelines.xhtml

Progression to full trial

An independent study steering committee agreed that the Game of Stones study had demonstrated acceptability and feasibility agreeing that overall the pre-specified progression criteria were sufficiently met to support a full three-arm multi-site RCT.

DISCUSSION

Principal findings

This study successfully recruited 105 men from across the socioeconomic spectrum to a three-armed RCT and overall achieved 74% retention at 12 months' follow-up. Men living in more disadvantaged areas formed 60% of the sample. Narrative texts were broadly acceptable, but some participants disengaged or withdrew from the study due to dislike of texts. The endowment incentives were acceptable, and improving health was reported as a key motivator for weight loss. Positive indicative effects of weight loss were found in intervention and control groups.

Strengths and weaknesses of the study

This study was underpinned by the ROMEO systematic reviews and qualitative evidence synthesis of weight loss interventions for men with obesity[40 41], with a continuing PPI partnership with the Men's Health Forum GB and Ireland charities. Mixed methods research combined quantitative and qualitative data which, together with patient, public and stakeholder involvement, provided a multi-lens perspective on this study.

Most assessments were not blind to group allocation. The difference in assessment schedules for intervention and control groups meant that study staff were only partially blind. A full trial should ensure full blinding of all outcome assessments. Researchers were unable to collect data via qualitative interviews, questionnaires and anthropometric measures with men who withdrew from the study or were lost to follow-up at the 12 month appointment. Those providing a withdrawal reason may have provided socially desirable responses. Text messages were personalised by including the participant's name and weight unit preference, but no tailoring or interactivity were possible due to technical limitations of the delivery system. This feasibility trial was not powered to detect effects on weight loss and, in line with recommendations[27], no p-values are reported. Weight outcomes should be interpreted with caution.

For peer review only - http://bmjopen.bmj.com/site/about/guidelines.xhtml

Relation to other studies

The Game of Stones study adds to the evidence base demonstrating the feasibility of recruiting men for research on sensitive subjects, such as obesity, through community outreach and GP practice lists.[11-13]

The average BMI and age of the study participants of around 35kg/m^2 and 50-55 years is similar to UK weight management trials recruiting in the community[34], primary care[42], or a combination of community and primary care.[12] Three international text message based weight management studies examining outcomes after 12 months recruited younger participants with lower BMI.[43-45] However, three mixed sex weight management studies with financial incentives (two including text message components) reported broadly similar participant demographics to this study.[46-48]

Retention levels of 74% were acceptable and are similar to systematic review evidence of men-only weight loss interventions which found an average retention of 78%, ranging from 44% to 100%.[41] International text message studies with mixed sex participants report similar retention rates at 12 months of 70.3%[44] and 73%[43], with one study conducted in Latvia reporting 93% retention[45]. Two large UK studies recruiting mixed sex participants with obesity to weight management in primary care reported 75%[42] and 81%[49] retention at 12 months.

Two previous narrative text message intervention studies in men targeting alcohol reduction reported high acceptability levels and no negative reactions.[12 13] However, these studies were only 12 weeks in duration and overall contained fewer texts. The narrative texts in this study were broadly acceptable, although some negative reactions were reported. Study withdrawal due to texts and requests to stop texts suggest that the current narrative texts may not be universally acceptable to men. The text messages were designed by a professional scriptwriter/researcher with PPI input from the target population of men from disadvantaged backgrounds, but the narrative texts were similarly acceptable across the socio-economic spectrum. Intervention fidelity was high with the majority of sent texts successfully delivered and no adverse events were encountered, similar to previous text message-based intervention studies.[11-13]

The use of a financial incentive strategy for weight loss in men with obesity across the socioeconomic spectrum was feasible and acceptable. Two previous incentive studies used text messages to inform participants about their incentive achievement of weight loss targets,

For peer review only - http://bmjopen.bmj.com/site/about/guidelines.xhtml

and found it acceptable.[46 47] This extends the evidence base on the use of financial incentives as a complementary behaviour change strategy alongside other components.[22 50]

The financial incentive strategy was designed with future sustainability in mind and to be attractive to public sector funders. The text messages and incentive components are mostly automated, encourage self-management of weight and have a low administrative burden with one bank transfer pay out after 12 months, and verification of weight loss at three, six and 12 months. Yancy et al. similarly provided incentives at three and six months for their six months' incentive intervention[46]. John et al. asked participants to be weighed monthly, for which they received $20 per visit.[47] Few RCTs examine financial incentive strategies that are delivered for at least 12 months.[50] Two other studies providing financial incentives for 12 months provided an intensive financial incentive schedule either weekly[51] or monthly[52]. Previous studies typically provided participants with weekly weight loss goals such as 1lb (0.45kg) per week, on which financial incentives were contingent.[50] Whilst these goals are similar to the weight loss targets in the current study, there were no intermittent or weekly targets. A balance needs to be struck between having more regular weight measurements and more immediate pay-outs, costs and future sustainability.

Patel et al. applied a similar framing which they called a loss incentive[26]. University employees were allocated a monthly hypothetical incentive of $42 upfront and $1.40 was taken away each time their daily goal of 7000 steps was not met. The current study adds to the evidence base demonstrating the acceptability and feasibility of loss framed interventions for weight loss and in settings outside workplaces.

Conclusion

This study tests a novel combination of narrative SMS with endowment incentives that have not previously been combined in this way to address weight loss and weight loss maintenance in men with obesity. This three-arm feasibility trial recruited men from across the socioeconomic spectrum with the majority coming from disadvantaged areas, had an acceptable retention rate, and was broadly acceptable to most participants, and feasible to deliver. Acceptability and feasibility progression criteria were met. A full trial is warranted to determine effectiveness and cost-effectiveness, with consideration of scalability.

For peer review only - http://bmjopen.bmj.com/site/about/guidelines.xhtml

ACKNOWLEDGEMENT

We would like to acknowledge the following people who have contributed to this study:

All men who contributed to PPI and who participated in the study.

Colin Fowler from Men's Health Forum in Ireland, who was involved in the ROMEO systematic review that informed this study, commented on the grant application, protocol and final report. Together with Paula Carroll, they accessed wider PPI in Ireland.

Fiona Hogarth, Director Tayside Clinical Trials Unit, oversaw randomisation, data management and the running of the study from a Clinical Trials Unit perspective.

Hannah Collacott, who was employed as a research assistant at the Health Economics Research Unit, University of Aberdeen in 2016-2017. She undertook the think aloud interviews, PPI and assisted in the design and preliminary analysis of the DCE described in the final report to the funder.(Dombrowski et al., in submission)

Clinical, Community and Academic Networks who advised our team on many aspects of this study, including: NHS Research Scotland Primary Care Network and the GP practices involved, Alliance Scotland, Community Health Councils and Health Centres

The following post-graduate Masters students, who accompanied the research assistants when recruiting men in the community in alphabetical order: Norelle Calder-Macphee, Ines Elenin Lucy Gunatillake, Bryony Lang, Heather Renwick and Amy Spiteri.

Karen Stanton, Lorna Kerr, Karen Murray, Siobhan McDermott, Suzannah Hunter, who provided administrative support.

The prior work of Professor Crombie and his team at Dundee who developed the software to deliver the SMS and assisted with the grant application for this study.(Crombie et al., 2017; Crombie et al., 2018; Irvine et al., 2017)

The Study Steering Committee:

Associate Professor Ed Juszczak, Director, NPEU Clinical Trials Unit, Nuffield Department of Population Health, University of Oxford (Chair).

Professor Kate Jolly, Professor of Public Health & Primary Care, University of Birmingham.

Mr. Mark Kelvin, Programme Director (until 2018), Health and Social Care Alliance Scotland (the ALLIANCE).

For peer review only

Mr. Andrew Shanahan, Director MAN v FAT - the weight loss website for men

Ms. Martine Stead, Deputy Director, Institute for Social Marketing, University of Stirling

Miss Joyce Thompson Dietetic Consultant in Public Health Nutrition and Tayside Nutrition MCN Lead Clinician, NHS Tayside.

AUTHOR CONTRIBUTIONS

SD co-led the study with PH as joint Chief-Investigator and co-led the design and overall conduct of the study and led the writing of the manuscript, overseen by PH.

MM undertook the study set up, recruitment and data collection, co-ordinated various PPI activities, contributed substantially to qualitative and quantitative data analysis and interpretation. MvdP was a co-investigator, contributed to the design and the interpretation of findings and led the DCE and health economic analysis. MG was a co-investigator, contributed to the design and the interpretation of findings and led the development of the narrative SMS component, the related PPI. AA was a co-investigator, contributed to the design and the interpretation of findings. PC and MT linked to PPI networks, contributed to the design, and interpretation of the findings. AE was a co-investigator, contributed to the design and the interpretation of findings and oversaw the statistical analysis. NG and EC contributed to the analysis and interpretation of the qualitative data. CG was a co-investigator, facilitated PPI, contributed to the design, and interpretation of findings. FH was a co-investigator, oversaw the qualitative data collection and analysis and contributed to the interpretation of findings. AH undertook the statistical analysis at Tayside Clinical Trials Unit. CJ was a co-investigator, contributed to the design and the interpretation of findings and oversaw the automated SMS delivery system, recruitment tracking, the study website and integration of data with databases at Tayside Clinical Trials Unit. FK and MMcK were co-investigators and contributed to the design and the interpretation of findings. RS contributed to recruitment, data collection and undertaking qualitative interviews and contributed to the interpretation of findings.

FUNDING

This report presents independent research commissioned by the NIHR. The views and opinions expressed by authors in this publication are those of the authors and do not necessarily reflect those of the NHS, the NIHR, MRC, CCF, NETSCC, the Public Health Research programme or the Department of Health. The views and opinions expressed by the

For peer review only - http://bmjopen.bmj.com/site/about/guidelines.xhtml

interviewees in this publication are those of the interviewees and do not necessarily reflect those of the authors, those of the NHS, the NIHR, MRC, CCF, NETSCC, the Public Health Research programme or the Department of Health.

The Nursing Midwifery and Allied Health Professionals Research Unit, the Health Services Research Unit and Health Economics Research Unit are funded by the Chief Scientist Office of the Scottish Government Health and Social Care Directorate.

COMPETING INTERESTS

Dr Grindle is Director of Eos Digital Health Ltd which holds all rights to DNA - The Digital Narrative Approach deployed to design and write the narrative text intervention.

No other competing interests were declared.

DATA AVAILABILITY

Access to data can be arranged through the Co-PIs of the study: Prof. Pat Hoddinott, (University of Stirling, p.m.hoddinott@stir.ac.uk) and Dr. Stephan Dombrowski (University of New Brunswick, stephan.dombrowski@unb.ca) to discuss data sharing, data requirements and conflicts of interest, in line with any EU and other regulations, including ethics approvals.

For peer review only - http://bmjopen.bmj.com/site/about/guidelines.xhtml

REFERENCES

1. Ng M, Fleming T, Robinson M, et al. Global, regional, and national prevalence of overweight and obesity in children and adults during 1980–2013: a systematic analysis for the Global Burden of Disease Study 2013. *The Lancet* 2014;384(9945):766-81. doi: 10.1016/S0140-6736(14)60460-8

2. The Scottish Government. The Scottish Health Survey 2016. Edinburgh, 2017.

3. Health Survey for England. http://www.hscic.gov.uk/public-health. 2014

4. Robertson C, Archibald D, Avenell A, et al. Systematic reviews of and integrated report on the quantitative, qualitative and economic evidence base for the management of obesity in men. *HTA* 2014;18(35):1-424. doi: 10.3310/hta18350

5. Hall AK, Cole-Lewis H, Bernhardt JM. Mobile Text Messaging for Health: A Systematic Review of Reviews. *Annual Review of Public Health* 2015;36(1):393-415. doi: doi:10.1146/annurev-publhealth-031914-122855

6. Shaw R, Bosworth H. Short message service (SMS) text messaging as an intervention medium for weight loss: A literature review. *Health Informatics Journal* 2012;18(4):235-50. doi: 10.1177/1460458212442422

7. Palmer M, Sutherland J, Barnard S, et al. The effectiveness of smoking cessation, physical activity/diet and alcohol reduction interventions delivered by mobile phones for the prevention of non-communicable diseases: A systematic review of randomised controlled trials. *PLoS ONE* 2018;13(1):e0189801. doi: 10.1371/journal.pone.0189801

8. Ofcom. Adults' Media Use and Attitudes Report. 2016

9. Hinyard LJ, Kreuter MWJHE, Behavior. Using narrative communication as a tool for health behavior change: a conceptual, theoretical, and empirical overview. 2007;34(5):777-92.

10. Dombrowski SU, McDonald M, van der Pol M, et al. Feasibility trial of how to engage men with obesity in SMS (short message system) and incentive interventions for weight loss. in submission

For peer review only - http://bmjopen.bmj.com/site/about/guidelines.xhtml

11. Crombie IK, Irvine L, Falconer DW, et al. Alcohol and disadvantaged men: A feasibility trial of an intervention delivered by mobile phone. *Drug and Alcohol Review* 2017;36(4):468-76. doi: 10.1111/dar.12455

12. Irvine L, Crombie IK, Cunningham KB, et al. Modifying alcohol consumption to reduce obesity: A randomized controlled feasibility study of a complex community-based intervention for men. *Alcohol and Alcoholism* 2017;52(6):677-84. doi: 10.1093/alcalc/agx067

13. Crombie IK, Irvine L, Williams B, et al. Texting to Reduce Alcohol Misuse (TRAM): main findings from a randomized controlled trial of a text message intervention to reduce binge drinking among disadvantaged men. *Addiction* 2018;13(9):1609–18. doi: 10.1111/add.14229

14. Haff N, Patel MS, Lim R, et al. The role of behavioral economic incentive design and demographic characteristics in financial incentive-based approaches to changing health behaviors: A meta-analysis. *Am J Health Promot* 2015;29(5):314-23. doi: 10.4278/ajhp.140714-LIT-333

15. Mantzari E, Vogt F, Shemilt I, et al. Personal financial incentives for changing habitual health-related behaviors: A systematic review and meta-analysis. *Preventive Medicine* 2015;75:75-85. doi: 10.1016/j.ypmed.2015.03.001

16. Jay M, Orstad SL, Wali S, et al. Goal-directed versus outcome-based financial incentives for weight loss among low-income patients with obesity: Rationale and design of the Financial Incentives for Weight Reduction (FIReWoRk) randomised controlled trial. *BMJ Open* 2019;9(4) doi: 10.1136/bmjopen-2018-025278

17. Leahey TM, LaRose JG, Mitchell MS, et al. Small Incentives Improve Weight Loss in Women From Disadvantaged Backgrounds. *American Journal of Preventive Medicine* 2018;54(3):e41-e47. doi: 10.1016/j.amepre.2017.11.007

18. Ryan M, Yi D, Avenell A, et al. Gaining pounds by losing pounds: preferences for lifestyle interventions to reduce obesity. *Health Economics, Policy and Law* 2014;10(2):161-82 doi: 10.1017/S1744133114000413

19. Burns RJ, Donovan AS, Ackermann RT, et al. A theoretically grounded systematic review of material incentives for weight loss: Implications for interventions. *Annals of Behavioral Medicine* 2012;44(3):375-88. doi: 10.1007/s12160-012-9403-4

For peer review only - http://bmjopen.bmj.com/site/about/guidelines.xhtml

20. Giles EL, Robalino S, McColl E, et al. The effectiveness of financial incentives for health behaviour change: Systematic review and meta-analysis. *PLoS ONE* 2014;9(3):e90347. doi: 10.1371/journal.pone.0090347

21. Purnell JQ, Gernes R, Stein R, et al. A systematic review of financial incentives for dietary behavior change. *Journal of the Academy of Nutrition and Dietetics* 2014;114(7):1023-35. doi: 10.1016/j.jand.2014.03.011

22. Ananthapavan J, Peterson A, Sacks G. Paying people to lose weight: the effectiveness of financial incentives provided by health insurers for the prevention and management of overweight and obesity – a systematic review. *Obesity Reviews* 2018;19(5):605-13. doi: 10.1111/obr.12657

23. Paloyo AR, Reichert AR, Reinermann H, et al. The causal link between financial incentives and weight loss: an evidence-based survey of the literature. *Journal of Economic Surveys* 2014;28(3):401-20. doi: 10.1111/joes.12010

24. Camerer CF, Loewenstein G, Rabin M. Advances in Behavioral Economics: Princeton University Press 2003.

25. Cawley J, Downs JS, Loewenstein G. Behavioral Economics and Obesity: Oxford University Press 2012.

26. Patel MS, Asch Md DA, Rosin R, et al. Framing financial incentives to increase physical activity among overweight and obese adults a randomized, controlled trial. *Annals of Internal Medicine* 2016;164(6):385-94. doi: 10.7326/M15-1635

27. Eldridge SM, Chan CL, Campbell MJ, et al. CONSORT 2010 statement: extension to randomised pilot and feasibility trials. *BMJ* 2016;355:i5239. doi: 10.1136/bmj.i5239

28. Eldridge SM, Lancaster GA, Campbell MJ, et al. Defining Feasibility and Pilot Studies in Preparation for Randomised Controlled Trials: Development of a Conceptual Framework. *PLoS One* 2016;11(3):e0150205. doi: 10.1371/journal.pone.0150205 [published Online First: 2016/03/16]

29. O'Cathain A, Murphy E, Nicholl J. Three techniques for integrating data in mixed methods studies. *Bmj* 2010;341:c4587. doi: 10.1136/bmj.c4587 [published Online First: 2010/09/21]

For peer review only - http://bmjopen.bmj.com/site/about/guidelines.xhtml

30. Craig P, Dieppe P, Macintyre S, et al. Developing and evaluating complex interventions: the new Medical Research Council guidance. *Bmj* 2008;337:a1655. doi: 10.1136/bmj.a1655 [published Online First: 2008/10/01]

31. Moore GF, Audrey S, Barker M, et al. Process evaluation of complex interventions: Medical Research Council guidance. *Bmj* 2015;350:h1258. doi: 10.1136/bmj.h1258 [published Online First: 2015/03/21]

32. Teare M, Dimairo M, Shephard N, et al. Sample size requirements to estimate key design parameters from external pilot randomised controlled trials: a simulation study. *Trials* 2014;15(1):264. doi: 10.1186/1745-6215-15-264

33. The Scottish Government. Definition of SIMD quintiles. Edinburgh, 2013.

34. Hunt K, Wyke S, Gray CM, et al. A gender-sensitised weight loss and healthy living programme for overweight and obese men delivered by Scottish Premier League football clubs (FFIT): A pragmatic randomised controlled trial. *The Lancet* 2014;383(9924):1211-21. doi: 10.1016/S0140-6736(13)62420-4

35. Dombrowski SU, Sniehotta FF, Johnston M, et al. Optimizing acceptability and feasibility of an evidence-based behavioral intervention for obese adults with obesity-related co-morbidities or additional risk factors for co-morbidities: An open-pilot intervention study in secondary care. *Patient Educ Couns* 2012;87(1):108-19. doi: 10.1016/j.pec.2011.08.003

36. Malterud K, Siersma VD, Guassora AD. Sample Size in Qualitative Interview Studies: Guided by Information Power. *Qualitative Health Research* 2016;26(13):1753-60. doi: 10.1177/1049732315617444

37. Ritchie J, Spencer L. Qualitative Data Analysis for Applied Policy Research: SAGE 2002.

38. Gamble C, Dudley L, Allam A, et al. Health Services and Delivery Research. An evidence base to optimise methods for involving patient and public contributors in clinical trials: a mixed-methods study. Southampton (UK): NIHR Journals Library 2015.

39. Men's Health Forum. Mind your language: How men talk about mental health. London2018.

For peer review only - http://bmjopen.bmj.com/site/about/guidelines.xhtml

40. Archibald D, Douglas F, Hoddinott P, et al. A qualitative evidence synthesis on the management of male obesity. *BMJ Open* 2015;5(10):e008372. doi: 10.1136/bmjopen-2015-008372

41. Robertson C, Avenell A, Stewart F, et al. Clinical Effectiveness of Weight Loss and Weight Maintenance Interventions for Men: A Systematic Review of Men-Only Randomized Controlled Trials (The ROMEO Project). *Am J Mens Health* 2017;11(4):1096-123. doi: 10.1177/1557988315587550

42. Aveyard P, Lewis A, Tearne S, et al. Screening and brief intervention for obesity in primary care: a parallel, two-arm, randomised trial. *The Lancet* 2016;388(10059):2492-500. doi: 10.1016/S0140-6736(16)31893-1

43. Haapala I, Barengo NC, Biggs S, et al. Weight loss by mobile phone: A 1-year effectiveness study. *Public Health Nutrition* 2009;12(12):2382-91. doi: 10.1017/S1368980009005230

44. Shapiro JR, Koro T, Doran N, et al. Text4Diet: A randomized controlled study using text messaging for weight loss behaviors. *Preventive Medicine* 2012;55(5):412-17. doi: 10.1016/j.ypmed.2012.08.011

45. Silina V, Tessma MK, Senkane S, et al. Text messaging (SMS) as a tool to facilitate weight loss and prevent metabolic deterioration in clinically healthy overweight and obese subjects: a randomised controlled trial. *Scandinavian Journal of Primary Health Care* 2017;35(3):262-70. doi: 10.1080/02813432.2017.1358435

46. Yancy Jr WS, Shaw PA, Wesby L, et al. Financial incentive strategies for maintenance of weight loss: results from an internet-based randomized controlled trial. *Nutrition & Diabetes* 2018;8(1):33. doi: 10.1038/s41387-018-0036-y

47. John LK, Loewenstein G, Troxel AB, et al. Financial incentives for extended weight loss: A randomized, controlled trial. *Journal of General Internal Medicine* 2011;26(6):621-26. doi: 10.1007/s11606-010-1628-y

48. Leahey TM, Subak LL, Fava J, et al. Benefits of adding small financial incentives or optional group meetings to a web-based statewide obesity initiative. *Obesity* 2015;23(1):70-76. doi: 10.1002/oby.20937

49. Little P, Stuart B, Hobbs FR, et al. An internet-based intervention with brief nurse support to manage obesity in primary care (POWeR+): a pragmatic, parallel-group,

For peer review only - http://bmjopen.bmj.com/site/about/guidelines.xhtml

randomised controlled trial. *Lancet Diabetes Endocrinol* 2016;4(10):821-28. doi: 10.1016/S2213-8587(16)30099-7

50. Paul-Ebhohimhen V, Avenell A. Systematic review of the use of financial incentives in treatments for obesity and overweight. *Obesity Reviews* 2008;9(4):355-67. doi: 10.1111/j.1467-789X.2007.00409.x

51. Jeffery RW, Wing RR, Thorson C, et al. Strengthening Behavioral Interventions for Weight Loss: A Randomized Trial of Food Provision and Monetary Incentives. *Journal of Consulting and Clinical Psychology* 1993;61(6):1038-45. doi: 10.1037/0022-006X.61.6.1038

52. Kramer FM, Jeffery RW, Snell MK, et al. Maintenance of successful weight loss over 1 year: Effects of financial contracts for weight maintenance or participation in skills training. *Behavior Therapy* 1986;17(3):295-301. doi: 10.1016/S0005-7894(86)80058-2

For peer review only

Table 1 Participant baseline characteristics

	SMS + I N=36	SMS only N=33	Control N=36	Total N=105
Age (years), mean (SD)	50.9 (14.2)[a]	52.5 (15.1)[b]	53.1 (10.1)	52.2 (13.1)[c]
Waist circumference (cm), mean SD	115.8 (10.0)	114.9 (12.7)	119.5 (12.2)	116.8 (11.8)
Weight (kg), mean (SD)	108.6 (16.4)	107.8 (20.2)	110.7 (19.0)	109.1 (18.4)
Height (cm), mean (SD)	175.9 (6.6)	175.2 (6.7)	173.8 (5.9)	175.0 (6.4)
BMI (kg/m^2), mean (SD)	35.1 (5.3)	35.1 (5.9)	36.7 (6.5)	35.7 (5.9)
**BMI (kg/m^2) categories, n (%)**				
<30	5 (13.9)	3 (9.1)	3 (8.3)	11 (10.5)
≥30->35	16 (44.4)	19 (57.6)	14 (38.9)	49 (46.7)
≥35->40	10 (27.8)	6 (18.2)	7 (19.4)	23 (21.8)
≥40->45	4 (11.1)	2 (6.1)	10 (27.8)	16 (15.2)
≥45->50	0 (0.0)	2 (6.1)	1 (2. 8)	3 (2.9)
≥50	1 (2.8)	1 (3.0)	1 (2. 8)	3 (2.9)
**SIMD deprivation category, n (%)**				
SIMD 1 (most disadvantaged)	11 (31.4)[a]	12 (36.4)	15 (41.7)	38 (36.5)[d]
SIMD 2	8 (22.9)[a]	9 (27.3)	7 (19.4)	24 (23.1)[d]
SIMD 3	6 (17.1)[a]	3 (9.1)	3 (8.3)	12 (11.5)[d]
SIMD 4	4 (11.4)[a]	4 (12.1)	6 (16.7)	14 (13.5)[d]
SIMD 5 (least disadvantaged)	6 (17.5)[a]	5 (15.2)	5 (13.9)	16 (15.4)[d]

For peer review only

For peer review only - http://bmjopen.bmj.com/site/about/guidelines.xhtml

**Marital status, n (%)**				
Cohabiting	4 (11.1)	2 (6.3) [e]	5 (13.8)	11 (10.6)[d]
Divorced	2 (5.6)	0 (0.0) [e]	2 (5.6)	4 (3.9)[d]
Married	22 (61.1)	21 (65.6)[e]	17 (47.2)	60 (57.7)[d]
Separated	1 (2.8)	3 (9.4)[e]	2 (5.6)	6 (5.8)[d]
Single	7 (19.4)	5 (15.6)[e]	8 (22.2)	20 (19.2)[d]
Widowed	0 (0)	1 (3.1)[e]	1 (2.8)	2 (19.2)[d]
Prefer not to say	0 (0.0)	0 (0.0)[e]	1 (2.8)	1 (1.0)[d]
**Comorbidities, n (%)**				
Arthritis	11 (30.6)	3 (9.1)	8 (22.2)	22 (21.0)
Cancer	0 (0.0)	0 (0.0)	1 (2.8)	1 (1.0)
Diabetes	4 (11.1)	5 (15.2)	6 (16.7)	15 (14.3)
Myocardial infarction	2 (5.6)	1 (3.0)	0 (0.0)	3 (2.9)
Hypertension	7 (19.4)	8 (24.2)	7 (19.4)	22 (21.0)
Stroke (including TIA)	1 (2.8)	1 (3.0)	0 (0.0)	2 (1.9)
**Ethnic group, n (%)**				
Asian	2 (5.6)	1 (3.1)[e]	1 (2.8)	4 (3.8)[d]
Black	2 (5.6)	1 (3.1)[e]	1 (2.8)	4 (3.8)[d]
White	32 (88.8)	29 (90.7)[e]	34 (94.4)	95 (91.4)[d]
Prefer not to say	0 (0.0)	1 (3.1) [e]	0 (0.0)	1 (1.0)[d]
**Education**				

For peer review only - http://bmjopen.bmj.com/site/about/guidelines.xhtml

For peer review only

Bachelor Degree (=SVQ5)	7 (19.4)	6 (19.5)[b]	11 (30.6)	24 (23.3)[f]
HNC/ HND (=SVQ4)	4 (11.1)	6 (19.5)[b]	2 (5.6)	12 (11.6)[f]
Higher Grade/ Advanced Higher/ A-Level or equivalent (=SVQ3)	3 (8.3)	5 (16.1)[b]	1 (2.8)	9 (8.7)[f]
Masters/ PhD or equivalent	1 (2.8)	1 (3.2)[b]	3 (8.3)	5 (4.8)[f]
No formal qualifications	7 (19.4)	3 (9.7)[b]	10 (27.8)	20 (19.4)[f]
Standard Grade/GCSE/ Intermediate 1 or 2	6 (16.7)	7 (22.6)[b]	4 (11.1)	17 (16.5)[f]
Still studying	2 (5.6)	1 (3.2)[b]	3 (8.3)	6 (5.8)[f]
Vocational qualifications (=SVQ1+2)	4 (11.1)	1 (3.2)[b]	0 (0.0)	5 (4.8)[f]
Other	0 (0.0)	1 (3.2)[b]	0 (0.0)	1 (1.0)[f]
Prefer not to say	2 (5.6)	0 (0.0)[b]	2 (5.6)	4 (3.9)[f]
**Working status, n (%)**				
Full time student	4 (11.1)	2 (6.3)[e]	1 (2.8)	7 (6.7)[d]
Employed - Full time (30+ hours per week)	16 (44.4)	16 (50.0)[e]	18 (50.0)	50 (48.1)[d]
Employed - Part time (8-29 hours per week)	3 (8.3)	3 (9.4)[e]	0 (0.0)	6 (5.8)[d]
Self-employed	3 (8.3)	3 (9.4)[e]	1 (2.8)	7 (6.7)[d]
Not in paid work	2 (5.6)	2 (6.2)[e]	12 (33.3)	16 (15.4)[d]
Retired	8 (22.2)	6 (18.7)[e]	4 (11.1)	18 (17.3)[d]
Household size, mean (SD)	2.6 (1.3)	2.6 (1.0)	2.4 (1.4)	2.5 (1.3)

For peer review only

Note: [a] N=35, [b] N=31, [c] N=102, [d] N=104, [e] N=32, [f] N=103; BMI = Body Mass Index, CM = centimetres, GCSE = General Certificate of Secondary Education, kg = kilogram, N = overall participants, n = participants within specific category, SD = Standard Deviation, SIMD = Scottish index of multiple deprivation 1 represents the most disadvantaged area; quintile SIMD 5 represents the least disadvantaged area, SVQ = Scottish Vocational Qualification

For peer review only

For peer review only - http://bmjopen.bmj.com/site/about/guidelines.xhtml

Table 2 Programme satisfaction, and contamination at three, six and 12 months

	3 months		6 months		12 months		
	SMS + I (N=31)	SMS only (N=27)	SMS + I (N=23)	SMS only (N=21)	SMS + I (N=22)	SMS only (N=26)	Control (N=30)
Programme satisfaction (0-100), mean (SD)	80.3 (21.1)	75.0 (22.2)	76.2 (29.6)	79.0 (20.7)	80.9 (20.0)	77.0 (20.8)	87.3 (17.5)[a]
**Programme has been… (1 = low, 5 = high), mean (SD)**							
understandable	4.5 (0.9)	4.4 (0.8)[b]	4.4 (1.2)[c]	4.4 (1.2)	4.6 (1.1)[d]	4.6 (1.1)[e]	4.6 (0.9)[a]
useful	4.4 (0.9)	4.0 (1.0)	4.1 (1.3)	4.1 (1.1)[d]	4.1 (1.2)	4.0 (1.0)	4.3 (0.9)
helpful	4.3 (0.9)[f]	3.9 (1.0)	4.1 (1.3)	4.1 (1.1)[d]	4.1 (1.10)	4.0 (1.0)	4.3 (0.9)
interesting	4.3 (1.0)	4.2 (1.1)	4.2 (1.1)	4.4 (1.3)[d]	4.3 (0.8)	4.3 (1.2)	4.5 (1.0)
relevant	4.2 (1.1)	3.9 (1.2)	4.0 (1.3)	4.3 (1.1)[d]	4.1 (1.1)	4.2 (1.1)	4.5 (1.0)
**Helpfulness (1 = low, 5 = high), mean (SD)**							
Text messages	3.4 (1.5)	3.1 (1.3)	3.1 (1.3)	3.6 (1.3)	3.4 (1.3)	3.3 (1.4)	n/a

For peer review only - http://bmjopen.bmj.com/site/about/guidelines.xhtml

Website	3.3 (1.2)[g]	3.3 (0.9)[b]	3.3 (1.2)	3.2 (0.8)[d]	3.6 (1.0)	3.4 (0.7)[h]	3.5 (1.1) [b]
Pedometer	4.1 (1.2)[f]	4.1 (0.9)	3.7 (1.4)	4.0 (1.0)	3.9 (1.2)[i]	4.0 (1.0)[e]	3.7, (1.4)
**Met other men in programme, n (%)**							
Yes	0 (0.0)	0 (0.0)	1 (4.3)	0 (0.0)	1 (4.5)	0 (0.0)	1 (3.3)

Note. [a] N=29, [b] N=26, [c] N=22, [d] N=20, [e] N=25, [f] N=30, [g] N=28, [h] N=24, SD = Standard Deviation.

For peer review only - http://bmjopen.bmj.com/site/about/guidelines.xhtml

Table 3 Weight change at three, six, and 12 months

	3 months		6 months		12 months		
	SMS + I N=36	SMS only N=33	SMS + I N=36	SMS only N=33	SMS + I N=36	SMS only N=33	Control N=36
**Weight change (kg), mean (SD)**							
Observed cases only	2.79 (3.50)[a]	-1.97 (3.97)[b]	-4.59 (5.62)[c]	-3.30 (4.92)[d]	-3.93 (5.74)[c]	-1.64 (5.64)[b]	-1.06 (6.29)[e]
BOCF	-2.40 (3.38)	-1.55 (3.60)	-2.93 (4.98)	-2.10 (4.21)	-2.51 (4.94)	-1.29 (5.03)	-0.86 (5.64)
LOCF	-2.40 (3.38)	-1.86 (3.88)	-3.38 (4.92)	-2.21 (4.39)	-2.98 (4.91)	-1.33 (5.04)	-0.86 (5.64)
**Weight change (%), mean (SD)**							
Observed cases only	-2.54 (3.47)[a]	-1.95 (3.72)[b]	-4.20 (5.54)[c]	-3.02 (4.22)[d]	-3.51 (5.83)[c]	-1.51 (4.65)[b]	-1.00 (5.31)[e]
BOCF	-2.18 (3.33)	-1.53 (3.39)	-2.69 (4.84)	-1.92 (3.65)	-2.24 (4.93)	-1.19 (4.16)	-0.80 (4.77)
LOCF	-2.18 (3.33)	-1.53 (3.39)	-3.11 (4.80)	-2.06 (3.85)	-2.68 (4.92)	-1.22 (4.16)	-0.80 (4.77)
**Weight change categories (observed cases only), n (%)**							

weight gain	6 (19.4)[a]	8 (30.8)[b]	2 (8.7)[c]	6 (28.6)[d]	5 (21.7)[c]	10 (38.5)[b]	14 (48.3)[e]
0-<3% weight loss	13 (41.9)[a]	10 (38.5)[b]	10 (43.5)[c]	7 (33.3)[d]	5 (21.7)[c]	9 (34.6)[b]	7 (24.1)[e]
≥3-<5% weight loss	4 (12.9)[a]	4 (15.4)[b]	3 (13.0)[c]	2 (9.5)[d]	3 (13.0)[c]	4 (15.4)[b]	0 (0.0)[e]
≥5-<10% weight loss	8 (25.8)[a]	2 (7.7)[b]	3 (13.0)[c]	4 (19.1)[d]	7 (30.4)[c]	2 (7.7)[b]	6 (20.7)[e]
≥10% weight loss	0 (0.0)[a]	2 (7.7)[b]	5 (21.7)[c]	2 (9.5)[d]	3 (13.0)[c]	1 (3.9)[b]	2 (6.9)
**BMI change, mean (SD)**							
Observed cases only	-0.89 (1.1) [a]	-0.73 (1.32)[b]	-1.45 (1.79)[c]	-1.04 (1.49)[d]	-1.24 (1.89)[c]	-0.49 (1.68)[b]	-0.47 (2.13)[e]
BOCF	-0.75 (1.07)	-0.59 (1.22)	-0.92 (1.58)	-0.66 (1.28)	-0.78 (1.60)	-0.37 (1.50)	-0.37 (1.96)
LOCF	-0.75 (1.07)	-0.59 (1.22)	-0.93 (1.56)	-0.69 (1.34)	-0.93 (1.59)	-0.38 (1.50)	-0.37 (1.96)
**Waist circumference change (cm), mean (SD)**							
Observed cases only	-3.74 (4.62) [a]	-3.14 (4.3)[b]	-4.70 (6.29)[c]	-3.14 (4.34)[d]	-4.40 (6.08)[c]	-2.30 (4.37)[b]	-2.26 (4.97)[e]
BOCF	-3.22 (4.47)	-2.49 (3.9)	-3.00 (5.49)	-2.00 (3.76)	-2.81 (5.82)	-1.82 (3.98)	-1.82 (4.53)
LOCF	-3.22 (4.47)	-2.49 (3.9)	-3.71 (5.76)	-2.23 (4.03)	-3.51 (4.48)	-1.85 (3.97)	-1.82 (4.53)

For peer review only - http://bmjopen.bmj.com/site/about/guidelines.xhtml

Note. [a] N=31, [b] N=26, [c] N=23, [d] N=21, BOCF = baseline observation carried forward, cm = centimetres, LOCF = last observation carried forward, kg = kilogram, M = mean, SD = Standard Deviation.

For peer review only

Figure 1: Consort flow diagram

For peer review only

4.

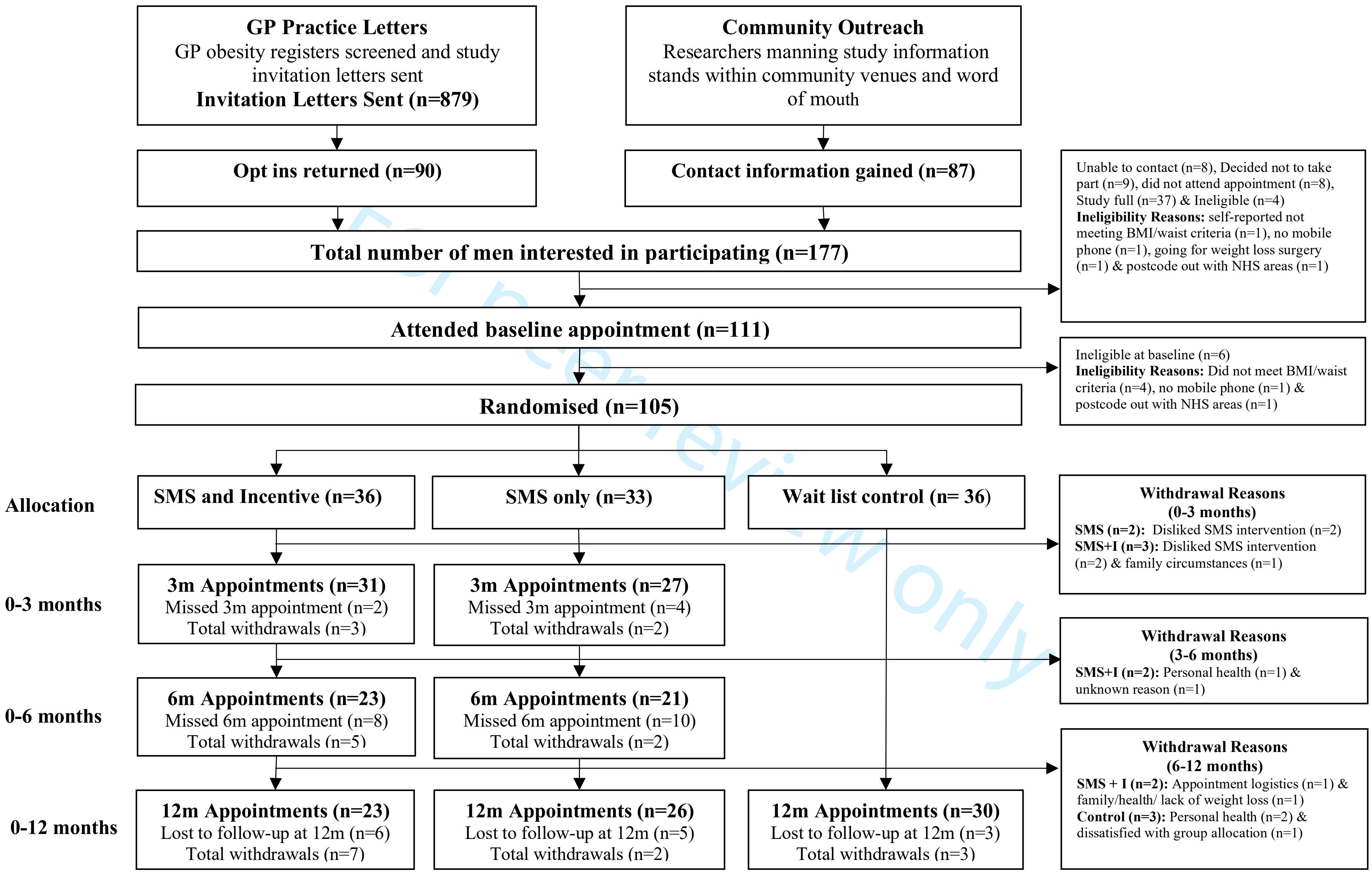

For peer review only - http://bmjopen.bmj.com/site/about/guidelines.xhtml

Online supplement 1: Incentive strategy contingent on meeting weight loss targets over 12 months

	Money secured	Money lost
3 months: £50 – target 5% weight loss		
<5%	£0	£50
≥5%	£50	£0
6 months: £150 – target 10%		
<5%	£0	£150
5%	£75	£75
6%	£90	£60
7%	£105	£45
8%	£120	£30
9%	£135	£15
≥10%	£150	£0
12 months: £200 – target 10%		
<5%	£0	£200
5%	£100	£100
6%	£120	£80
7%	£140	£60
8%	£160	£40
9%	£180	£20
≥10%	£200	£0

For peer review only - http://bmjopen.bmj.com/site/about/guidelines.xhtml

Online supplement 2 Intervention engagement over time for narrative texts and study webpage

	0-3 months		3-6 months		6-12 months	
	SMS + I N=36	SMS only N=33	SMS + I N=36	SMS only N=33	SMS + I N=36	SMS only N=33
Replies to texts, count	289	81	10	6	28	11
Replies to texts, mean (SD)	8.0 (24.2)	2.5 (7.9)	0.3 (0.7)	0.3 (1.0)	0.8 (3.3)	0.3 (1.0)
**Text replies, n (%)**						
0 replies	21 (58.3)	23 (69.7)	30 (83.3)	31 (93.9)	28 (77.8)	28 (84.8)
1-5 replies	9 (25.0)	7 (22.3)	6 (16.7)	2 (6.1)	7 (19.4)	5 (15.2)
6-10 replies	2 (5.6)	1 (3.0)	0 (0.0)	0 (0.0)	0 (0.0)	0 (0.0)
11-15 replies	0 (0.0)	0 (0.0)	0 (0.0)	0 (0.0)	0 (0.0)	0 (0.0)
16-20 replies	1 (2.8)	1 (3.0)	0 (0.0)	0 (0.0)	0 (0.0)	0 (0.0)
>20 replies	3 (8.3)	1 (3.0)	0 (0.0)	0 (0.0)	1 (2.8)	0 (0.0)

For peer review only - http://bmjopen.bmj.com/site/about/guidelines.xhtml

Stopping texts, n (%)	1 (2.8)	2 (6.1)	3 (8.3)	3 (9.1)	1 (2.8)	1 (3.0)
Withdrawal due to texts (self-report)	2 (5.6)	2 (6.1)	0 (0.0)	0 (0.0)	0 (0.0)	0 (0.0)
**Participants using website self-monitoring features, n (%)**						
Weight	8 (22.2)	5 (15.2)	6 (16.7)	3 (9.1)	4 (11.1)	3 (9.1)
Steps	10 (27.8)	6 (18.2)	7 (19.4)	2 (6.1)	3 (8.3)	2 (6.1)
Waistline	4 (11.1)	3 (9.1)	3 (8.3)	0 (0.0)	1 (2.8)	0 (0.0)
Belt notches	2 (5.6)	0 (0.0)	2 (5.6)	0 (0.0)	0 (0.0)	0 (0.0)
**Number self-monitoring features used, n (%)**						
None	24 (66.7)	26 (78.8)	28 (77.8)	30 (90.1)	31 (86.1)	29 (87.9)
One	5 (13.9)	2 (6.1)	2 (5.6)	1 (3.1)	3 (8.3)	3 (9.1)
Two	3 (8.3)	3 (9.1)	3 (8.3)	2 (6.1)	1 (2.8)	1 (3.0)

For peer review only

Three	3 (8.3)	2 (6.1)	2 (5.6)	0 (0.0)	1 (2.8)	0 (0.0)
Four	1 (2.8)	0 (0.0)	1 (2.8)	0 (0.0)	0 (0.0)	0 (0.0)
**Number of entries on self-monitoring feature, count**						
Weight	69	115	45	23	42	46
Steps	419	296	350	102	397	74
Waistline	4	7	5	0	2	0
Belt notches	8	0	4	0	0	0

Note. M = mean, SD = Standard Deviation.

For peer review only

Online supplement 3 Satisfaction and helpfulness ratings for overall programme and narrative texts at 12 months by deprivation status

	SMS+I		SMS only		Total	
	SIMD 1, 2 (n=14)	SIMD 3+ (n=7)	SIMD 1, 2 (n=16)	SIMD 3+ (n=10)	SIMD 1, 2 (n=30)	SIMD 3+ (n=17)
Satisfaction programme[a]	81.8, 21.7	79.9, 19.2	74.6, 23.5	81.0, 16.0	77.9, 22.6	80.5, 16.8
Helpfulness programme[b]	4.2, 1.1	3.9, 1.1	3.9, 1.1	4.2, 1.0	4.1, 1.1	4.1, 1.0
Helpfulness of narrative texts[b]	3.4, 1.3	3.4, 1.4	3.0, 1.5	3.8, 1.2	3.2, 1.4	3.6, 1.3

Note. All data mean (SD), [a] scored 0-100 (higher = more satisfied), [b] scored 1-5 **(**1 = low, 5 = high**),** SIMD = Scottish index of multiple deprivation 1 represents the most disadvantaged area; quintile SIMD 5 represents the least disadvantaged area SMS = Short message service, SMS+I = SMS plus incentive group.

For peer review only

CONSORT 2010 checklist of information to include when reporting a pilot or feasibility trial*

Section/Topic	Item No	Checklist item	Reported on page No
**Title and abstract**			
	1a	Identification as a pilot or feasibility randomised trial in the title	1
	1b	Structured summary of pilot trial design, methods, results, and conclusions (for specific guidance see CONSORT abstract extension for pilot trials)	3, 4
**Introduction**			
Background and objectives	2a	Scientific background and explanation of rationale for future definitive trial, and reasons for randomised pilot trial	6, 7
	2b	Specific objectives or research questions for pilot trial	7
**Methods**			
Trial design	3a	Description of pilot trial design (such as parallel, factorial) including allocation ratio	7
	3b	Important changes to methods after pilot trial commencement (such as eligibility criteria), with reasons	n/a
Participants	4a	Eligibility criteria for participants	8
	4b	Settings and locations where the data were collected	8,9
	4c	How participants were identified and consented	8,9
Interventions	5	The interventions for each group with sufficient details to allow replication, including how and when they were actually administered	10, 11
Outcomes	6a	Completely defined prespecified assessments or measurements to address each pilot trial objective specified in 2b, including how and when they were assessed	11-13
	6b	Any changes to pilot trial assessments or measurements after the pilot trial commenced, with reasons	n/a
	6c	If applicable, prespecified criteria used to judge whether, or how, to proceed with future definitive trial	7
Sample size	7a	Rationale for numbers in the pilot trial	8
	7b	When applicable, explanation of any interim analyses and stopping guidelines	n/a
Randomisation:			
Sequence generation	8a	Method used to generate the random allocation sequence	9
	8b	Type of randomisation(s); details of any restriction (such as blocking and block size)	9
Allocation concealment mechanism	9	Mechanism used to implement the random allocation sequence (such as sequentially numbered containers), describing any steps taken to conceal the sequence until interventions were assigned	9

For peer review only

Implementation	10	Who generated the random allocation sequence, who enrolled participants, and who assigned participants to interventions	9
Blinding	11a	If done, who was blinded after assignment to interventions (for example, participants, care providers, those assessing outcomes) and how	9
	11b	If relevant, description of the similarity of interventions	n/a
Statistical methods	12	Methods used to address each pilot trial objective whether qualitative or quantitative	11-13
**Results**			
Participant flow (a diagram is strongly recommended)	13a	For each group, the numbers of participants who were approached and/or assessed for eligibility, randomly assigned, received intended treatment, and were assessed for each objective	40
	13b	For each group, losses and exclusions after randomisation, together with reasons	40
Recruitment	14a	Dates defining the periods of recruitment and follow-up	15
	14b	Why the pilot trial ended or was stopped	15
Baseline data	15	A table showing baseline demographic and clinical characteristics for each group	31-33
Numbers analysed	16	For each objective, number of participants (denominator) included in each analysis. If relevant, these numbers should be by randomised group	31-45
Outcomes and estimation	17	For each objective, results including expressions of uncertainty (such as 95% confidence interval) for any estimates. If relevant, these results should be by randomised group	15-19
Ancillary analyses	18	Results of any other analyses performed that could be used to inform the future definitive trial	n/a
Harms	19	All important harms or unintended effects in each group (for specific guidance see CONSORT for harms)	19
	19a	If relevant, other important unintended consequences	n/a
**Discussion**			
Limitations	20	Pilot trial limitations, addressing sources of potential bias and remaining uncertainty about feasibility	20
Generalisability	21	Generalisability (applicability) of pilot trial methods and findings to future definitive trial and other studies	22
Interpretation	22	Interpretation consistent with pilot trial objectives and findings, balancing potential benefits and harms, and considering other relevant evidence	22
	22a	Implications for progression from pilot to future definitive trial, including any proposed amendments	22
**Other information**			
Registration	23	Registration number for pilot trial and name of trial registry	4
Protocol	24	Where the pilot trial protocol can be accessed, if available	7
Funding	25	Sources of funding and other support (such as supply of drugs), role of funders	24,25
	26	Ethical approval or approval by research review committee, confirmed with reference number	7

For peer review only

Citation: Eldridge SM, Chan CL, Campbell MJ, Bond CM, Hopewell S, Thabane L, et al. CONSORT 2010 statement: extension to randomised pilot and feasibility trials. BMJ. 2016;355.
*We strongly recommend reading this statement in conjunction with the CONSORT 2010, extension to randomised pilot and feasibility trials, Explanation and Elaboration for important clarifications on all the items. If relevant, we also recommend reading CONSORT extensions for cluster randomised trials, non-inferiority and equivalence trials, non-pharmacological treatments, herbal interventions, and pragmatic trials. Additional extensions are forthcoming: for those and for up to date references relevant to this checklist, see www.consort-statement.org.

For peer review only
